# Unveiling medication patterns in traditional Chinese medicine for the prevention of colorectal cancer recurrence: from potential combinations to validation of components and targets

**DOI:** 10.1186/s13020-026-01438-5

**Published:** 2026-06-04

**Authors:** Qianqian Bu, Shaoxun Yuan, Xiaoman Wei, Junyi Wang, Liu Li, Pan Chen, Weixing Shen, Dongdong Sun, Lingyu Linda Ye, Yun Yang, Luying Xu, Sicheng Lu, Dayue Darrel Duan, Haibo Cheng

**Affiliations:** 1 The First Clinical Medical College, Nanjing University of Chinese Medicine, Nanjing, 210023 China; 2 Jiangsu Collaborative Innovation Center of Traditional Chinese Medicine in Prevention and Treatment of Tumor, Nanjing, 210023 China; 3 School of Artificial Intelligence and Information Technology, Nanjing University of Chinese Medicine, Nanjing, 210023 China; 4 Jiangsu Province Engineering Research Center of TCM Intelligence Health Service, Nanjing University of Chinese Medicine, Nanjing, 210023 China; 5 Affiliated Hospital of Nanjing University of Chinese Medicine, Nanjing, 210029 China; 6 The Academy of Phenomics of Traditional Chinese Medicine, Nanjing University of Chinese Medicine, Nanjing, 210030 China; 7 School of Integrative Medicine of Nanjing University of Chinese Medicine, Nanjing, 210023 China; 8 Research Institute of TCM Zhenghou Phenomics, School of Traditional Chinese Medicine, Hunan University of Chinese Medicine, Changsha, 410208 China; 9 Department of Stomatology, Nanjing Drum Tower Hospital, Nanjing, 210008 China; 10 Department of Pharmacology, University of Nevada Reno School of Medicine, Reno, NV 89557 USA

**Keywords:** Colorectal cancer, Traditional Chinese medicine, Recurrence, Network analysis, Compatibility rules

## Abstract

Colorectal cancer (CRC) is a prevalent malignant tumor with high incidence and mortality rates, with recurrence being the primary cause of death among patients. Traditional Chinese Medicine (TCM) utilizes a holistic cognitive approach to develop herbal treatment strategies, displaying significant efficacy in slowing tumor progression and improving patients' quality of life. However, discrepancies in herbal prescription strategies arise from variations in clinicians’ syndrome differentiation and experiential knowledge, leading to a lack of systematic understanding of compound prescription compatibility rules. This study aims to identify TCM prescriptions that are effective in preventing or treating CRC recurrence through evidence-supported and clinically applied prescriptions. By employing Apriori association rule mining and graph convolutional network analysis, a core herb spectrum associated with anti-recurrence prescriptions was identified, emphasizing a high-confidence herbal combination for further mechanistic exploration. Subsequent network pharmacology analysis revealed quercetin and kaempferol as representative active compounds with PTGS2 identified as a potential key target. Molecular docking, molecular dynamics simulation, public database analysis, and in vitro experiments provided initial evidence supporting their interaction and biological effects in CRC cells. Overall, this study reveals the compatibility characteristics of TCM prescriptions for preventing CRC recurrence and offers a scientific foundation for the continued use of TCM-based strategies in managing CRC recurrence.

**Supplementary Information:**

The online version contains supplementary material available at 10.1186/s13020-026-01438-5.

## Introduction

Colorectal cancer (CRC) is one of the most prevalent malignant tumors worldwide, characterized by high incidence and mortality rates that severely threaten human life and health [1]. Despite significant advancements in surgical techniques and drug therapies, recurrence following curative resection and anti-cancer therapies treatments remains a major challenge in CRC management. Current epidemiological data indicate that approximately 20% of patients experience tumor recurrence within 5 years after radical surgery, with stage-dependent recurrence rates of 6.8–16.3% for stage I, 11.6–21.9% for stage II, and 24.6–35.3% for stage III disease [2]. These persistent recurrence rates underscore an urgent need for more effective therapeutic strategies to improve long-term outcomes in CRC patients.

Traditional Chinese Medicine (TCM) has gained increasing global recognition as a complementary approach in oncology, attributed to its favorable safety profile characterized by low toxicity, minimal side effects, and excellent patient tolerability. Unlike conventional cancer therapies that primarily focus on tumor cell eradication, TCM adopts a holistic treatment paradigm emphasizing systemic regulation. This approach aims to simultaneously modulate disease progression and enhance patients’ quality of life. Notably, accumulating evidence supports a multifaceted role of TCM in cancer prevention and treatment, including chemopreventive potential against tumorigenesis, mitigation of treatment-related adverse effects, enhancement of the efficacy of radiotherapy or chemotherapy, and reduction of tumor recurrence and metastasis rates [3, 4].

However, the clinical application of TCM formulas faces considerable challenges due to their inherent complexity. Prescription methodologies heavily rely on clinicians personalized syndrome differentiation and experiential knowledge, leading to substantial variability in herbal combinations. This lack of standardization not only hinders the systematic advancement of TCM formulations but also poses significant barriers to establishing robust evidence-based efficacy through reproducible clinical trials.

Randomized controlled trials (RCTs) are regarded as the gold standard for validating the clinical application of TCM formulas. Concurrently, commercially available Chinese patent medicines and patented TCM combinations offer real-world corroboration of clinical efficacy. The above encompass valuable implicit prescription patterns that encapsulate clinically pertinent therapeutic strategies. The application of advanced data mining techniques facilitates the systematic extraction and analysis of these patterns through multidimensional evaluation of TCM formulations. Recent research on TCM formulations has shifted towards data-driven modeling and predictive analytics. Methodological advancements highlight the importance of machine learning [5], knowledge graphs [6] and deep learning [7, 8] in modernizing TCM research, particularly in integrating diverse data types and analyzing complex relationships among herbs, compounds, targets, and therapeutic outcomes. Furthermore, progress in artificial intelligence is paving the way for more autonomous and sophisticated analytical frameworks in TCM research. Various computational approaches offer complementary perspectives on formula compatibility, classic data mining methods such as the Apriori association rule and graph convolutional network (GCN) demonstrating wide applicability in TCM-research. Specifically, the Apriori association rules have been proven to be effectively applicable in various research scenarios such as mining acupuncture point combinations [9], analyzing TCM prescription [10], and extracting characteristic features of TCM syndromes [11]. GCN can aggregate features from formula nodes and TCM nodes to investigate correlations between formula composition and efficacy. GCN can also address the challenge of small sample sizes by utilizing topological information, even in datasets with limited samples [7, 12]. These computational approaches offer complementary insights into the intricate compatibility principles of TCM formulations, facilitating the translation from empirical knowledge to standardized clinical applications.

**Fig.1:**
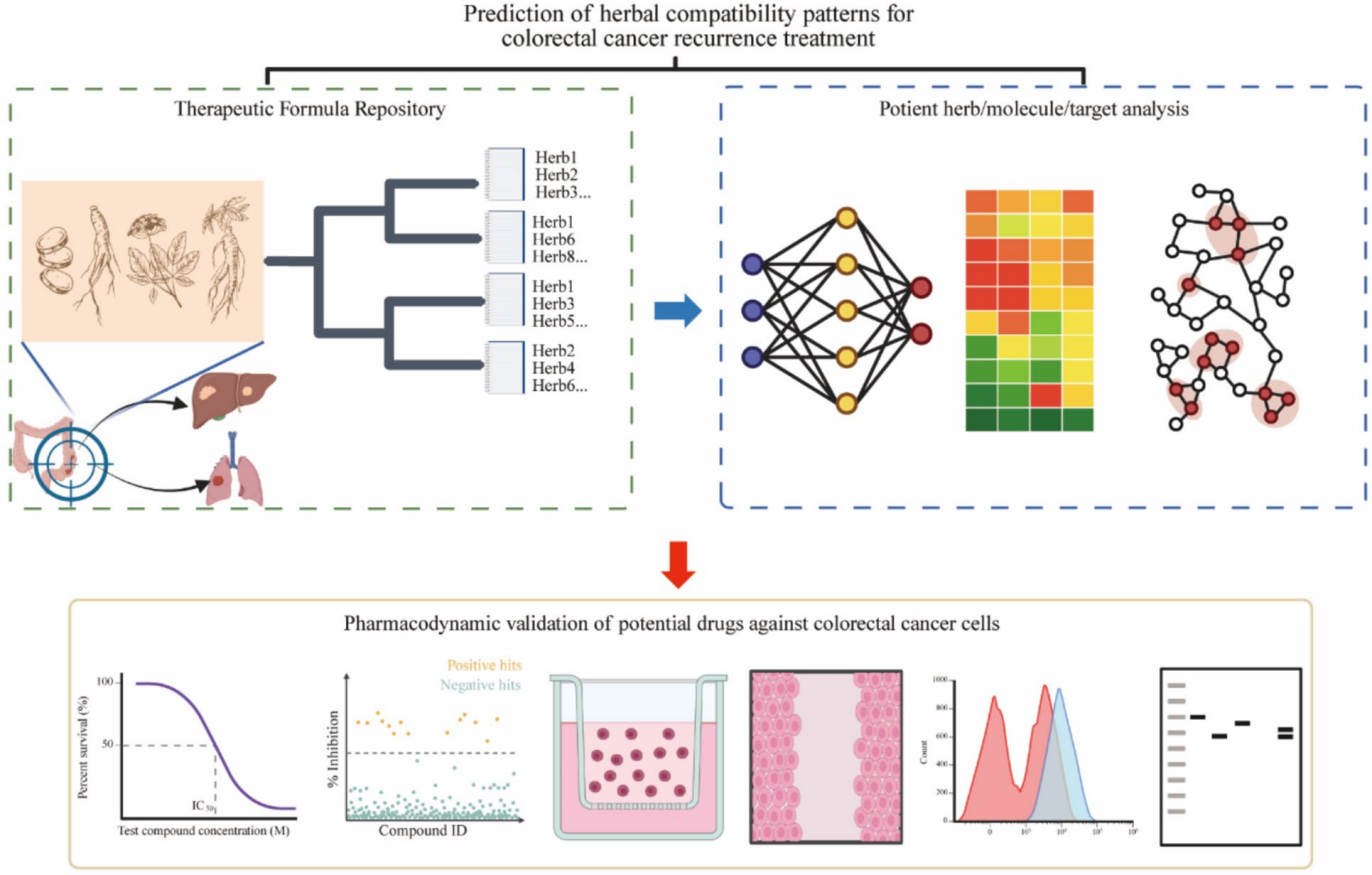
Schematic diagram of this study

This study aimed to determine the fundamental herb combination in TCM formulations for preventing CRC recurrence. This was accomplished by integrating bioinformatics methodologies, evidence-based research and clinical prescriptions. To improve the reliability of candidate selection, anti-recurrence prescriptions were contrasted with general treatment prescriptions, and essential herbs were identified through frequency analysis, random forest, Apriori association rule mining, and GCN analysis. Utilizing this combination derived from prescriptions, network pharmacology was employed to methodically screen significant active components and molecular targets, with potential mechanisms further explored through molecular docking, molecular dynamics simulation, analysis of public database, and in vitro experiments. The objective of this approach was to elucidate the scientific rationale behind TCM combinations and bolster the clinical application of TCM in managing CRC recurrence (Fig. 1).

## Materials and methods

### Data collection and filtering

Prescriptions were collected from four sources up to December 31, 2024: (1) Registered RCTs from the establishment of the Chinese Clinical Trial Registry (ChiCTR), Clinical Trials, and the International Traditional Medicine Clinical Trial Registry (ITMCTR); (2) National Basic Medical Insurance, Work-related Injury Insurance, and Maternity Insurance Drug List (2024); (3) Patents from the China National Intellectual Property Administration; (4) Published CRC-related RCTs retrieved from CNKI, Wanfang, VIP, and PubMed.

TCM prescriptions explicitly mentioning the prevention and treatment of CRC recurrence, showing anti-tumor effects in labeling, or demonstrating anti-recurrence efficacy in clinical trials were included in the anti-recurrence set. Prescriptions intended for precancerous lesions, perioperative treatment, or adjuvant therapy to enhance efficacy and reduce toxicity, but not specifically aimed at recurrence prevention, were categorized as “general treatment prescriptions”. TCM formulas with incomplete composition descriptions and withdrawn registered trials were excluded; duplicate formulas with identical compositions were counted once. Recurrence in this study encompassed both local and distant recurrence.

The above data were independently extracted by two researchers, who then cross-checked each other’s work. All herb names in this study have been standardized by HERB 2.0 database and are consistent with those recorded in the Pharmacopoeia of the People's Republic of China (2020 Edition).

### Apriori association rule mining

The apriori association rule mining approach was applied to identify frequent itemsets and co-prescription patterns in TCM formulas. The analysis was conducted using the R package *arules* (v4.2.3) with a minimum support of 0.08 and a minimum confidence of 0.80. The rules were ranked by lift, with the highest-lift combinations retained. These thresholds were chosen to balance retention of clinically meaningful low-frequency combinations with exclusion of sporadic, non-reproducible associations.

### Graph convolutional network

This network was used to prevent recurrence and metastasis of CRC, involving 216 distinct herbs. An undirected edge was established between any two herbs that appeared together in the same formula, with an edge weight equal to their co-occurrence frequency. This network was encoded as a PyTorch Geometric graph object, using node degree as the initial feature. Drug embeddings were learned via a two-layer GCN with hidden and output dimensions of 32 and 16, respectively. The model was trained for 200 epochs on a CUDA-enabled device using Adam optimization (learning rate = 0.01) under a self-supervised link prediction objective. The resulting 16-dimensional embeddings were clustered by K-means (K = 7, selected by maximizing the silhouette score), and key herb clusters were prioritized based on a composite metric incorporating average node degree, intra-cluster subgraph density, and the proportion of formulas covered. All analyses were conducted in Python 3.9.23, utilizing PyTorch 2.6.0 + cu124, PyTorch Geometric, scikit-learn (≥ 1.4), and NetworkX 3.3.

### Enrichment analysis and random forest model construction

The study calculated the frequency of herbs in prescriptions for anti-CRC recurrence and general treatment, comparing the differences between the two groups. Frequency variance > 20%, 10–20%, and 0–10% were defined as high, moderate, and slight, respectively. Additionally, a random forest classifier (ntree = 1000, set. seed = 123) was developed to identify differential herbs, with herbs as predictors and group labels as outcomes. Model performance was evaluated by the out-of-bag (OOB) error, and herb importance was determined using mean decrease gini and mean decrease accuracy.

### Network pharmacology analysis

Active compounds and corresponding targets were retrieved from the Traditional Chinese Medicine Systems Pharmacology platform (TCMSP), Herb Ingredients' Targets (HIT), Integrated Traditional Chinese Medicine (ITCM), and Traditional Chinese Medicine Bank (TCMBank) databases, with data processing and network construction tailored to each platform. In TCMSP [13], compounds were filtered using oral bioavailability (OB) ≥ 30% and drug-likeness (DL) ≥ 0.18. Targets required SVM_score ≥ 0.8 and RF_score ≥ 0.7. Targets were mapped to official gene symbols using *STRING* (v12.0) to construct a protein–protein interaction (PPI) network, visualized in Cytoscape (3.7.2) and R *igraph* package (v2.1.4). In HIT [14], compounds lacking annotated targets were excluded. In ITCM [15], only compounds with valid DL scores and PubChem compound identifiers (CIDs) were retained, and the CIDs were used for target mapping. In TCMBank [16], all recorded compounds were included after removing entries with unstandardized chemical or gene names. For HIT, ITCM, and TCMBank, compound–target bipartite networks were constructed, and node degree was used to identify key active compounds and central targets.

### Gene ontology (GO) and kyoto encyclopedia of genes and genomes (KEGG) enrichment analysis

GO and KEGG enrichment were performed using the R packages *clusterProfiler* (v4.6.2), *org.Hs.eg.db* (v3.16.0), and *ggplot2* (v3.5.2). For enrichment analysis, the parameter pAdjustMethod in *clusterProfiler* was set to “fdr” (false discovery rate) to control the false positive rate caused by multiple hypothesis testing. Bubble plots were generated to visualize pathway significance and gene counts, while heatmaps were used to compare enrichment patterns of different gene sets across GO terms and KEGG pathways.

### Molecular docking

Simulations of protein–ligand complex predictions have been performed using AutoDock, respectively with the following PDB structures: PTGS1 (1PGE), PTGS2 (1CX2), NCOA2 (1M2N), HSP90AA1 (1YET), PRSS1 (1A0J), and GABRA1 (4COF). Proteins were prepared in PyMOL by stripping water molecules and native ligands, followed by the addition of polar hydrogens. The ligand structures of quercetin (CID: 5280343) and kaempferol (CID: 5280863) were retrieved from PubChem, converted to MOL2 format via Open Babel, and subjected to energy minimization using Avogadro. For each protein–ligand pair, three independent runs were performed, retaining the conformation with the lowest binding energy for subsequent analysis.

### Molecular dynamics simulation

Molecular dynamics simulations were performed in GROMACS 2022.3, using the CHARMM36m force field for protein and CGenFF for ligands. Each complex was solvated in a cubic box with the TIP3P water model and neutralized with 0.15 M NaCl. Systems were energy-minimized via the steepest descent algorithm until the maximum force was < 1000 kJ·mol^−1^·nm^−1^ (up to 50,000 steps). Subsequently, 100 ps of NVT (300 K) and 100 ps of NPT (1 bar) equilibrations were conducted. Finally, a 1 ns production simulation was run with a 2 fs time step, saving coordinates every 10 ps. Hydrogen bonds were constrained using the LINCS algorithm, and long-range electrostatic interactions were computed via the Particle Mesh Ewald (PME) method with a 1.2 nm cutoff.

### Correlation analysis of core targets with clinical characteristics and prognosis of CRC

Gene expression and survival analysis were conducted using the public databases UALCAN [17] and GEPIA [18], respectively. In UALCAN, six candidate target gene names were entered and the corresponding cancer type was specified. The expression data of each gene in normal tissues (Normal) and cancer tissues, different stages of cancer (Normal, Stage Ⅰ-Ⅳ), and different lymph node metastasis statuses (Normal, N0, N1, N2) were extracted, with expression quantified as transcripts per million (TPM). In GEPIA, the same cancer types and candidate genes were analyzed using “Single Gene Analysis” module to generate overall survival (OS) and disease-free survival (DFS) curves. The Log-rank test was applied to calculate *P* value and hazard ratio (HR).

### Cell culture and general reagents

HCT116 and RKO human colorectal cancer cells were procured from the Shanghai Cell Bank, Type Culture Collection Committee of Chinese Academy of Science (Shanghai, China). They were cultured in RPMI1640 supplemented with 10% FBS and 1% penicillin/streptomycin at 37 °C in a 5% CO_2_ incubator. Quercetin (MCE, HY-18085) and kaempferol (MCE, HY-14590) were used in vitro.

### Cell viability assay

The cell counting kit-8 (Yeasen) was utilized to assess cell viability. Cells were seeded at 8 × 10^3^ cells per well in 96-well plates. Following cell adhesion, the cells were treated quercetin and kaempferol for either 24 or 48 h. Subsequently, 10 μL of CCK-8 solution was added and incubated for an additional 2 h at 37 °C. Cell viability was quantified by measuring the optical density.

### Analysis of drug combination effects

The drug's inhibitory rate at different concentrations was measured through CCK-8 experiments. Dose–response curves and combination index curves were plotted using CompuSyn software. Further visual analysis was conducted using SynergyFinder software [13].

### Flow cytometry analysis

For cell cycle analysis, cells underwent harvesting, washing, and fixation in ice-cold 70% ethanol overnight. Subsequently, they were resuspended in 250 μl PBS with 5 μl RNase A (10 mg/ml) and incubated for 1 h. Afterward, 10 μl of PI solution (10 μg/ml) was added to the samples, which were then incubated in the dark at room temperature for 10 min before being analyzed by CytoFLEX flow cytometry (Beckman Coulter).

For apoptosis analysis, cells were assessed for apoptosis using the Alexa Fluor 647 Annexin V/PI Kit (Yeasen). They were washed with cold PBS, detached with 0.25% trypsin, and centrifuged at 600 g for 3 min. After removing the supernatant, cells were resuspended in binding buffer at 1 × 10^6^ cells/ml. A 100 µl cell suspension was mixed with 5 µl Annexin V-Alexa Fluor 647 and 10 µl PI, incubated for 15 min at room temperature, and analyzed by CytoFLEX flow cytometry within an hour.

### Transwell assays

For the migration assay, 5 × 10^4^ cells were seeded into the upper compartments of transwell filters (Labselect). Following a 24-h incubation period, the cells were fixed using 4% paraformaldehyde and subsequently stained with a 1% crystal violet solution. The migrated cells adhering to the underside of the filter were then photographed, and quantification was performed by counting cells in three randomly selected microscopic fields per well, with the mean value calculated. Invasion assays were performed similarly after coating the inserts with a diluted matrigel matrix.

### Wound healing assay

Scratch wound assays were performed by quantifying the scratch area at various time intervals. A total of 2 × 10^5^ cells were seeded into each well of 6-well plates. Following a 48-h incubation period, a linear scratch was introduced to the bottom of the wells using a 200 μL pipette tip. Images of the identical regions were captured at designated time points (0, 24, and 48 h) post-injury.

### Western blot

The cells were lysed utilizing RIPA buffer (Beyotime) supplemented with InStab™ Protease Inhibitor Cocktail (Yeasen). The resulting lysates were subjected to denaturation at 100 °C for a duration of 10 min. Subsequently, 20 μg of total protein was resolved on an SDS-PAGE gel, transferred onto PVDF membranes (Millipore, MA, USA), blocked with Protein Free Rapid Blocking Buffer (EpiZyme) and incubated with primary antibodies overnight at 4 °C followed by secondary antibodies at room temperature. GAPDH served as an internal loading control.

### Statistical analysis

GraphPad Prism software (v10.3.0) was used for statistical analysis. All results were presented as mean ± standard deviation (mean ± SD), and independent t-tests were used to compare significant differences between two groups. A *P* value < 0.05 was considered significant statistically.

## Results

### Component characteristics and prediction of TCM combinations

**Fig.2:**
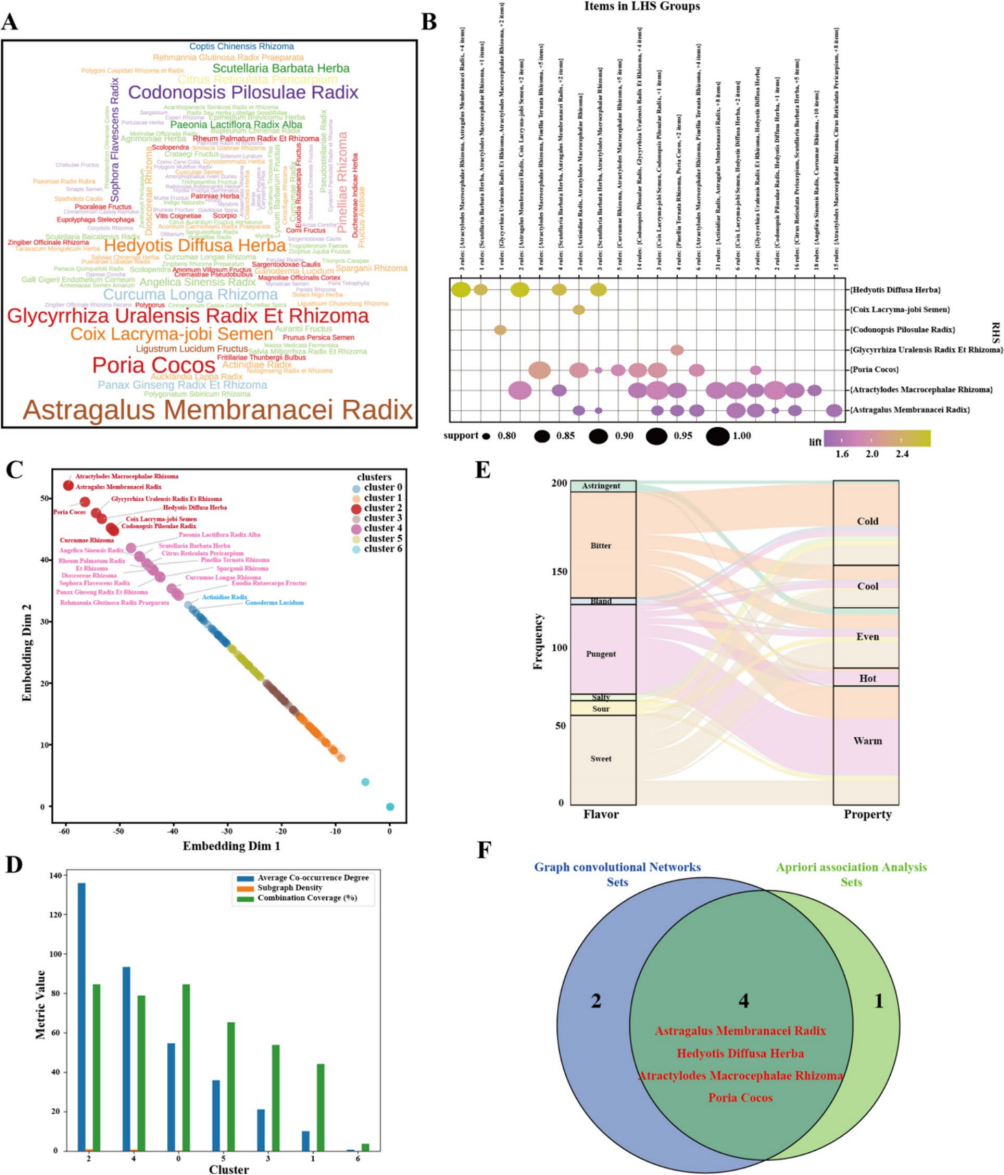
Analysis of the composition, properties, and compatibility rules of TCM for preventing and treating colorectal cancer recurrence. **A** Word cloud of herbs. **B** Grouping matrix for the 148 association rules. **C** Clustering visualization distribution of TCM herb nodes based on graph embedding. **D** Metric value of different TCM herb clusters. **E** Distribution of Herbal Properties (Nature and Flavor). **F** Venn diagram of core herb identified by GCN and Apriori association rule

In this study, we screened and collected 102 TCM prescriptions (STable.1) for the prevention and treatment of CRC recurrence from RCTs associated TCM combinations, marketed Chinese patent drug and TCM combinations obtained a patent. These prescriptions were composed of multiple herbal combinations, and after summarization and duplication, a total of 216 different herbs were finally included (Fig. 2A).

To explore the compatibility rules of herbs in the prescriptions, we analyzed 102 prescriptions using the Apriori association rule, and a total of 148 valid association rules (STable.2) were identified. The compatibility relationships between the left-hand side (LHS) and right-hand side (RHS) herb item sets in the association rules can be clearly presented through a matrix diagram (Fig. 2B). With lift as the sorting criterion, the association rule “*Atractylodes Macrocephalae Rhizoma, Astragalus Membranacei Radix, Poria Cocos, Coix Lacryma-jobi Semen*, *Hedyotis Diffusa Herba*” showed the highest lift. Under this rule, the appearance probability of *Hedyotis Diffusa Herba* reached 92.31% (confidence = 0.9231) when the combination of *Atractylodes Macrocephalae Rhizoma, Astragalus Membranacei Radix, Poria Cocos* and *Coix Lacryma-jobi Semen* was present in a prescription. Meanwhile, compared with the probability of using *Hedyotis Diffusa Herba* alone, this combination could increase the likelihood of using *Hedyotis Diffusa Herba* by 2.82 times.

In order to more accurately explore the intrinsic relationship between composition and efficacy of prescriptions, we employed a two-layer GCN to perform embedding learning on the herbs. The herbs within the same cluster exhibit a high degree of similarity in terms of their characteristics and efficacy directions. Among them, we observed that eight herbs, including *Atractylodes Macrocephalae Rhizoma, Astragalus Membranacei Radix, Poria Cocos, Glycyrrhiza Uralensis Radix Et Rhizoma, Coix Lacryma-jobi Semen, Hedyotis Diffusa Herba, Curcumae Rhizoma* and *Codonopsis Pilosulae Radix*, were classified into cluster 2 (Fig. 2C). These herbs have the highest average co-occurrence degree and relatively higher combination coverage (Fig. 2D). The GCN results suggest that strengthening the spleen, clearing heat, drying dampness and eliminating blood stasis are the classic compound prescription logic for preventing and treating the recurrence of colorectal cancer. Meanwhile, an analysis of the properties of these herbs in terms of nature and flavor reveals that most are astringent and bitter (Fig. 2E). From the perspective of medicinal properties, these herbs mostly correspond to cold or cool qualities. Based on TCM theory, it can be further summarized that strengthening the spleen, clearing heat, resolving blood stasis, and drying dampness are the core functions, which are in line with the needs of pathogenesis for CRC recurrence.

GCN analysis and association rule mining collectively identified a core herb spectrum associated with anti-CRC recurrence prescriptions, including *Atractylodes Macrocephalae Rhizoma, Astragalus Membranacei Radix, Poria Cocos, Glycyrrhiza Uralensis Radix Et Rhizoma, Coix Lacryma-jobi Semen, Hedyotis Diffusa Herba, Curcumae Rhizoma* and *Codonopsis Pilosulae Radix*. Among these, the overlapping herbs identified by both methods were prioritized for subsequent mechanistic and experimental analyses (Fig. 2F).

### Validation of potential core herbs

To validate the reliability of core herbs, general treatment prescriptions for CRC were systematically reviewed using evidence-based medicine principles and compared with anti-recurrence prescriptions. A total of 85 general treatment prescriptions were analyzed, comprising 198 herbs (STable.3). *Astragalus Membranacei Radix*, *Hedyotis Diffusa Herba*, *Atractylodes Macrocephalae Rhizoma*, *Coix Lacryma-jobi Semen*, *Curcumae Rhizoma*, *Poria Cocos*, and *Codonopsis Pilosulae Radix* were frequently found in anti-recurrence prescriptions, while *Scutellaria Barbata Herba*, Actinidiae Radix and *Sophora Flavescens Radix* showed moderate enrichment, indicating distinct compositional variations between the two groups.

Given the high-dimensional and sparse nature of TCM formulas, containing interrelated components, and the stable modeling capability of random forests without assuming a specific data distribution, a random forest model was developed. Results indicated varying contributions of different herbs in distinguishing between the anti-recurrence and non-anti-recurrence groups, with an OOB error rate of 25.53%. This suggests distinct roles of individual herbs in the efficacy of the prescriptions. According to the mean decrease accuracy ranking, *Hedyotis Diffusa Herba*, *Astragalus Membranacei Radix*, *Curcumae Rhizoma*, *Coix Lacryma-jobi Semen*, and *Atractylodes Macrocephalae Rhizoma* were among the top five, highlighting their importance in model discrimination (STable.4). *Hedyotis Diffusa Herba* received the highest importance score, indicating its significant impact on model performance. *Codonopsis Pilosulae Radix* and *Poria Cocos* were also ranked in the top 14, suggesting their auxiliary roles in the formulations. The findings of the random forest model were highly consistent with those of the Apriori association rule and GCN models, supporting further exploration of the potential core herbs.

### Enrichment analysis of combined herb target genes and pathways

To clarify the potential targets of this herbal combination, we collected the known target genes related to CRC and the genes targeted by the compatible herbs through the TCMSP database. To deeply understand the functions and biological significance of these target genes, we performed GO and KEGG pathway enrichment analysis.

**Fig.3:**
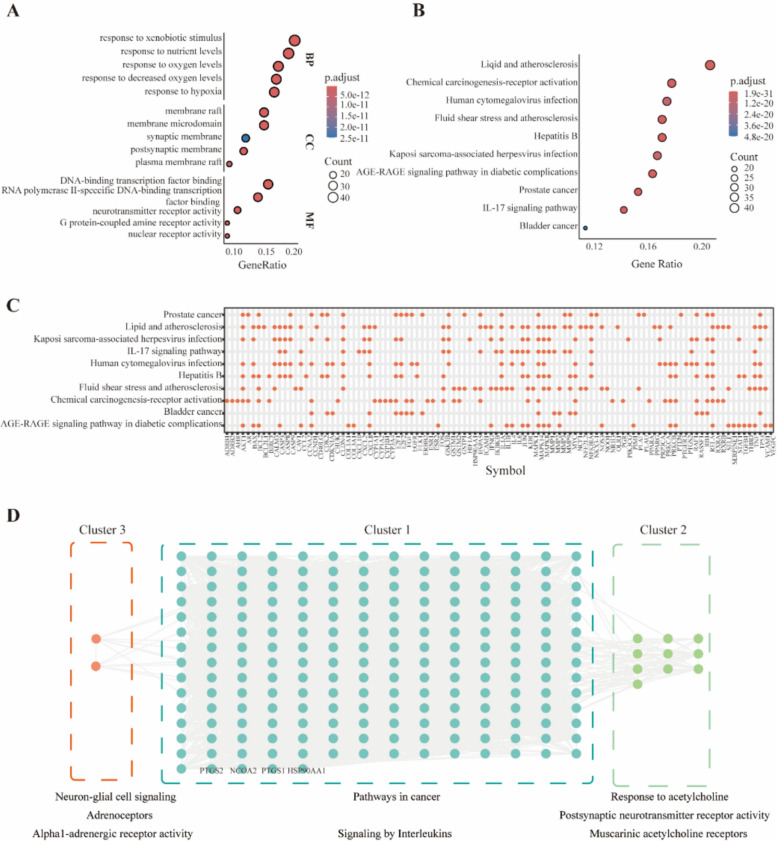
Enrichment analysis of combined herb target genes.** A** GO enrichment analysis of target genes. **B** KEGG enrichment analysis of target genes. **C** Enrichment of target genes in multiple signaling pathways. **D** Cluster analysis of target genes and biological pathways

GO functional enrichment analysis reveals that target genes have significant roles in Biological Process (BP), Cellular Component (CC), and Molecular Function (MF) (Fig. 3A). In BP, they are involved in response to external stimuli and oxidative stress, linked to tumor microenvironment and metabolic reprogramming. In CC, they are enriched in lipid rafts, synaptic membranes and postsynaptic membranes, indicating roles in cell signaling and transport. In MF, they participate in transcription factor binding and nuclear receptor activity, emphasizing their importance in gene expression and signaling. These insights are crucial for understanding target genes’ roles in colorectal cancer development.

KEGG pathway enrichment analysis indicated that the screened potential target genes were significantly enriched in pathways including lipid and atherosclerosis, chemical carcinogenesis-receptor activation, prostate cancer, IL17 signaling pathway and bladder cancer (Fig. 3B). To visually present the pathway enrichment characteristics of target genes, we used a point graph to visualize the enrichment of target genes in multiple signaling pathways of this herbal combination (Fig. 3C). The results showed that multiple target genes were concentrated in pathways closely related to tumor occurrence and development, inflammatory response, and metabolic disorders, further confirming that this herbal combination may exert anti-CRC recurrence effects by regulating these pathways.

In order to clarify the specific association between target genes and biological pathways, we conducted cluster analysis on target genes. The results showed that all target genes were ultimately divided into three categories (Fig. 3D). The target genes in Cluster 1 are involved in pathways related to cancer and signaling by interleukins. The key genes include *PTGS2*, *NCOA2*, *PTGS1*, *GABRA1* and *HSP90AA1*, which play a central role in the occurrence and development of cancer and inflammatory response [19–21]. Cluster 2 and Cluster 3 represent two major functional modules of adrenergic neural signaling and cholinergic neural signaling, respectively. These reflect distinct branches of the neural signal transduction system.

### Screening of active ingredients and targets in combined herbs

**Fig.4:**
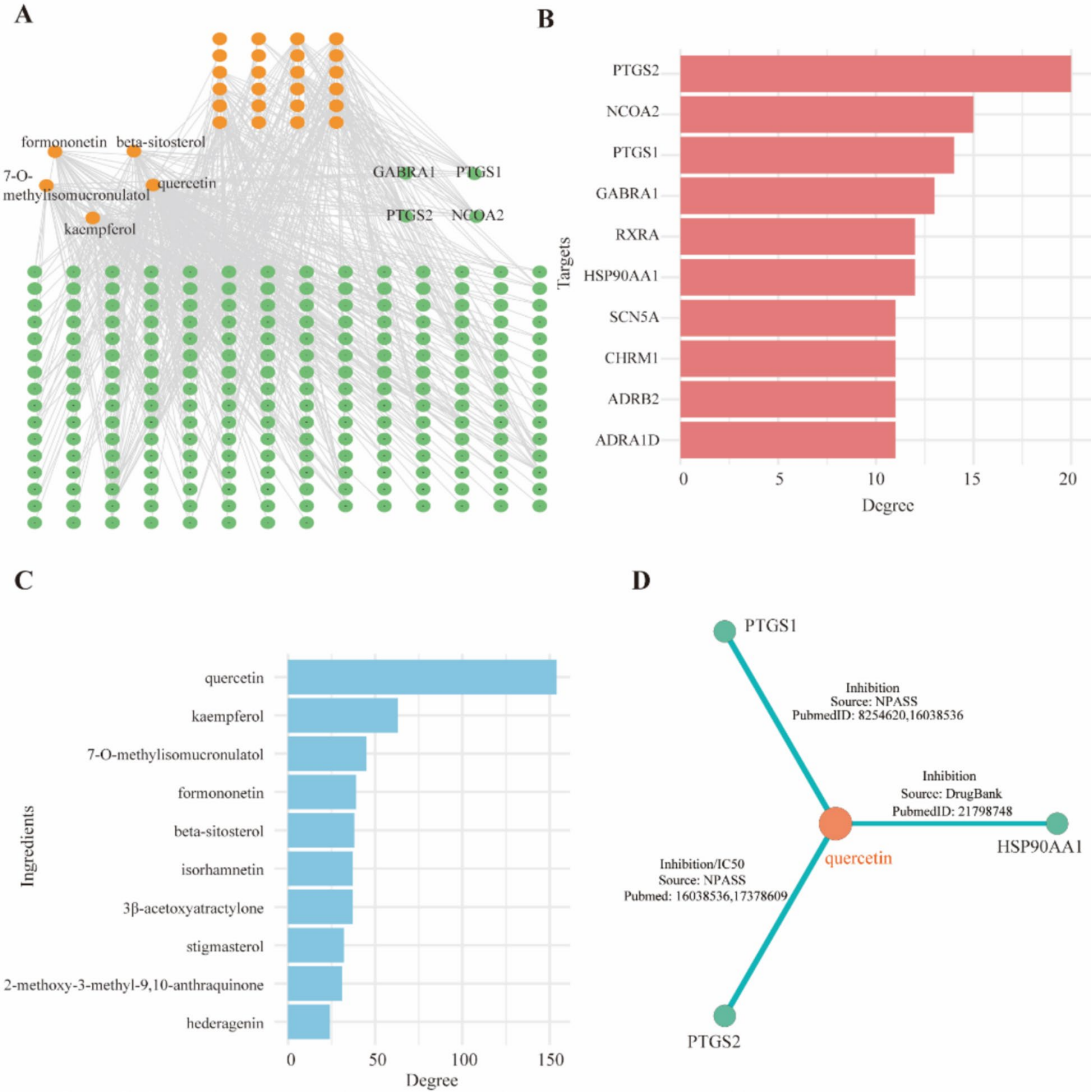
The compound-protein network diagram. **A** Interaction between active ingredients and target proteins. **B** The top ten active ingredients in compatible TCM ranked by degree. **C** The top ten target proteins in compatible TCM ranked by degree. **D** Quercetin can act on three targets, PTGS1, PTGS2, and HSP90AA1, simultaneously

To further identify the key active components and core targets of the compatibility herbal medicine including *Astragalus Membranacei Radix*, *Hedyotis Diffusa Herba*, *Atractylodes Macrocephalae Rhizom* and *Poria Cocos*, we constructed a component-target interaction network based on the TCMSP database (Fig. 4A). In this network, orange nodes represent the active components identified from herbs, green nodes represent potential target proteins, and the edges connecting the nodes represent the interactions between them. The key targets commonly affected include PTGS2, NCOA2, PTGS1, GABRA1, RXRA, HSP90AA1, SCN5A, CHRM1, ADRB2 and ADRAID (Fig. 4B).

Through statistical analysis of the connectivity (degree) of network nodes, the core active components with top-ranked degree values were selected out, including quercetin, kaempferol, 7-O-methylisomucronulatol, formononetin, beta-sitosterol, isorhamnetin, 3β-acetoxyatractylone, stigmasterol, 2-methoxy-3-methyl-9,10-anthraquinone and hederagenin (Fig. 4C). To further refine the screening results, we also conducted a screening of key ingredients and core targets in HIT, ITCM, and TCMBank databases. We summarized the top five active ingredients (SFig.1A) and core targets (SFig.1B). This result not only verifies the accuracy of the predictions made by the TCMSP database, but also provides a clear direction for subsequent verification. Combined with previous research reports, these components have definite activities in inhibiting tumor cell proliferation, regulating inflammatory responses, and interfering with tumor metabolism, which suggests that they may be the key material basis for this compatible herbal combination to antagonize the development of CRC [22–24]. Meanwhile, utilizing the ccTCM database, we extracted literature-supported interactions involving the specified compounds and their respective targets, with a focus on quercetin. The interactions identified include two instances of inhibition between quercetin and PTGS1, two instances of both IC_50_ and inhibition between quercetin and PTGS2, as well as documented activity between quercetin and HSP90AA1. These findings offer a foundational dataset for subsequent analyses of quercetin's mechanism of action. (Fig. 4D). In addition, the molecular docking results showed that both quercetin and kaempferol had strong binding affinity with all six target proteins. Quercetin, in particular, demonstrated the lowest binding energy with PTGS2 at −9.25 kcal/mol, suggesting the highest binding affinity (SFig.2A and STable.5). Similar to the docking results with molecules, molecular dynamics results demonstrate stable interactions between quercetin and kaempferol with the target protein (SFig.2B and STable.6).

### Correlation analysis of core targets with clinical characteristics and prognosis of CRC

Based on the core target genes (*PTGS2, NCOA2, PTGS1, HSP90AA1*) identified through previous screening, this study used the UALCAN and GEPIA database platforms to conduct expression and survival analyses.

**Fig.5:**
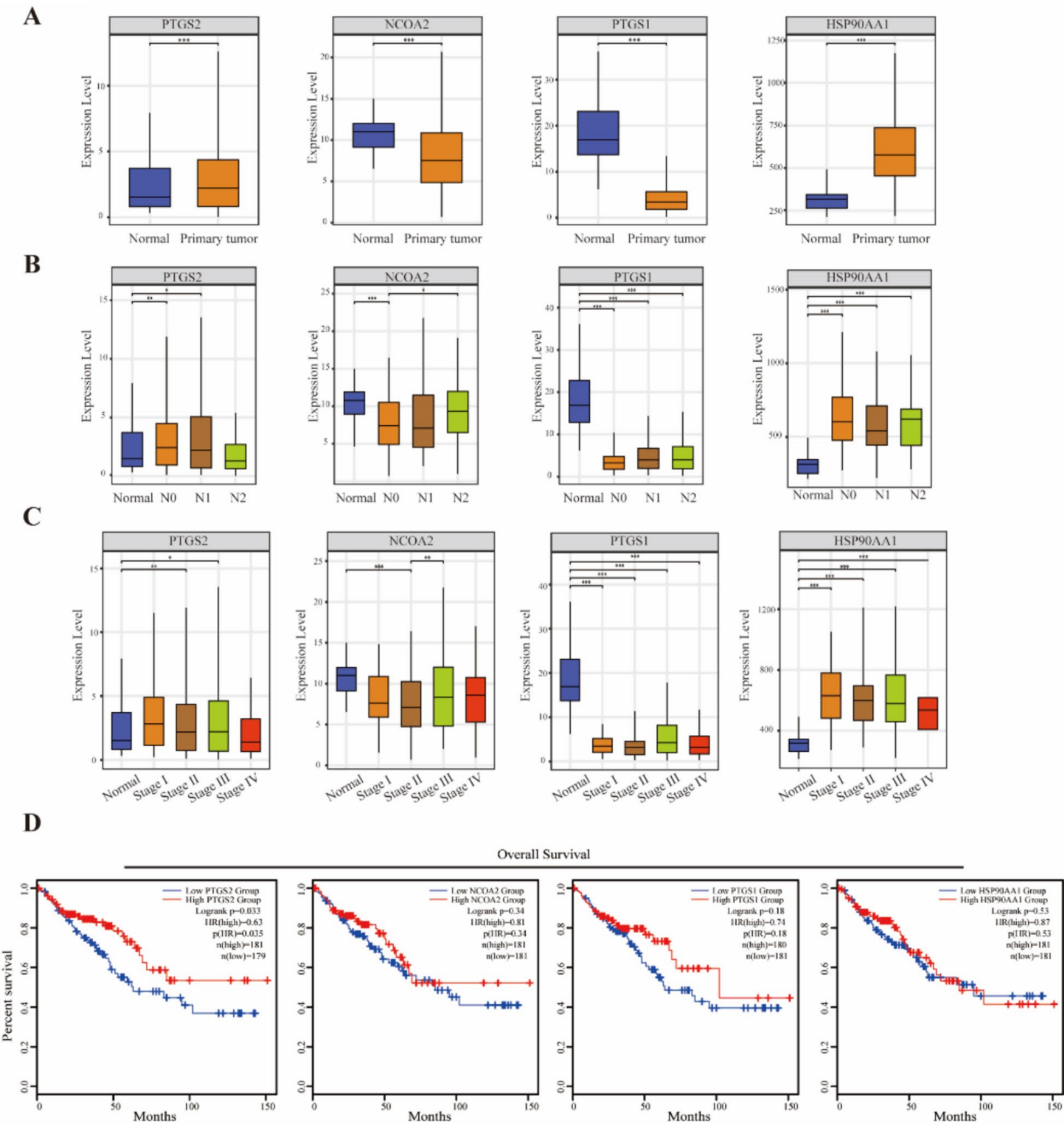
Correlation analysis of core targets with clinical characteristics and prognosis of CRC. **A** Expression differences of target genes in normal and primary tumor tissues. **B** Expression differences of target genes in N staging. **C** The relative expression levels of target genes in various pathological stages of CRC. **D** Survival analysis of target genes with different expression levels

In the UALCAN database, taking CRC as the research subject, we obtained the TPM expression values of the above 4 target genes under different tissue statuses. Comparison between normal tissues and cancer tissues showed that the expressions of *PTGS2* and *HSP90AA1* were significantly upregulated in cancer tissues (*P*<0.001), while those of *NCOA2* and *PTGS1* were significantly downregulated (*P*<0.001) (Fig.5A). Stratified analysis of lymph node metastasis status (Normal, N0, N1, N2) showed that the 4 target genes exhibited the most significant expression differences between the Normal group and the N0 stage (*P*<0.01), while there was no statistically significant change in the expression changes between the N1 and N2 stages (Fig.5B). Further analysis of the expression differences across different stages (Normal, Stage Ⅰ-Ⅳ) revealed that the mentioned genes showed statistical differences between the normal stage and each pathological stage, and their expression trends remained stable with the progression of stages (*P*<0.01) (Fig.5C). Combined with the clinical features that the N0 stage correspond to stage I and II colon cancers, this suggests that these genes may serve as potential biomarkers for early diagnosis. Survival analysis in the GEPIA database showed that *PTGS2* was the only target whose expression was significantly associated with prognosis, with patients in the high *PTGS2* expression group showing significantly better OS than those in the low expression group (HR=0.63, Log-rank *P*<0.05), while DFS showed a similar trend (Fig.5D). The survival curves of the remaining 3 target genes showed no statistical difference (*P*>0.05). These results suggest that *PTGS2* is not only a core target of the herb compatibility, but its expression level can also serve as a reference indicator for evaluating tumor progression and recurrence prognosis in CRC patients.

### Quercetin and kaempferol induce apoptosis and arrest cell cycle in CRC cells

**Fig.6:**
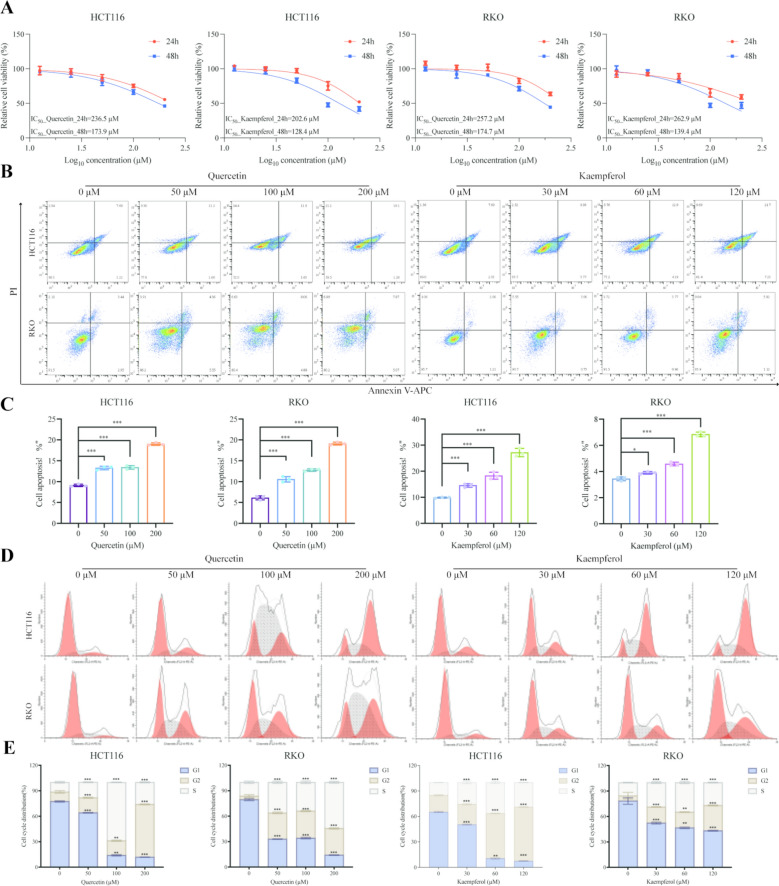
Cell viability assay and flow cytometry analysis of HCT116 and RKO cells. **A** Cell viability of HCT116, RKO cells after incubation with various concentrations of quercetin and kaempferol for 24 and 48 h. **B, C** The representative images and statistical results of cell apoptosis in HCT116 and RKO cells. **D, E** The determination of cell cycle of HCT116 and RKO cells by using flow cytometry

To verify the anti-tumor activity of quercetin and kaempferol, we performed CCK-8 assay in the colorectal cancer cell lines HCT116 and RKO. Quercetin and kaempferol effectively inhibit the growth of HCT116 and RKO cells in vitro in a dose- and time-dependent manner. After intervention with quercetin, the 48 h half-maximal inhibitory concentration (IC_50_) of HCT116 and RKO cells were 173.9 μM and 174.7 μM, respectively; while the IC_50_ of HCT116 and RKO cells treated with kaempferol were 128.4 μM and 139.4 μM, respectively (Fig. 6A). Meanwhile, flow cytometry results showed that as the concentrations of quercetin and kaempferol gradually increased, the fluorescent signals of cell apoptosis increased significantly in a dose-dependent manner (Fig. 6B, C). To further evaluate whether the anti-tumor effects of quercetin and kaempferol would induce cell cycle arrest, cell cycle analysis results revealed that the proportion of cells in G2 phase increased with increasing drug concentration, after treating HCT116 and RKO cells with drugs (Fig. 6D, E).

### Quercetin and kaempferol inhibits the migration and invasion of CRC cells

**Fig.7:**
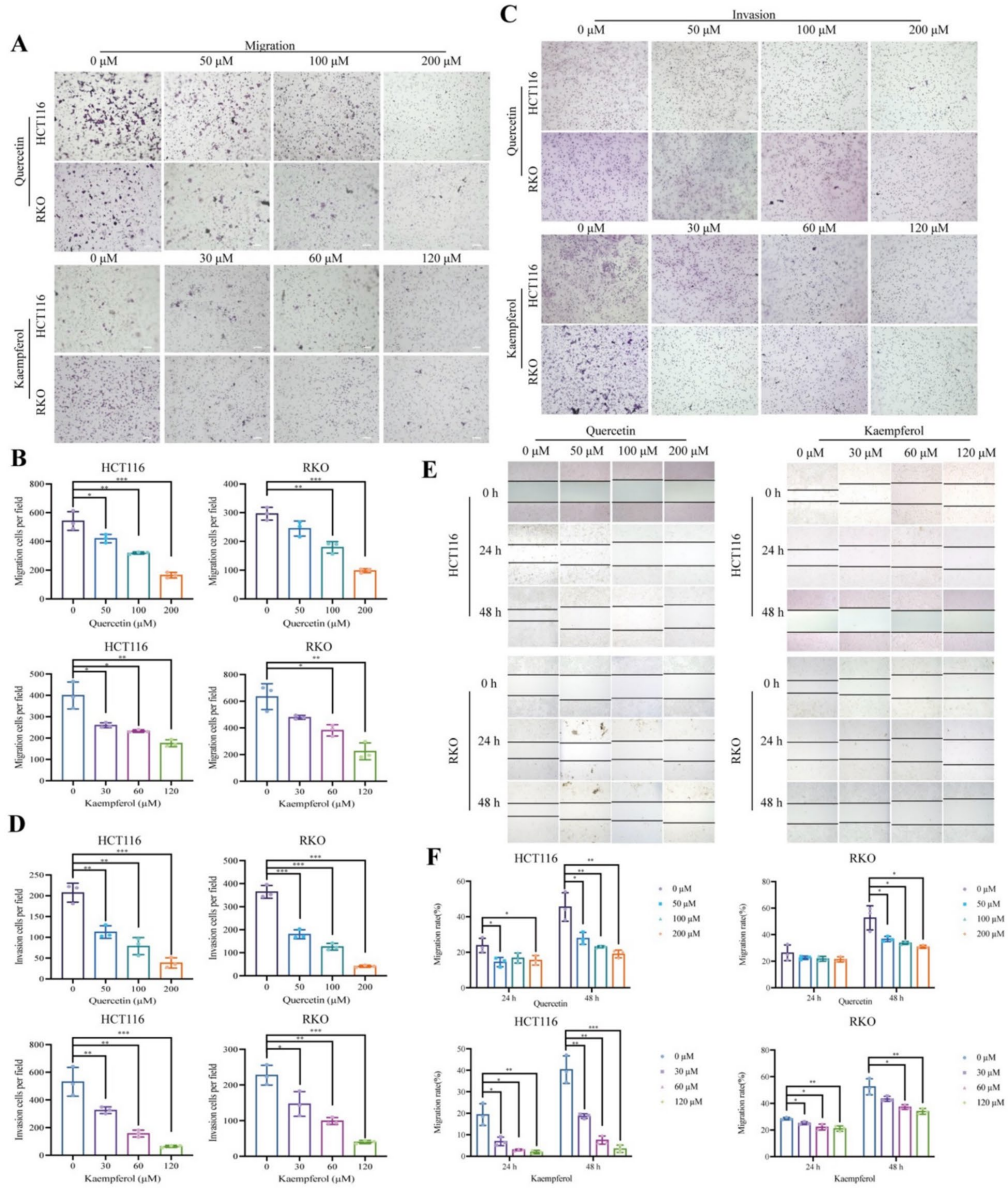
Transwell assay and wound healing assay of HCT116 and RKO cells. **A, B** Representative images and cell count of migration assays for HCT116 and RKO cells. **C, D** Representative images and cell count of invasion assays for HCT116 and RKO cells. **E** Wound-healing experiments of HCT116 and RKO cells for 24 h and 48 h. **F** Cell migration rates were plotted by respective histogram

To determine whether migration and metastasis are involved in the anti-tumor effects of quercetin and kaempferol, we performed transwell assays and wound healing assays. In the transwell assay, we observed that compared to the control group, quercetin and kaempferol reduced the number of migrated HCT116 and RKO cells (Fig. 7A, B). Additionally, the cell invasion assay showed that the two drugs inhibited the invasiveness of CRC cells (Fig. 7C, D). In wound healing assay, HCT116 and RKO cells were incubated with different concentrations of the drugs for 0, 24, and 48 h, respectively. The results showed that compared with the control group, both quercetin and kaempferol significantly slowed down wound healing (Fig. 7E, F). These results confirm that quercetin and kaempferol can significantly inhibit the invasion and metastasis abilities of CRC cells in vitro.

### Quercetin and kaempferol inhibit the expression of PTGS2

**Fig.8:**
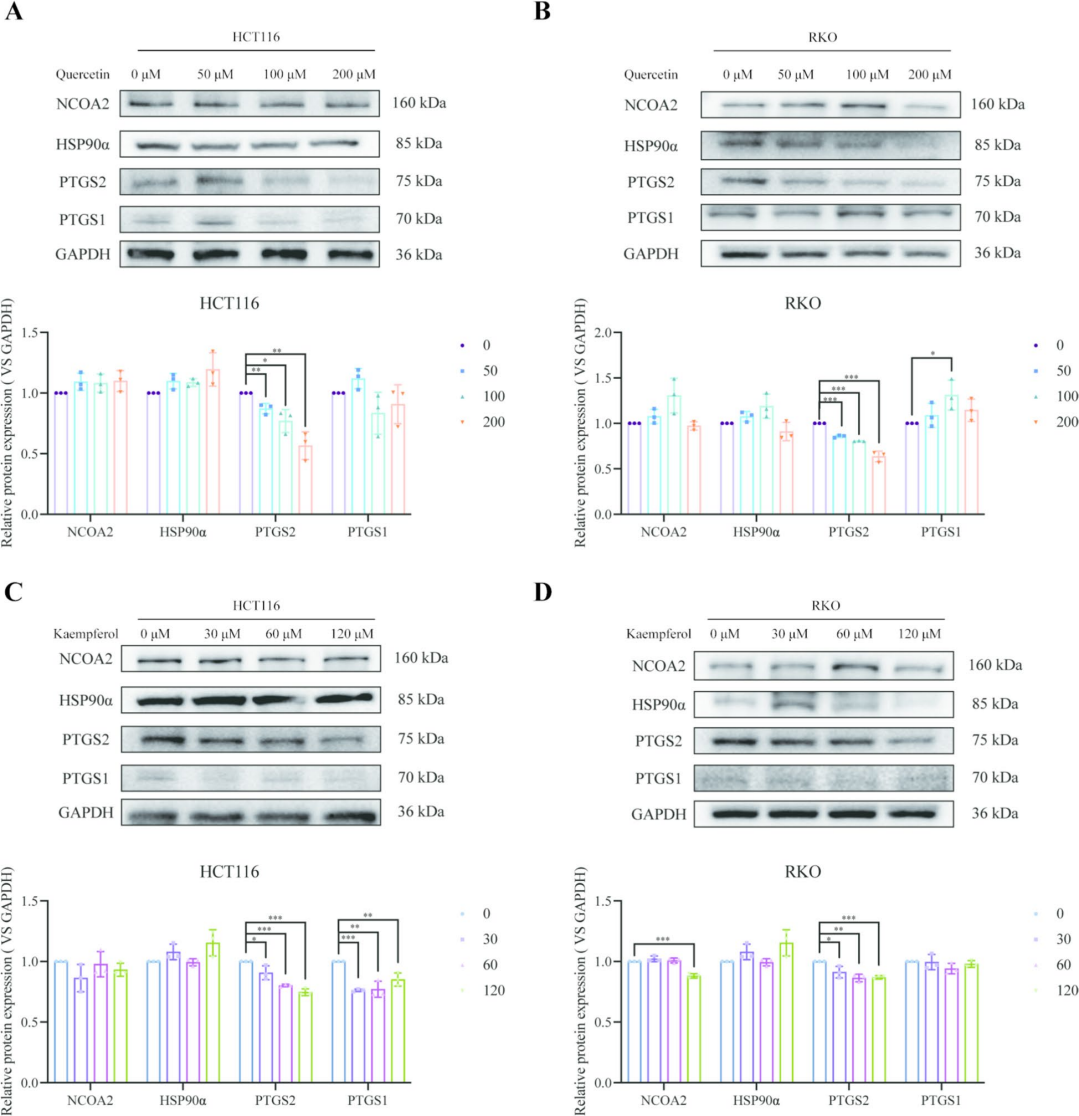
Expression of target genes in HCT116 and RKO cells. **A** The protein levels of target genes in HCT116 cells treated with quercetin. **B** The protein levels of target genes in RKO cells treated with quercetin. **C** The protein levels of target genes in HCT116 cells treated with kaempferol.** D** The protein levels of target genes in RKO cells treated with kaempferol

Based on the analysis results from public databases and the analysis of the potential target effects of drugs, it is inferred that PTGS2, PTGS1, NCOA2, HSP90AA1, PRSS1 and GABRA1 are potential molecular targets of quercetin and kaempferol in the TCM prescription for inhibiting colorectal recurrence. Through western blot analysis, we further investigated the relationship between quercetin, kaempferol and target proteins. The results showed that quercetin and kaempferol exerted a significant inhibitory effect on PTGS2, making them effective candidate drugs for the treatment of CRC recurrence (Fig. 8A-D). Lastly, considering that the Chinese medicines from which the two compounds are derived are often used in combination in prescriptions, we wondered whether they exhibit a synergistic effect. Therefore, we conducted drug combination experiments, and observed that quercetin and kaempferol had a certain potentiating effect. However, the mean of ZIP synergy scores was consistently greater than 1 at different concentrations, indicating that there was no significant drug synergy between the two (SFigure.3).

## Discussion

This study identified candidate herbal combinations for preventing CRC recurrence based on TCM prescriptions. Subsequent compound-target analysis and experimental validation were conducted to substantiate the potential advantages of these prescription-derived combinations. Through screening 102 TCM prescriptions for efficacy in preventing or treating CRC recurrence, along with 86 general treatment prescriptions, a core herb combination was identified. This combination consisting of *Astragalus Membranacei Radix, Hedyotis Diffusa Herba, Atractylodes Macrocephalae Rhizoma* and *Poria Coco* was determined using association rule mining and GCN analysis. Differential analysis between anti-recurrence and general treatment prescriptions further supported this core combination. Network pharmacology analysis highlighted that quercetin and kaempferol as potential principal active components, with PTGS2 identified as a central target across various databases. Molecular docking and dynamics simulations confirmed a stable interaction between quercetin and PTGS2. Clinical relevance of these targets, particularly *PTGS2*, in CRC development and prognosis was indicated through UALCAN and GEPIA analyses. In vitro experiments demonstrated that quercetin and kaempferol inhibited CRC cell proliferation, migration, and invasion, induced apoptosis, and suppressed PTGS2 expression. These findings collectively support the rationality and anti-recurrence potential of the identified core herbal combination, providing a scientific foundation for its further development in CRC recurrence prevention.

The study reveals a consistent core herb spectrum in anti-CRC recurrence prescriptions, identified through GCN analysis and Apriori association rule mining. Key herbs include *Atractylodes Macrocephalae Rhizoma, Astragalus Membranacei Radix, Poria Cocos, Glycyrrhiza Uralensis Radix Et Rhizoma, Coix Lacryma-jobi Semen, Hedyotis Diffusa Herba, Curcumae Rhizoma* and *Codonopsis Pilosulae Radix*. These herbs collectively target spleen-qi, clearing heat, resolving dampness, promoting blood circulation, and eliminating pathogenic factors, aligning with TCM’s view of CRC recurrence. Pharmacological studies support this prescription, showing immunomodulatory and anti-tumor properties in these herbs. For instance, *Astragalus Membranacei Radix* and *Curcumae Rhizoma* not only enhances sorafenib efficacy against hepatocellular carcinoma (HCC) and regulate glycolysis to suppress CRC metastasis [25, 26]. *Atractylodes Macrocephalae Rhizoma* can exert immunomodulatory effects by activating macrophages through pathways such as NF-κB and JAK-STAT [27]. Lobetyolin, an active component of *Codonopsis Pilosulae Radix*, induces HCT116 cell apoptosis by inhibiting ASCT2-mediated glutamine metabolism, potentially regulated by p53 [28, 29].

Quercetin, a natural polyphenolic flavonoid compound, exhibits anti-inflammatory, antioxidant, anti-tumor, and immunomodulatory properties [30, 31]. Previous studies have shown that its inhibitory effects on various solid tumor cells, such as HCC, breast cancer, CRC, and pancreatic cancer [31, 32]. In CRC, quercetin can regulate the tumor immune microenvironment, enhance T-cell infiltration and activation, and ultimately suppress tumor recurrence by promoting anti-tumor immune memory [33]. Moreover, it has been reported to inhibit angiogenesis and induce tumor cell apoptosis through pathways such as NFAT, Wnt3a/β-catenin, and Akt/NF-κB [34, 35]. Another natural flavonoid, kaempferol also possesses anti-tumor and immunoregulatory properties. Previous studies have shown that kaempferol can inhibit CRC progression by inducing pyroptosis, suppressing metastasis, and alleviating chemotherapy resistance [36]. Our findings align with these results, highlighting quercetin and kaempferol as key active compounds that effectively inhibit malignant behaviors in CRC cells.

PTGS2 (also known as COX2) plays a crucial role in prostaglandin synthesis and serves as a significant mediator connecting inflammation to tumor progression. Its overexpression has been reported in multiple malignancies, including lung cancer, breast cancer and CRC [37–39]. Prior research has demonstrated that PTGS2 facilitates tumor advancement by shaping a pro-metastatic microenvironment, while its inhibition can suppress metastasis [40]. In our investigation, quercetin and kaempferol emerged as the principal active compounds in the prioritized herbal combination, with implications pointing to their potential modulation through PTGS2. Combination experiments revealed an enhanced inhibitory trend without a significant synergistic effect, likely due to the structural and mechanistic similarities between the two compounds [40]. Both flavonoids have overlapping tumor-related targets, such as AKT1, TP53, and STAT3, and regulate core pathways like PI3K/AKT, MAPK, and mTOR [41–44]. The combined use of quercetin and kaempferol may primarily reinforce existing regulatory effects on common targets and pathways rather than introduce new functional effects. Optimal drug synergy often relies on specific dose ratios and exposure conditions, which may not have been achieved in this study. Despite the absence of marked synergy, the combined administration of quercetin and kaempferol exemplifies the multi-component, multi-target, and holistic characteristics of TCM-based interventions. This combination may still confer broader anti-tumor effects by collectively impacting tumor proliferation, metastasis, and the tumor microenvironment [45].

Alongside the host target-based mechanisms identified by network pharmacology, it is important to also consider the potential interaction between TCM and gut microbiota. In CRC, gut microbial dysbiosis is closely associated with intestinal inflammation, immune imbalance, and tumor progression [46]. Recent studies suggest that the anti-tumor effects of TCM may involve modulation of gut microbiota composition, microbial metabolites, intestinal barrier function, and immune responses [47, 48]. Therefore, the anti-recurrence effects of the core herbal prescription identified in the present study may not be solely explained by compound–target interactions. This may be particularly relevant to flavonoids such as quercetin and kaempferol, as gut microbiota can participate in flavonoid metabolism and biological effects, thereby influencing their pharmacokinetic behavior as well as downstream pharmacodynamic activities related to intestinal inflammation [49] and barrier regulation. For quercetin, evidence suggests that it can modulate gut microbiota-related metabolism by increasing the abundance of *Clostridium_XIVa* and *Clostridium_XI*, thereby promoting the microbiota-dependent production of isovanillic acid, a microbial metabolite involved in intestinal barrier protection and inflammatory regulation [50]. Quercetin has also been reported to regulate gut microbiota–bile acid crosstalk [51]. Similarly, in ApcMin/+ mice, kaempferol was shown to reshape gut microbiota and regulate bile acid metabolism, accompanied by reduced intestinal tumor burden and improved barrier function [52]. These findings suggest that gut microbiota may represent an additional mechanistic layer linking the identified herbal combination and its representative compounds with CRC recurrence-related microenvironmental regulation. While not directly studied here, this aspect suggests a valuable direction for future research. Comprehensive analyses integrating gut microbiota, metabolomics, and host signaling could enhance understanding of the holistic mechanisms underlying TCM-based recurrence prevention.

Despite identifying a core herbal combination, representative active components, and key targets related to CRC recurrence prevention, this study still has some limitations. Firstly, the current evidence is primarily derived from in vitro experiments and lacks validation in vivo mouse models, particularly CRC recurrence models. Further studies incorporating PTGS2 knockout or knockdown alongside drug treatment may help clarify whether the observed effects are PTGS2-dependent. Secondly, the interaction pattern of quercetin and kaempferol requires further elucidation. Finally, the possible contribution of gut microbiota was not directly addressed in the present study.

## Conclusion

By integrating Apriori association rule mining and GCN analysis, this study identified a core herbal combination related to CRC recurrence prevention and prioritized a high-confidence herbal combination for mechanistic investigation. Subsequent network pharmacology analysis highlighted quercetin and kaempferol as active compounds and PTGS2 as a potential key target, findings further supported by in vitro experiments. These results provide evidence for the rationality and anti-recurrence potential of prescription-derived herbal combinations, offering a scientific basis for the further application of TCM in CRC recurrence prevention.

## Supplementary Information

Supplementary Material 1

Supplementary Material 2

Supplementary Material 3

## Data Availability

The datasets used or analyzed among the current study will be made available from the corresponding author on reasonable request.

## Abbreviations

BP: Biological Process
CC: Cellular Component
ChiCTR: Chinese Clinical Trial Registry
CIDs: Compound Identifiers
CRC: Colorectal cancer
DFS: Disease-free Survival
DL: Drug-likeness
Fdr: False discovery rate
GCN: Graph convolutional network
GO: Gene ontology
HCC: Hepatocellular carcinoma
HIT: Herb Ingredients' Targets
HR: Hazard ratio
IC_50_: Half-maximal inhibitory concentration
ITCM: Integrated Traditional Chinese Medicine
ITMCTR: International Traditional Medicine Clinical Trial Registry
KEGG: Kyoto encyclopedia of genes and genomes
LHS: Left-hand side
MF: Molecular Function
OB: Oral bioavailability
OOB: Out-of-bag
OS: Overall survival
PME: Particle Mesh Ewald
PPI: Protein–protein Interaction
RCTs: Randomized controlled trials
RHS: Right-hand side
SD: Standard deviation
TCM: Traditional Chinese Medicine
TCMBank: Traditional Chinese Medicine Bank
TCMSP: Traditional Chinese Medicine Systems Pharmacology platform
TPM: Transcripts per million
