# Supplementary material for: Unveiling medication patterns in traditional Chinese medicine for the prevention of colorectal cancer recurrence: from potential combinations to validation of components and targets
Source: Chin Med. 2026 Jun 4;21:160. doi: 10.1186/s13020-026-01438-5 (PMC13235114; doi:10.1186/s13020-026-01438-5)
Supplement: Supplementary file 1 — Supplementary Material 1 [file 13020_2026_1438_MOESM1_ESM.docx]

**STable.1. TCM prescriptions for the prevention and treatment of CRC recurrence from RCTs, marketed Chinese patent drug and TCM combinations obtained a patent.**

| **Number** | **Identification number*** | **Herb** |
| --- | --- | --- |
| 1 | ChiMCTR2100004460 | *Puerariae Lobatae Radix, Scutellaria Baicalensis Radix, Coptis Chinensis Rhizoma, Glycyrrhiza Uralensis Radix Et Rhizoma* |
| 2 | ChiCTR-TRC-14004899 | *Astragalus Membranacei Radix, Atractylodes Macrocephalae Rhizoma, Rehmannia Glutinosa Radix Praeparata, Psoraleae Fructus, Amorphophallus rivieri Durieu, Massa Medicata Fermentata, Glycyrrhiza Uralensis Radix Et Rhizoma* |
| 3 | ChiCTR-IPR-15005846 | *Venenum Bufonis* |
| 4 | ChiCTR-INR-16008575 | *Panax Ginseng Radix Et Rhizoma, Atractylodes Macrocephalae Rhizoma, Poria Cocos, Glycyrrhiza Uralensis Radix Et Rhizoma, Pericarpium Citri Reticulatae Viride, Pinellia Ternata Rhizoma, Amomum Villosum Fructus, Aucklandia Lappa Radix* |
| 5 | ChiCTR-IOR-16007719 | *Astragalus Membranacei Radix, Codonopsis Pilosulae Radix, Aucklandia Lappa Radix, Polyporus, Pericarpium Citri Reticulatae Viride, Atractylodes Macrocephalae Rhizoma, Fructus Akebiae, Rehmannia Glutinosa Radix Praeparata, Sargentodoxae Caulis, Coix Lacryma-jobi Semen, Agrimoniae Herba, Vitis Coignetiae* |
| 6 | ChiCTR-IOR-16009739 | *Pericarpium Citri Reticulatae Viride, Pinellia Ternata Rhizoma, Bupleurum Chinense Radix, Poria Cocos, Atractylodes Macrocephalae Rhizoma, Astragalus Membranacei Radix, Coix Lacryma-jobi Semen, Actinidiae Radix, Hedyotis Diffusa Herba* |
| 7 | ChiCTR-IOR-17012337 | *Rehmannia Glutinosa Radix Praeparata, Cremastrae Pseudobulbus, Cistanches Herba, Ligustrum Lucidum Fructus, Fructus Akebiae, Duchesneae Indicae Herba* |
| 8 | ChiCTR1900027691 | *Ganoderma Lucidum* |
| 9 | ChiCTR2000033332 | *Coptis Chinensis Rhizoma, Euodia Rutaecarpa Fructus* |
| 10 | ChiCTR2000036541 | *Actinidiae Radix, Scutellaria Barbata Herba, Coix Lacryma-jobi Semen, Duchesneae Indicae Herba, Solani Nigri Herba, Poria Cocos, Atractylodes Macrocephalae Rhizoma, Visci Herba* |
| 11 | ChiCTR2000036635 | *Astragalus Membranacei Radix, Codonopsis Pilosulae Radix, Atractylodes Macrocephalae Rhizoma, Fructus Akebiae, Coix Lacryma-jobi Semen, Vitis Coignetiae* |
| 12 | ChiCTR2000037345 | *Astragalus Membranacei Radix, Codonopsis Pilosulae Radix, Atractylodes Macrocephalae Rhizoma, Poria Cocos, Glycyrrhiza Uralensis Radix Et Rhizoma, Pinellia Ternata Rhizoma, Scolopendra, Sargentodoxae Caulis, Actinidiae Radix, Ostreae Concha* |
| 13 | ChiCTR2000037693 | *Astragalus Membranacei Radix, Codonopsis Pilosulae Radix, Atractylodes Macrocephalae Rhizoma, Poria Cocos, Eupolyphaga Seu Steleophaga, Prunus Persica Semen, Scutellaria Barbata Herba, Hedyotis Diffusa Herba, Ganoderma Lucidum, Gynostemmatis Herba, Polygonatum Sibiricum Rhizoma* |
| 14 | ChiCTR2400085896 | *Astragalus Membranacei Radix, Sophora Flavescens Radix, Codonopsis Pilosulae Radix, Atractylodes Macrocephalae Rhizoma, Poria Cocos, Coix Lacryma-jobi Semen, Hedyotis Diffusa Herba, Scolopendra, Scolopendra, Glycyrrhiza Uralensis Radix Et Rhizoma* |
| 15 | ChiCTR2100050757 | *Astragalus Membranacei Radix, Panax Ginseng Radix Et Rhizoma, Atractylodes Macrocephalae Rhizoma, Bupleurum Chinense Radix, Angelica Sinensis Radix, Paeonia Lactiflora Radix Alba, Schisandrae Chinensis Fructus, Sparganii Rhizoma, Curcumae Rhizoma, Prunellae Spica, Curcumae Longae Rhizoma, Trionycis Carapax, Glycyrrhiza Uralensis Radix Et Rhizoma* |
| 16 | ChiCTR2100053521 | *Codonopsis Pilosulae Radix, Atractylodes Macrocephalae Rhizoma, Poria Cocos, Eupolyphaga Seu Steleophaga, Prunus Persica Semen, Scutellaria Barbata Herba, Hedyotis Diffusa Herba, Smilacis Glabrae Rhizoma, Curcumae Rhizoma, Aurantii Fructus* |
| 17 | NCT02796820 | *Huaier* |
| 18 | NCT05709249 | *Agrimoniae Herba, Coptis Chinensis Rhizoma, Sophora Flavescens Radix, Coix Lacryma-jobi Semen, Sparganii Rhizoma, Curcumae Rhizoma, Astragalus Membranacei Radix, Atractylodes Macrocephalae Rhizoma* |
| 19 | NCT03716063 | *Astragalus Membranacei Radix, Panax Ginseng Radix Et Rhizoma, Arisaematis Rhizoma, Ligustrum Lucidum Fructus, Poria Cocos, Epimedium Brevicornu Herba, Curcumae Longae Rhizoma, Salvia Miltiorrhiza Radix Et Rhizoma* |
| 20 | NCT02510118 | *Panax Ginseng Radix Et Rhizoma, Astragalus Membranacei Radix, Actinidiae Radix, Fructus Akebiae, Coix Lacryma-jobi Semen, Sophora Flavescens Radix* |
| 21 | Z52020236 | *Mylabris, Panax Ginseng Radix Et Rhizoma, Astragalus Membranacei Radix, Acanthopanacis Senticosi Radix et Rhizoma* |
| 22 | Z61021330 | *Curcumae Radix, Strychni Semen, Agrimoniae Herba, Trogopterorum Faeces, Alumen, Natrii Sulfas, Toxicodendri Resina, Aurantii Fructus* |
| 23 | Z52020238 | *Mylabris, Panax Ginseng Radix Et Rhizoma, Astragalus Membranacei Radix, Acanthopanacis Senticosi Radix et Rhizoma, Sparganii Rhizoma, Scutellaria Barbata Herba, Curcumae Rhizoma, Corni Fructus, Ligustrum Lucidum Fructus, Fel Ursi, Glycyrrhiza Uralensis Radix Et Rhizoma* |
| 24 | Z14021231 | *Sophora Flavescens Radix, Smilacis Chinae Rhizoma* |
| 25 | Z10980045 | *Leonuri Herba, Carthamus Tinctorius Flos, Zanthoxyli Pericarpium, Hirudo, Angelica Sinensis Radix, Sappan Lignum, Sparganii Rhizoma, Anemones Raddeanae Rhizoma, Ligusticum Chuanxiong Rhizoma, Lignum Dalbergiae Odoriferae, Cyperi Rhizoma, Panax Ginseng Radix Et Rhizoma, Alpiniae Officinarum Rhizoma, Curcumae Longae Rhizoma, Myrrha, Armeniacae Semen Amarum, Rheum Palmatum Radix Et Rhizoma, Moschus Artifactus, Foeniculi Fructus, Trogopterorum Faeces, Prunus Persica Semen, Tabanus, Trionycis Carapax, Caryophylli Flos, Corydalis Rhizoma, Paeonia Lactiflora Radix Alba, Typhae Pollen, Olibanum, Toxicodendri Resina, Euodia Rutaecarpa Fructus, Ferulae Resina, Cinnamomum Cassia Cortex, Artemisiae Argyi Folium, Perillae Fructus, Rehmannia Glutinosa Radix Praeparata* |
| 26 | Z20080468 | *Marsdeniae Tenacissimae Caulis* |
| 27 | Z20044247 | *Bruceae Fructus* |
| 28 | Z20026868 | *Astragalus Membranacei Radix, Panax Ginseng Radix Et Rhizoma, Sophora Flavescens Radix* |
| 29 | Z20025336 | *Cremastrae Pseudobulbus, Solanum Lyratum, Sophora Flavescens Radix, Epimedium Brevicornu Herba, Panax Ginseng Radix Et Rhizoma, Angelica Sinensis Radix, Atractylodes Macrocephalae Rhizoma* |
| 30 | Z20040121 | *Panax Ginseng Radix Et Rhizoma, Astragalus Membranacei Radix, Atractylodes Macrocephalae Rhizoma, Galli Gigerii Endothelium Corneum, Trichosanthis Fructus, Pinellia Ternata Rhizoma, Magnoliae Officinalis Cortex, Aurantii Fructus, Curcumae Radix, Salvia Miltiorrhiza Radix Et Rhizoma, Scorpio, Scolopendra* |
| 31 | Z20063321 | *Venenum Bufonis, Aconiti Radix, Carthamus Tinctorius Flos* |
| 32 | Z32021229 | *Polyporus* |
| 33 | Z20040086 | *Astragalus Membranacei Radix* |
| 34 | Z11020037 | *Codonopsis Pilosulae Radix, Lycium Barbarum Fructus, Ligustrum Lucidum Fructus, Atractylodes Macrocephalae Rhizoma, Cuscutae Semen, Psoraleae Fructus* |
| 35 | Z20025075 | *Ferulae Resina, Aspongopus, Rheum Palmatum Radix Et Rhizoma, Curcumae Longae Rhizoma, Chebulae Fructus, Aucklandia Lappa Radix, Caryophylli Flos, Ophiocordyceps Sinensis* |
| 36 | Z20113005 | *Epimedium Brevicornu Herba, Psoraleae Fructus, Aconitum Carmichaelii Radix Praeparata, Lycium Barbarum Fructus, Astragalus Membranacei Radix, Spatholobus Suberectus Caulis, Rubiae Radix et Rhizoma, Angelica Sinensis Radix, Phragmitis Rhizoma, Ophiopogonis Radix, Glycyrrhiza Uralensis Radix Et Rhizoma* |
| 37 | Z22021226 | *Angelica Sinensis Radix, Astragalus Membranacei Radix, Cornu Cervi Colla, Asini Corii Colla, Ziziphus Jujuba Fructus* |
| 38 | Z10970042 | *Ginseng Radix et Rhizoma Rubra, Astragalus Membranacei Radix, Lycium Barbarum Fructus, Ligustrum Lucidum Fructus, Polyporus, Poria Cocos* |
| 39 | CN118831132A | *Pseudostellariae Radix,Atractylodes Macrocephalae Rhizoma, Astragalus Membranacei Radix, Polygonatum Sibiricum Rhizoma, Ganoderma Lucidum, Rehmannia Glutinosa Radix Praeparata, Smilacis Chinae Rhizoma, Vitis Coignetiae, Hedyotis Diffusa Herba, Gynostemmatis Herba* |
| 40 | CN118416178A | *Rehmannia Glutinosa Radix Praeparata, Angelica Sinensis Radix, Paeonia Lactiflora Radix Alba, Ligusticum Chuanxiong Rhizoma, Epimedium Brevicornu Herba, Crinis Carbonisatus, Codonopsis Pilosulae Radix, Pericarpium Citri Reticulatae Viride, Zanthoxyli Pericarpium, Coix Lacryma-jobi Semen, Amomum Villosum Fructus, Glycyrrhiza Uralensis Radix Et Rhizoma* |
| 41 | CN118356475A | *Paeonia Lactiflora Radix Alba, Glycyrrhiza Uralensis Radix Et Rhizoma, Aconitum Carmichaelii Radix Praeparata, Scutellaria Baicalensis Radix, Sophora Flavescens Radix, Rehmanniae Radix, Ziziphi Spinosae Semen, Zingiber Officinale Rhizoma, Ostreae Concha, Amomum Villosum Fructus, Poria, Pheretima* |
| 42 | CN116370594A | *Codonopsis Pilosulae Radix, Atractylodes Macrocephalae Rhizoma, Poria Cocos, Astragalus Membranacei Radix, Coix Lacryma-jobi Semen, Ganoderma Lucidum, Epimedium Brevicornu Herba, Salvia Miltiorrhiza Radix Et Rhizoma, Hedyotis Diffusa Herba, Paridis Rhizoma, Pinellia Ternata Rhizoma, Scutellaria Barbata Herba, Rehmannia Glutinosa Radix Praeparata, Curcumae Rhizoma, Aucklandia Lappa Radix, Glycyrrhiza Uralensis Radix Et Rhizoma, Coix Lacryma-jobi Semen, Poria Cocos, Curcumae Radix, Hedyotis Diffusa Herba, Patriniae Herba, Sophora Flavescens Radix, Magnoliae Officinalis Cortex, Atractylodes Lancea Rhizoma, Borneolum* |
| 43 | CN115300592A | *Codonopsis Pilosulae Radix, Poria Cocos, Atractylodes Macrocephalae Rhizoma, Coix Lacryma-jobi Semen, Amomum Villosum Fructus, Lablab Purpureus, Dioscoreae Rhizoma, Euryales Semen, Glycyrrhiza Uralensis Radix Et Rhizoma, Paeoniae Radix Rubra, Aurantii Fructus, Bupleurum Chinense Radix, Hedyotis Diffusa Herba, Pulsatillae Radix, Nelumbinis Semen* |
| 44 | CN117018153A | *Hedyotis Diffusa Herba, Paridis Rhizoma,Sophora Flavescens Radix, Taraxacum Mongolicum Herba, Curcumae Longae Rhizoma, Myristicae Semen, Magnoliae Officinalis Cortex, Poria Cocos, Curcumae Radix, Onosma Paniculatum, Atractylodes Macrocephalae Rhizoma, Paeoniae Radix Rubra, Viticis Fructus, Platycodonis Radix, Polygoni Cuspidati Rhizoma et Radix, Cnidii Fructus, Forsythia Suspensa Fructus, Cimicifugae Rhizoma, Euodia Rutaecarpa Fructus, Cinnamomum Cassia Ramulus, Zingiber Officinale Rhizoma Recens* |
| 45 | CN114588240A | *Borax, Actinidiae Radix, Vitis Coignetiae, Fructus Polygoni Orientalis, Ficus Pumila, Curcumae Rhizoma, Hedyotis Diffusa Herba, Paecilomyces Cicadae, Scolopendra, Rehmannia Glutinosa Radix Praeparata, Duchesneae Indicae Herba, Sparganii Rhizoma, Selaginella Doederleinii, Smilacis Chinae Rhizoma, Trichosanthis Fructus, Ganoderma Lucidum, Scolopendra, Citrus Aurantium Fructus Immaturus* |
| 46 | CN113925952A | *Coptis Chinensis Rhizoma, Zingiberis Rhizoma Preparatum, Indigo Naturalis, Sophora Flavescens Radix, Notoginseng Radix et Rhizoma, Aucklandia Lappa Radix, Sanguisorbae Radix, Glycyrrhiza Uralensis Radix Et Rhizoma* |
| 47 | CN115154534A | *Pseudostellariae Radix,Poria Cocos,Pinellia Ternata Rhizoma,Sargentodoxae Caulis,Ophiocordyceps Sinensis,Prunus Mume Flos,Ligustrum Lucidum Fructus* |
| 48 | CN109674925A | *Armeniacae Semen Amarum, Schisandrae Chinensis Fructus, Euodia Rutaecarpa Fructus, Ranunculi Ternati Radix, Astragalus Membranacei Radix, Atractylodes Macrocephalae Rhizoma, Corni Fructus, Bulbus Allii, Panacis Quinquefolii Radix, Sophora Flavescens Radix, Pericarpium Citri Reticulatae Viride, Arctii Fructus, Bupleurum Chinense Radix, Scutellaria Baicalensis Radix, Rabdosiae Rubescentis Herba, Saposhnikovia Divaricata Radix, Cistanches Herba, Zanthoxyli Pericarpium, Astragali Complanati Semen, Cynomorii Herba, Dioscoreae Rhizoma, Scutellaria Barbata Herba, Hedyotis Diffusa Herba, Crataegi Fructus* |
| 49 | CN107551254A | *Hedyotis Diffusa Herba, Sophora Flavescens Radix, Codonopsis Pilosulae Radix, Atractylodes Macrocephalae Rhizoma, Mume Fructus, Coptis Chinensis Rhizoma, Zingiberis Rhizoma Preparatum, Coix Lacryma-jobi Semen* |
| 50 | CN108324880A | *Scolopendra, Scorpio, Radix Seu Herba Lobeliae Sessilifoliae, Phellodendron Chinense Cortex, Sparganii Rhizoma, Rheum Palmatum Radix Et Rhizoma, Arisaema Cum Bile, Sargassum, Astragalus Membranacei Radix, Dioscoreae Rhizoma* |
| 51 | CN106390013A | *Astragalus Membranacei Radix, Codonopsis Pilosulae Radix, Atractylodes Macrocephalae Rhizoma, Poria Cocos, Dioscoreae Rhizoma, Glycyrrhiza Uralensis Radix Et Rhizoma, Mume Fructus, Sparganii Rhizoma, Curcumae Rhizoma, Agrimoniae Herba, Patriniae Herba* |
| 52 | CN105920534A | *Zaocys, Coix Lacryma-jobi Semen, Trichosanthis Fructus, Scorpio, Scolopendra, Galli Gigerii Endothelium Corneum, Sparganii Rhizoma, Curcumae Rhizoma, Borax, Gleditsiae Sinensis Fructus, Indigo Naturalis* |
| 53 | CN105832933A | *Andrographis Paniculata Herba, Hirudo, Pinellia Ternata Rhizoma, Radix Tripterygii Hypoglauci Pareter Corticem, Ganoderma Lucidum, Gardenia Jasminoides Fructus, Rabdosiae Rubescentis Herba, Resina Liquidambaris, Notoginseng Radix et Rhizoma, Polygoni Multiflori Radix, Sanguisorbae Radix, Agrimoniae Herba, Hippocampus, Menthae Haplocalycis Herba* |
| 54 | CN105456949A | *Codonopsis Pilosulae Radix, Astragalus Membranacei Radix, Atractylodes Macrocephalae Rhizoma, Epimedium Brevicornu Herba, Coix Lacryma-jobi Semen, Pinellia Ternata Rhizoma, Taraxacum Mongolicum Herba, Ostreae Concha, Curcumae Rhizoma, Actinidiae Radix* |
| 55 | CN105396068A | *Codonopsis Pilosulae Radix, Dioscoreae Rhizoma, Astragalus Membranacei Radix, Coix Lacryma-jobi Semen, Curcumae Rhizoma, Smilacis Glabrae Rhizoma, Scutellaria Barbata Herba, Alismatis Rhizoma, Rheum Palmatum Radix Et Rhizoma, Chebulae Fructus, Eupatorii Herba, Scrophulariae Radix, Corydalis Rhizoma, Pinellia Ternata Rhizoma, Olibanum, Myrrha, , Duchesneae Indicae Herba, Sargassum, Tsaoko Fructus, Arcae Concha, Pumex, Indigo Naturalis* |
| 56 | CN104127798A | *Astragalus Membranacei Radix, Ligustrum Lucidum Fructus, Panax Ginseng Radix Et Rhizoma, Ganoderma Lucidum, Curcumae Rhizoma, Atractylodes Macrocephalae Rhizoma, Scutellaria Barbata Herba, Gynostemmatis Herba, Poria Cocos, Galli Gigerii Endothelium Corneum, Duchesneae Indicae Herba, Solanum Lyratum, Artemisiae Scopariae Herba, Cynanchi Paniculati Radix et Rhizoma, Eupolyphaga Seu Steleophaga, Hedyotis Diffusa Herba* |
| 57 | 10.3969/j.issn.1004-0412.2011.36.010 | *Rheum Palmatum Radix Et Rhizoma, Phellodendron Chinense Cortex, Paeoniae Radix Rubra, Carthamus Tinctorius Flos, Sophora Flavescens Radix, Taraxacum Mongolicum Herba, Paris Tetraphylla, Sanguisorbae Radix,Prunus Persica Semen, Aucklandia Lappa Radix, Coptis Chinensis Rhizoma, Sargentodoxae Caulis, Pulsatillae Radix, Sparganii Rhizoma,Curcumae Rhizoma,Citrus Aurantium Fructus Immaturus,Fructus Akebiae* |
| 58 | 0253-9934(2014)11-984-986 | *Astragalus Membranacei Radix, Atractylodes Macrocephalae Rhizoma, Corni Fructus, Rehmannia Glutinosa Radix Praeparata, Psoraleae Fructus, Amorphophallus rivieri Durieu, Vitis Coignetiae, Curcumae Rhizoma, Crataegi Fructus, Massa Medicata Fermentata, Glycyrrhiza Uralensis Radix Et Rhizoma* |
| 59 | 10.13862/j.cnki.cn43-1446/r.2014.05.009 | *Panacis Quinquefolii Radix, Astragalus Membranacei Radix, Dioscoreae Rhizoma, Poria Cocos, Ganoderma Lucidum, Lycium Barbarum Fructus, Cuscutae Semen, Ligustrum Lucidum Fructus, Prunellae Spica, Hedyotis Diffusa Herba, Glycyrrhiza Uralensis Radix Et Rhizoma, Crataegi Fructus* |
| 60 | 10.1097/MD.0000000000027850 | *Puerariae Lobatae Radix,Scutellaria Baicalensis Radix,Coptis Chinensis Rhizoma,Aucklandia Lappa Radix,Pericarpium Citri Reticulatae Viride,Benincasae Semen,Zingiberis Rhizoma Preparatum* |
| 61 | 10.26988/d.cnki.gcdzu.2022.000541 | *Pinellia Ternata Rhizoma,Zingiber Officinale Rhizoma,Panax Ginseng Radix Et Rhizoma ,Scutellaria Baicalensis Radix ,Coptis Chinensis Rhizoma,Euodia Rutaecarpa Fructus,Cinnamomum Cassia Ramulus,Poria Cocos* |
| 62 | 10.3969/j.issn.1005-7072.2022.3. zgzyykj202203015 | *Astragalus Membranacei Radix,Ganoderma Lucidum,Hedyotis Diffusa Herba,Scorpio,Prunellae Spica,Curcumae Rhizoma* |
| 63 | 10.13457/j.cnki.jncm.2014.01.093 | *Astragalus Membranacei Radix, Coix Lacryma-jobi Semen, Hedyotis Diffusa Herba, Codonopsis Pilosulae Radix, Atractylodes Macrocephalae Rhizoma, Poria Cocos, Pinellia Ternata Rhizoma, Pericarpium Citri Reticulatae Viride, Fructus Akebiae, Fritillariae Thunbergii Bulbus, Actinidiae Radix* |
| 64 | 10.13359/j.cnki.gzxbtcm.2024.10.030 | *Astragalus Membranacei Radix, Codonopsis Pilosulae Radix, Atractylodes Macrocephalae Rhizoma, Angelica Sinensis Radix, Pericarpium Citri Reticulatae Viride, Cimicifugae Rhizoma, Bupleurum Chinense Radix, Glycyrrhiza Uralensis Radix Et Rhizoma, Pulsatillae Radix, Fraxini Cortex, Phellodendron Chinense Cortex, Coptis Chinensis Rhizoma, Curcumae Longae Rhizoma, Curcumae Radix* |
| 65 | 10.3969/j.issn.1003-8914.2024.01.032 | *Astragalus Membranacei Radix, Coix Lacryma-jobi Semen, Spatholobus Suberectus Caulis, Agrimoniae Herba, Hedyotis Diffusa Herba, Patriniae Herba, Atractylodes Macrocephalae Rhizoma, Patriniae Radix et Rhizoma, Curcumae Rhizoma, Sinapis Semen, Panax Ginseng Radix Et Rhizoma* |
| 66 | 10.26922/d.cnki.ganzc.2024.000119 | *Panax Ginseng Radix Et Rhizoma, Glycyrrhiza Uralensis Radix Et Rhizoma, Atractylodes Macrocephalae Rhizoma, Astragalus Membranacei Radix, Poria Cocos, Hedyotis Diffusa Herba, Fructus Akebiae, Cremastrae Pseudobulbus* |
| 67 | 10.3969/j.issn.1006-6810.2023.06.004 | *Glycyrrhiza Uralensis Radix Et Rhizoma, Citrus Aurantium Fructus Immaturus, Dioscoreae Rhizoma, Coix Lacryma-jobi Semen, Astragalus Membranacei Radix, Scorpio, Scolopendra, Cremastrae Pseudobulbus, Poria Cocos, Galli Gigerii Endothelium Corneum, Arisaematis Rhizoma, Pinellia Ternata Rhizoma* |
| 68 | 10.13457/j.cnki.jncm.2023.05.034 | *Scorpio, Scolopendra, Panax Ginseng Radix Et Rhizoma, Cervi Cornu Pantotrichum, Rheum Palmatum Radix Et Rhizoma, Scolopendra, Notoginseng Radix et Rhizoma, Caulis Bambusae in Taeniam, Pericarpium Citri Reticulatae Viride, Natrii Sulfas, Glycyrrhiza Uralensis Radix Et Rhizoma* |
| 69 | 10.19613/j.cnki.1671-3141.2019.88.115 | *Cimicifugae Rhizoma, Poria Cocos, Paeonia Lactiflora Radix Alba, Squama Manis, Codonopsis Pilosulae Radix, Eriobotryae Folium, Astragalus Membranacei Radix, Ligustrum Lucidum Fructus, Galli Gigerii Endothelium Corneum, Lycium Barbarum Fructus, Cuscutae Semen, Hedyotis Diffusa Herba, Pericarpium Citri Reticulatae Viride, Polygonatum Sibiricum Rhizoma, Coix Lacryma-jobi Semen, Polyporus, Angelica Sinensis Radix, Ligusticum Chuanxiong Rhizoma, Actinidiae Radix, Portulacae Herba, Atractylodes Macrocephalae Rhizoma, Rehmannia Glutinosa Radix Praeparata, Paris Tetraphylla, Pinellia Ternata Rhizoma, Glycyrrhiza Uralensis Radix Et Rhizoma* |
| 70 | 10.13457/j.cnki.jncm.2019.08.059 | *Paeonia Lactiflora Radix Alba, Coix Lacryma-jobi Semen, Pseudostellariae Radix, Hordei Fructus Germinatus, Crataegi Fructus, Massa Medicata Fermentata, Scutellaria Barbata Herba, Sophora Flavescens Radix, Solani Nigri Herba, Astragalus Membranacei Radix, Curcumae Radix, Radix Seu Herba Lobeliae Sessilifoliae, Polygoni Cuspidati Rhizoma et Radix, Atractylodes Macrocephalae Rhizoma, Portulacae Herba, Magnoliae Officinalis Cortex, Hedyotis Diffusa Herba, Salvia Miltiorrhiza Radix Et Rhizoma, Rheum Palmatum Radix Et Rhizoma, Glycyrrhiza Uralensis Radix Et Rhizoma* |
| 71 | 10.3969/j.issn.1005-5304.2018.12.007 | *Astragalus Membranacei Radix, Pseudostellariae Radix, Atractylodes Macrocephalae Rhizoma, Poria Cocos, Glycyrrhiza Uralensis Radix Et Rhizoma, Coix Lacryma-jobi Semen, Hedyotis Diffusa Herba, Scutellaria Barbata Herba, Curcumae Rhizoma, Pinellia Ternata Rhizoma, Pericarpium Citri Reticulatae Viride, Ramulus Taxi Cuspidatae, Gleditsiae Sinensis Fructus* |
| 72 | 10.3969/j.issn.1672-2671.2018.02.018 | *Astragalus Membranacei Radix, Codonopsis Pilosulae Radix, Poria Cocos, Corni Fructus, Atractylodes Macrocephalae Rhizoma, Pericarpium Citri Reticulatae Viride, Rehmannia Glutinosa Radix Praeparata, Aucklandia Lappa Radix, Cinnamomum Cassia Cortex, Lycium Barbarum Fructus, Polygonatum Sibiricum Rhizoma, Angelica Sinensis Radix, Pinellia Ternata Rhizoma, Coptis Chinensis Rhizoma, Scutellaria Baicalensis Radix, Glycyrrhiza Uralensis Radix Et Rhizoma* |
| 73 | 10.16424/j.cnki.cn32-1807/r.2018.01.010 | *Codonopsis Pilosulae Radix, Astragalus Membranacei Radix, Dioscoreae Rhizoma, Drynariae Rhizoma, Atractylodes Macrocephalae Rhizoma, Ganoderma Lucidum, Gynostemmatis Herba, Poria Cocos, Polyporus, Coix Lacryma-jobi Semen, Taxus chinensis, Actinidiae Radix, Hedyotis Diffusa Herba, Polygoni Cuspidati Rhizoma et Radix, Curcumae Rhizoma* |
| 74 | 1000-3649(2017)11-0095-03 | *Pseudostellariae Radix, Astragalus Membranacei Radix, Atractylodes Macrocephalae Rhizoma, Poria Cocos, Coix Lacryma-jobi Semen, Scutellaria Barbata Herba, Curcumae Rhizoma, Salvia Miltiorrhiza Radix Et Rhizoma, Hedyotis Diffusa Herba, Agrimoniae Herba* |
| 75 | 1672-397X (2017)05-0036-04 | *Astragalus Membranacei Radix, Atractylodes Macrocephalae Rhizoma, Pericarpium Citri Reticulatae Viride, Hordei Fructus Germinatus, Galli Gigerii Endothelium Corneum, Coix Lacryma-jobi Semen, Cyperi Rhizoma, Curcumae Radix, Curcumae Rhizoma, Eupolyphaga Seu Steleophaga, Scutellaria Barbata Herba, Hedyotis Diffusa Herba, Scolopendra, Glycyrrhiza Uralensis Radix Et Rhizoma* |
| 76 | 10.13193/j.issn.1673 - 7717.2017.02.013 | *Actinidiae Radix, Polygoni Cuspidati Rhizoma et Radix, Radix Adinae Rubellae, Atractylodes Macrocephalae Rhizoma, Codonopsis Pilosulae Radix, Poria Cocos, Coix Lacryma-jobi Semen, Glycyrrhiza Uralensis Radix Et Rhizoma* |
| 77 | 10.11986/j.issn.1673-873X.2015.06.017 | *Astragalus Membranacei Radix, Coix Lacryma-jobi Semen, Spatholobus Suberectus Caulis, Hedyotis Diffusa Herba, Puerariae Lobatae Radix, Agrimoniae Herba, Atractylodes Macrocephalae Rhizoma, Patriniae Radix et Rhizoma, Sinapis Semen, Curcumae Rhizoma* |
| 78 | 10.13935/j.cnki.sjzx.150735 | *Fritillariae Thunbergii Bulbus, Codonopsis Pilosulae Radix, Atractylodes Macrocephalae Rhizoma, Coix Lacryma-jobi Semen, Spatholobus Suberectus Caulis, Polygonatum Sibiricum Rhizoma, Angelica Sinensis Radix, Lycium Barbarum Fructus, Amomum Villosum Fructus* |
| 79 | 10.16367/j.issn.1003-5028.2014.07.058 | *Codonopsis Pilosulae Radix, Astragalus Membranacei Radix, Acanthopanacis Senticosi Radix et Rhizoma,Poria Cocos, Atractylodes Macrocephalae Rhizoma, Paeonia Lactiflora Radix Alba, Ligustrum Lucidum Fructus, Lycium Barbarum Fructus, Angelica Sinensis Radix, Crataegi Fructus, Ziziphus Jujuba Fructus* |
| 80 | 10.13193/j.issn.1673-7717.2014.03.076 | *Codonopsis Pilosulae Radix, Atractylodes Macrocephalae Rhizoma, Poria Cocos, Astragalus Membranacei Radix, Coix Lacryma-jobi Semen, Pinellia Ternata Rhizoma, Fritillariae Thunbergii Bulbus, Dioscoreae Rhizoma, Actinidiae Radix, Hedyotis Diffusa Herba, Trionycis Carapax, Glycyrrhiza Uralensis Radix Et Rhizoma* |
| 81 | 10.16368/j.issn.1674-8999.2013.10.040 | *Astragalus Membranacei Radix, Pinellia Ternata Rhizoma, Pericarpium Citri Reticulatae Viride, Atractylodes Macrocephalae Rhizoma, Pseudostellariae Radix, Sargentodoxae Caulis, Patriniae Herba* |
| 82 | 10.16295/j.cnki.0257-358x.2013.07.036 | *Astragalus Membranacei Radix, Atractylodes Macrocephalae Rhizoma, Sargentodoxae Caulis, Codonopsis Pilosulae Radix, Poria Cocos, Coix Lacryma-jobi Semen, Magnoliae Officinalis Cortex, Paeonia Lactiflora Radix Alba, Cortex Toonae Sinensis, Myristicae Semen, Herba Artemisiae Anomalae, Scutellaria Barbata Herba, Hedyotis Diffusa Herba, Polygonatum Sibiricum Rhizoma, Glycyrrhiza Uralensis Radix Et Rhizoma* |
| 83 | 10.3969/j.issn.1002-2619.2013.01.039 | *Codonopsis Pilosulae Radix, Atractylodes Macrocephalae Rhizoma, Poria Cocos, Paeonia Lactiflora Radix Alba, Bupleurum Chinense Radix, Toosendan Fructus, Citrus Medica Var. Sarcodactylis Fructus, Dioscoreae Rhizoma, Curcumae Rhizoma, Vitis Coignetiae, Sargentodoxae Caulis, Prunus Mume Flos* |
| 84 | 10.3969/j.issn.1674-7860.2012.17.001 | *Astragalus Membranacei Radix, Codonopsis Pilosulae Radix, Coix Lacryma-jobi Semen, Atractylodes Macrocephalae Rhizoma, Poria Cocos, Pericarpium Citri Reticulatae Viride, Aucklandia Lappa Radix, Hedyotis Diffusa Herba, Scutellaria Barbata Herba, Taraxacum Mongolicum Herba, Cynanchi Paniculati Radix et Rhizoma* |
| 85 | 10.13192/j.ljtcm.2012.05.85.laijch.010 | *Astragalus Membranacei Radix, Pseudostellariae Radix, Dioscoreae Rhizoma, Coix Lacryma-jobi Semen, Atractylodes Macrocephalae Rhizoma, Poria Cocos, Lablab Purpureus, Hedyotis Diffusa Herba, Radix Seu Herba Lobeliae Sessilifoliae, Scutellaria Barbata Herba, Phellodendron Chinense Cortex, Puerariae Lobatae Radix, Paeonia Suffruticosa Cortex, Salvia Miltiorrhiza Radix Et Rhizoma* |
| 86 | 10.3969/j.issn.1672-397X.2011.11.010 | *Panacis Quinquefolii Radix, Astragalus Membranacei Radix, Angelica Sinensis Radix, Atractylodes Macrocephalae Rhizoma, Poria Cocos, Trogopterorum Faeces, Myrrha, Notoginseng Radix et Rhizoma, Paeonia Lactiflora Radix Alba, Actinidiae Radix, Endoconcha Sepiae, Arcae Concha, Glycyrrhiza Uralensis Radix Et Rhizoma* |
| 87 | 10.13193/j.archtcm.2011.01.156.dongjb.074 | *Pseudostellariae Radix, Atractylodes Macrocephalae Rhizoma, Poria Cocos, Glycyrrhiza Uralensis Radix Et Rhizoma, Fritillariae Thunbergii Bulbus, Platycodonis Radix, Coix Lacryma-jobi Semen, Curcumae Rhizoma, Ficus Pumila* |
| 88 | 10.3760/cma.j.issn.1673-4246.2006.05.012 | *Astragalus Membranacei Radix, Pseudostellariae Radix, Spatholobus Suberectus Caulis, Atractylodes Macrocephalae Rhizoma, Poria Cocos, Lycium Barbarum Fructus, Ligustrum Lucidum Fructus, Cuscutae Semen, Psoraleae Fructus, Paeoniae Radix Rubra, Hirudo* |
| 89 | 10.3969/j.issn.1672-397X.2005.01.006 | *Bruceae Fructus, Camptotheca Acuminata, Curcumae Rhizoma, Sargentodoxae Caulis, Codonopsis Pilosulae Radix* |
| 90 | 1009-8771(2016)01-0069-04 | *Codonopsis Pilosulae Radix, Astragalus Membranacei Radix, Atractylodes Macrocephalae Rhizoma, Poria Cocos, Eupolyphaga Seu Steleophaga, Prunus Persica Semen, Curcumae Rhizoma, Sarcandrae Herba, Hedyotis Diffusa Herba, Aurantii Fructus* |
| 91 | 10.27044/d.cnki.ggzzu.2023.001021 | *Hedyotis Diffusa Herba, Scutellaria Barbata Herba, Smilacis Glabrae Rhizoma, Curcumae Rhizoma, Prunus Persica Semen, Eupolyphaga Seu Steleophaga, Codonopsis Pilosulae Radix, Atractylodes Macrocephalae Rhizoma, Poria Cocos, Aurantii Fructus, Glycyrrhiza Uralensis Radix Et Rhizoma* |
| 92 | 1672-835(2017)09-0010-02 | *Coix Lacryma-jobi Semen, Astragalus Membranacei Radix, Pericarpium Citri Reticulatae Viride, Atractylodes Macrocephalae Rhizoma, Glycyrrhiza Uralensis Radix Et Rhizoma, Fructus Akebiae, Poria Cocos, Codonopsis Pilosulae Radix, Fritillariae Thunbergii Bulbus, Pinellia Ternata Rhizoma* |
| 93 | NJUCM-MS-2017-WJM | *Codonopsis Pilosulae Radix, Astragalus Membranacei Radix, Atractylodes Macrocephalae Rhizoma, Polygonatum Sibiricum Rhizoma, Polygonati Odorati Rhizoma, Poria Cocos, Coix Lacryma-jobi Semen, Aucklandia Lappa Radix, Curcumae Rhizoma, Actinidiae Radix, Hedyotis Diffusa Herba* |
| 94 | 10.11954/ytctyy.201708054 | *Pinellia Ternata Rhizoma, Scutellaria Baicalensis Radix, Codonopsis Pilosulae Radix, Zingiber Officinale Rhizoma, Glycyrrhiza Uralensis Radix Et Rhizoma, Coptis Chinensis Rhizoma, Ziziphus Jujuba Fructus* |
| 95 | 2096-2304(2018)07(03)-151-152 | *Angelica Sinensis Radix, Dioscoreae Rhizoma, Atractylodes Macrocephalae Rhizoma, Pinellia Ternata Rhizoma, Coix Lacryma-jobi Semen, Curcumae Rhizoma, Sophora Flavescens Radix, Fritillariae Thunbergii Bulbus, Patriniae Herba, Codonopsis Pilosulae Radix, Poria Cocos, Solani Nigri Herba, Glycyrrhiza Uralensis Radix Et Rhizoma, Pericarpium Citri Reticulatae Viride* |
| 96 | 10.16548/j.2095-3720.2018.06.011 | *Astragalus Membranacei Radix, Corni Fructus, Zingiber Officinale Rhizoma, Acori Tatarinowii Rhizoma, Solani Nigri Herba, Pericarpium Citri Reticulatae Viride, Aconitum Carmichaelii Radix Praeparata, Poria Cocos, Scutellaria Barbata Herba, Psoraleae Fructus, Salvia Miltiorrhiza Radix Et Rhizoma, Paeonia Lactiflora Radix Alba, Curcumae Radix, Agrimoniae Herba* |
| 97 | 10.3969/j.issn.1003-8914.2016.08.003 | *Astragalus Membranacei Radix, Codonopsis Pilosulae Radix, Smilacis Glabrae Rhizoma, Hedyotis Diffusa Herba, Patriniae Herba, Sparganii Rhizoma, Curcumae Rhizoma, Glycyrrhiza Uralensis Radix Et Rhizoma* |
| 98 | 10.3969/j.issn.1000-7369.2024.02.010 | *Cornu Cervi Colla, Cinnamomum Cassia Cortex, Zingiber Officinale Rhizoma, Atractylodes Macrocephalae Rhizoma, Ephedrae Herba, Poria Cocos, Aconitum Carmichaelii Radix Praeparata, Rehmannia Glutinosa Radix Praeparata, Astragalus Membranacei Radix, Glycyrrhiza Uralensis Radix Et Rhizoma, Euodia Rutaecarpa Fructus* |
| 99 | 10.27753/d.cnki.gcqgx.2024.001277 | *Galli Gigerii Endothelium Corneum, Codonopsis Pilosulae Radix, Poria Cocos, Glycyrrhiza Uralensis Radix Et Rhizoma, Crataegi Fructus, Hedyotis Diffusa Herba, Garcinia Hanburyi, Curcumae Longae Rhizoma, Rheum Palmatum Radix Et Rhizoma, Astragalus Membranacei Radix, Angelica Sinensis Radix , Atractylodes Macrocephalae Rhizoma, Polygoni Multiflori Radix, Cistanches Herba, Armeniacae Semen Amarum, Paeonia Lactiflora Radix Alba, Cannabis Semen, Aurantii Fructus, Magnoliae Officinalis Cortex, Glycyrrhiza Uralensis Radix Et Rhizoma, Pericarpium Citri Reticulatae Viride, Zingiber Officinale Rhizoma Recens, Angelica Sinensis Radix* |
| 100 | 10.3969/j.issn.1674-070X.2024.07.011 | *Curcumae Radix, Astragalus Membranacei Radix, Aurantii Fructus, Poria Cocos, Hedyotis Diffusa Herba, Epimedium Brevicornu Herba, Atractylodes Macrocephalae Rhizoma, Scutellaria Barbata Herba, Pinellia Ternata Rhizoma, Panax Ginseng Radix Et Rhizoma, Glycyrrhiza Uralensis Radix Et Rhizoma* |
| 101 | 10.3969/j.issn.1008-7664.2014.08.057 | *Ginseng Radix et Rhizoma, Poria Cocos, Atractylodes Macrocephalae Rhizoma, Dioscoreae Rhizoma, Dendrobii Caulis, Angelica Sinensis Radix, Paeonia Lactiflora Radix Alba, Glycyrrhiza Uralensis Radix Et Rhizoma* |
| 102 | 10.13241/j.cnki.pmb.2023.04.013 | *Codonopsis Pilosulae Radix, Astragalus Membranacei Radix, Atractylodes Macrocephalae Rhizoma, Rehmannia Glutinosa Radix Praeparata, Poria Cocos, Angelica Sinensis Radix, Paeonia Lactiflora Radix Alba, Ligusticum Chuanxiong Rhizoma, Pericarpium Citri Reticulatae Viride, Bupleurum Chinense Radix, Cimicifugae Rhizoma, Fructus Akebiae, Galli Gigerii Endothelium Corneum, Crataegi Fructus, Glycyrrhiza Uralensis Radix Et Rhizoma* |

^*^Identification number includes Registration Number, Drug approval number, Patent Number and DOI Number

**STable.2. A total of 148 valid rules based on Apriori association rule.**

| **Rules** | **Support** | **Confidence** | **Coverage** | **Lift** | **Count** |
| --- | --- | --- | --- | --- | --- |
| *Atractylodes Macrocephalae Rhizoma, Poria Cocos, Astragalus Membranacei Radix, Coix Lacryma-jobi Semen→Hedyotis Diffusa Herba* | 0.12 | 0.92 | 0.13 | 2.82 | 12 |
| *Atractylodes Macrocephalae Rhizoma, Scutellaria Barbata Herba, Astragalus Membranacei Radix→Hedyotis Diffusa Herba* | 0.11 | 0.92 | 0.12 | 2.8 | 11 |
| *Codonopsis Pilosulae Radix, Poria Cocos, Astragalus Membranacei Radix, Coix Lacryma-jobi Semen→Hedyotis Diffusa Herba* | 0.09 | 0.9 | 0.1 | 2.75 | 9 |
| *Atractylodes Macrocephalae Rhizoma, Codonopsis Pilosulae Radix, Poria Cocos, Astragalus Membranacei Radix, Coix Lacryma-jobi Semen→Hedyotis Diffusa Herba* | 0.09 | 0.9 | 0.1 | 2.75 | 9 |
| *Atractylodes Macrocephalae Rhizoma, Scutellaria Barbata Herba→Hedyotis Diffusa Herba* | 0.13 | 0.87 | 0.14 | 2.65 | 13 |
| *Poria Cocos, Astragalus Membranacei Radix, Coix Lacryma-jobi Semen→Hedyotis Diffusa Herba* | 0.13 | 0.87 | 0.14 | 2.65 | 13 |
| *Atractylodes Macrocephalae Rhizoma, Scutellaria Barbata Herba, Poria Cocos→Hedyotis Diffusa Herba* | 0.1 | 0.83 | 0.12 | 2.55 | 10 |
| *Atractylodes Macrocephalae Rhizoma, Actinidiae Radix→Coix Lacryma-jobi Semen* | 0.09 | 0.82 | 0.11 | 2.5 | 9 |
| *Scutellaria Barbata Herba, Poria Cocos, Astragalus Membranacei Radix→Hedyotis Diffusa Herba* | 0.09 | 0.82 | 0.11 | 2.5 | 9 |
| *Atractylodes Macrocephalae Rhizoma, Poria Cocos, Glycyrrhiza Uralensis Radix Et Rhizoma, Coix Lacryma-jobi Semen→Codonopsis Pilosulae Radix* | 0.09 | 0.82 | 0.11 | 2.36 | 9 |
| *Pinellia Ternata Rhizoma, Glycyrrhiza Uralensis Radix Et Rhizoma, Astragalus Membranacei Radix→Poria Cocos* | 0.09 | 1 | 0.09 | 2.21 | 9 |
| *Atractylodes Macrocephalae Rhizoma, Pinellia Ternata Rhizoma, Glycyrrhiza Uralensis Radix Et Rhizoma→Poria Cocos* | 0.1 | 1 | 0.1 | 2.21 | 10 |
| *Hedyotis Diffusa Herba, Codonopsis Pilosulae Radix, Astragalus Membranacei Radix, Coix Lacryma-jobi Semen→Poria Cocos* | 0.09 | 1 | 0.09 | 2.21 | 9 |
| *Atractylodes Macrocephalae Rhizoma, Codonopsis Pilosulae Radix, Glycyrrhiza Uralensis Radix Et Rhizoma, Coix Lacryma-jobi Semen→Poria Cocos* | 0.09 | 1 | 0.09 | 2.21 | 9 |
| *Hedyotis Diffusa Herba, Atractylodes Macrocephalae Rhizoma, Codonopsis Pilosulae Radix, Astragalus Membranacei Radix→Poria Cocos* | 0.12 | 1 | 0.12 | 2.21 | 12 |
| *Hedyotis Diffusa Herba, Atractylodes Macrocephalae Rhizoma, Codonopsis Pilosulae Radix, Astragalus Membranacei Radix, Coix Lacryma-jobi Semen→Poria Cocos* | 0.09 | 1 | 0.09 | 2.21 | 9 |
| *Atractylodes Macrocephalae Rhizoma, Pinellia Ternata Rhizoma, Poria Cocos→Glycyrrhiza Uralensis Radix Et Rhizoma* | 0.1 | 0.83 | 0.12 | 2.11 | 10 |
| *Pinellia Ternata Rhizoma, Poria Cocos, Astragalus Membranacei Radix→Glycyrrhiza Uralensis Radix Et Rhizoma* | 0.09 | 0.82 | 0.11 | 2.08 | 9 |
| *Hedyotis Diffusa Herba, Atractylodes Macrocephalae Rhizoma, Codonopsis Pilosulae Radix→Poria Cocos* | 0.14 | 0.94 | 0.15 | 2.07 | 15 |
| *Atractylodes Macrocephalae Rhizoma, Codonopsis Pilosulae Radix, Glycyrrhiza Uralensis Radix Et Rhizoma→Poria Cocos* | 0.14 | 0.94 | 0.15 | 2.07 | 15 |
| *Hedyotis Diffusa Herba, Codonopsis Pilosulae Radix, Astragalus Membranacei Radix→Poria Cocos* | 0.12 | 0.92 | 0.13 | 2.04 | 12 |
| *Pinellia Ternata Rhizoma, Glycyrrhiza Uralensis Radix Et Rhizoma→Poria Cocos* | 0.11 | 0.92 | 0.12 | 2.03 | 11 |
| *Atractylodes Macrocephalae Rhizoma, Codonopsis Pilosulae Radix, Glycyrrhiza Uralensis Radix Et Rhizoma, Astragalus Membranacei Radix→Poria Cocos* | 0.11 | 0.92 | 0.12 | 2.03 | 11 |
| *Atractylodes Macrocephalae Rhizoma, Actinidiae Radix→Poria Cocos* | 0.1 | 0.91 | 0.11 | 2.01 | 10 |
| *Hedyotis Diffusa Herba, Codonopsis Pilosulae Radix, Coix Lacryma-jobi Semen→Poria Cocos* | 0.1 | 0.91 | 0.11 | 2.01 | 10 |
| *Hedyotis Diffusa Herba, Atractylodes Macrocephalae Rhizoma, Codonopsis Pilosulae Radix, Coix Lacryma-jobi Semen→Poria Cocos* | 0.1 | 0.91 | 0.11 | 2.01 | 10 |
| *Atractylodes Macrocephalae Rhizoma, Codonopsis Pilosulae Radix, Curcumae Rhizoma→Poria Cocos* | 0.09 | 0.9 | 0.1 | 1.99 | 9 |
| *Codonopsis Pilosulae Radix, Glycyrrhiza Uralensis Radix Et Rhizoma, Coix Lacryma-jobi Semen→Poria Cocos* | 0.09 | 0.9 | 0.1 | 1.99 | 9 |
| *Hedyotis Diffusa Herba, Codonopsis Pilosulae Radix→Poria Cocos* | 0.14 | 0.88 | 0.16 | 1.95 | 15 |
| *Hedyotis Diffusa Herba, Atractylodes Macrocephalae Rhizoma, Glycyrrhiza Uralensis Radix Et Rhizoma→Poria Cocos* | 0.12 | 0.86 | 0.13 | 1.9 | 12 |
| *Atractylodes Macrocephalae Rhizoma, Glycyrrhiza Uralensis Radix Et Rhizoma, Coix Lacryma-jobi Semen→Poria Cocos* | 0.11 | 0.85 | 0.13 | 1.87 | 11 |
| *Codonopsis Pilosulae Radix, Glycyrrhiza Uralensis Radix Et Rhizoma, Astragalus Membranacei Radix→Poria Cocos* | 0.11 | 0.85 | 0.13 | 1.87 | 11 |
| *Hedyotis Diffusa Herba, Atractylodes Macrocephalae Rhizoma, Glycyrrhiza Uralensis Radix Et Rhizoma, Astragalus Membranacei Radix→Poria Cocos* | 0.1 | 0.83 | 0.12 | 1.84 | 10 |
| *Atractylodes Macrocephalae Rhizoma, Codonopsis Pilosulae Radix, Astragalus Membranacei Radix→Poria Cocos* | 0.17 | 0.82 | 0.21 | 1.81 | 18 |
| *Hedyotis Diffusa Herba, Glycyrrhiza Uralensis Radix Et Rhizoma→Poria Cocos* | 0.13 | 0.81 | 0.15 | 1.8 | 13 |
| *Atractylodes Macrocephalae Rhizoma, Pinellia Ternata Rhizoma→Poria Cocos* | 0.12 | 0.8 | 0.14 | 1.77 | 12 |
| *Atractylodes Macrocephalae Rhizoma, Scutellaria Barbata Herba→Poria Cocos* | 0.12 | 0.8 | 0.14 | 1.77 | 12 |
| *Glycyrrhiza Uralensis Radix Et Rhizoma, Coix Lacryma-jobi Semen→Poria Cocos* | 0.12 | 0.8 | 0.14 | 1.77 | 12 |
| *Atractylodes Macrocephalae Rhizoma, Glycyrrhiza Uralensis Radix Et Rhizoma→Poria Cocos* | 0.23 | 0.8 | 0.29 | 1.77 | 24 |
| *Poria Cocos, Actinidiae Radix→Atractylodes Macrocephalae Rhizoma* | 0.1 | 1 | 0.1 | 1.76 | 10 |
| *Codonopsis Pilosulae Radix, Poria Cocos→Atractylodes Macrocephalae Rhizoma* | 0.23 | 1 | 0.23 | 1.76 | 24 |
| *Pericarpium Citri Reticulatae Viride Pericarpium, Codonopsis Pilosulae Radix, Astragalus Membranacei Radix→Atractylodes Macrocephalae Rhizoma* | 0.09 | 1 | 0.09 | 1.76 | 9 |
| *Codonopsis Pilosulae Radix, Curcumae Rhizoma, Poria Cocos→Atractylodes Macrocephalae Rhizoma* | 0.09 | 1 | 0.09 | 1.76 | 9 |
| *Hedyotis Diffusa Herba, Codonopsis Pilosulae Radix, Coix Lacryma-jobi Semen→Atractylodes Macrocephalae Rhizoma* | 0.11 | 1 | 0.11 | 1.76 | 11 |
| *Codonopsis Pilosulae Radix, Poria Cocos, Coix Lacryma-jobi Semen→Atractylodes Macrocephalae Rhizoma* | 0.13 | 1 | 0.13 | 1.76 | 13 |
| *Hedyotis Diffusa Herba, Glycyrrhiza Uralensis Radix Et Rhizoma, Coix Lacryma-jobi Semen→Atractylodes Macrocephalae Rhizoma* | 0.09 | 1 | 0.09 | 1.76 | 9 |
| *Hedyotis Diffusa Herba, Codonopsis Pilosulae Radix, Poria Cocos→Atractylodes Macrocephalae Rhizoma* | 0.14 | 1 | 0.14 | 1.76 | 15 |
| *Codonopsis Pilosulae Radix, Poria Cocos, Glycyrrhiza Uralensis Radix Et Rhizoma→Atractylodes Macrocephalae Rhizoma* | 0.14 | 1 | 0.14 | 1.76 | 15 |
| *Codonopsis Pilosulae Radix, Poria Cocos, Astragalus Membranacei Radix→Atractylodes Macrocephalae Rhizoma* | 0.17 | 1 | 0.17 | 1.76 | 18 |
| *Hedyotis Diffusa Herba, Codonopsis Pilosulae Radix, Poria Cocos, Coix Lacryma-jobi Semen→Atractylodes Macrocephalae Rhizoma* | 0.1 | 1 | 0.1 | 1.76 | 10 |
| *Hedyotis Diffusa Herba, Codonopsis Pilosulae Radix, Astragalus Membranacei Radix, Coix Lacryma-jobi Semen→Atractylodes Macrocephalae Rhizoma* | 0.09 | 1 | 0.09 | 1.76 | 9 |
| *Codonopsis Pilosulae Radix, Poria Cocos, Glycyrrhiza Uralensis Radix Et Rhizoma, Coix Lacryma-jobi Semen→Atractylodes Macrocephalae Rhizoma* | 0.09 | 1 | 0.09 | 1.76 | 9 |
| *Codonopsis Pilosulae Radix, Poria Cocos, Astragalus Membranacei Radix, Coix Lacryma-jobi Semen→Atractylodes Macrocephalae Rhizoma* | 0.1 | 1 | 0.1 | 1.76 | 10 |
| *Hedyotis Diffusa Herba, Codonopsis Pilosulae Radix, Poria Cocos, Astragalus Membranacei Radix→Atractylodes Macrocephalae Rhizoma* | 0.12 | 1 | 0.12 | 1.76 | 12 |
| *Codonopsis Pilosulae Radix, Poria Cocos, Glycyrrhiza Uralensis Radix Et Rhizoma, Astragalus Membranacei Radix→Atractylodes Macrocephalae Rhizoma* | 0.11 | 1 | 0.11 | 1.76 | 11 |
| *Hedyotis Diffusa Herba, Codonopsis Pilosulae Radix, Poria Cocos, Astragalus Membranacei Radix, Coix Lacryma-jobi Semen→Atractylodes Macrocephalae Rhizoma* | 0.09 | 1 | 0.09 | 1.76 | 9 |
| *Hedyotis Diffusa Herba, Coix Lacryma-jobi Semen→Atractylodes Macrocephalae Rhizoma* | 0.17 | 0.95 | 0.18 | 1.67 | 18 |
| *Hedyotis Diffusa Herba, Codonopsis Pilosulae Radix→Atractylodes Macrocephalae Rhizoma* | 0.15 | 0.94 | 0.16 | 1.66 | 16 |
| *Hedyotis Diffusa Herba, Astragalus Membranacei Radix, Coix Lacryma-jobi Semen→Atractylodes Macrocephalae Rhizoma* | 0.15 | 0.94 | 0.16 | 1.66 | 16 |
| *Pericarpium Citri Reticulatae Viride Pericarpium, Astragalus Membranacei Radix→Atractylodes Macrocephalae Rhizoma* | 0.13 | 0.93 | 0.14 | 1.65 | 14 |
| *Hedyotis Diffusa Herba, Scutellaria Barbata Herba→Atractylodes Macrocephalae Rhizoma* | 0.13 | 0.93 | 0.13 | 1.64 | 13 |
| *Codonopsis Pilosulae Radix, Astragalus Membranacei Radix, Coix Lacryma-jobi Semen→Atractylodes Macrocephalae Rhizoma* | 0.13 | 0.93 | 0.13 | 1.64 | 13 |
| *Hedyotis Diffusa Herba, Poria Cocos, Coix Lacryma-jobi Semen→Atractylodes Macrocephalae Rhizoma* | 0.13 | 0.93 | 0.13 | 1.64 | 13 |
| *Curcumae Rhizoma, Poria Cocos→Atractylodes Macrocephalae Rhizoma* | 0.12 | 0.92 | 0.13 | 1.63 | 12 |
| *Poria Cocos, Glycyrrhiza Uralensis Radix Et Rhizoma→Atractylodes Macrocephalae Rhizoma* | 0.23 | 0.92 | 0.25 | 1.63 | 24 |
| *Hedyotis Diffusa Herba, Codonopsis Pilosulae Radix, Astragalus Membranacei Radix→Atractylodes Macrocephalae Rhizoma* | 0.12 | 0.92 | 0.13 | 1.63 | 12 |
| *Codonopsis Pilosulae Radix, Glycyrrhiza Uralensis Radix Et Rhizoma, Astragalus Membranacei Radix→Atractylodes Macrocephalae Rhizoma* | 0.12 | 0.92 | 0.13 | 1.63 | 12 |
| *Hedyotis Diffusa Herba, Poria Cocos, Glycyrrhiza Uralensis Radix Et Rhizoma→Atractylodes Macrocephalae Rhizoma* | 0.12 | 0.92 | 0.13 | 1.63 | 12 |
| *Hedyotis Diffusa Herba, Poria Cocos, Astragalus Membranacei Radix, Coix Lacryma-jobi Semen→Atractylodes Macrocephalae Rhizoma* | 0.12 | 0.92 | 0.13 | 1.63 | 12 |
| *Pericarpium Citri Reticulatae Viride Pericarpium, Poria Cocos→Atractylodes Macrocephalae Rhizoma* | 0.11 | 0.92 | 0.12 | 1.62 | 11 |
| *Codonopsis Pilosulae Radix, Astragalus Membranacei Radix→Atractylodes Macrocephalae Rhizoma* | 0.21 | 0.92 | 0.23 | 1.62 | 22 |
| *Hedyotis Diffusa Herba, Poria Cocos→Atractylodes Macrocephalae Rhizoma* | 0.21 | 0.92 | 0.23 | 1.62 | 22 |
| *Hedyotis Diffusa Herba, Scutellaria Barbata Herba, Astragalus Membranacei Radix→Atractylodes Macrocephalae Rhizoma* | 0.11 | 0.92 | 0.12 | 1.62 | 11 |
| *Poria Cocos, Glycyrrhiza Uralensis Radix Et Rhizoma, Coix Lacryma-jobi Semen→Atractylodes Macrocephalae Rhizoma* | 0.11 | 0.92 | 0.12 | 1.62 | 11 |
| *Pericarpium Citri Reticulatae Viride Pericarpium, Codonopsis Pilosulae Radix→Atractylodes Macrocephalae Rhizoma* | 0.1 | 0.91 | 0.11 | 1.6 | 10 |
| *Pinellia Ternata Rhizoma, Poria Cocos, Glycyrrhiza Uralensis Radix Et Rhizoma→Atractylodes Macrocephalae Rhizoma* | 0.1 | 0.91 | 0.11 | 1.6 | 10 |
| *Pinellia Ternata Rhizoma, Poria Cocos, Astragalus Membranacei Radix→Atractylodes Macrocephalae Rhizoma* | 0.1 | 0.91 | 0.11 | 1.6 | 10 |
| *Hedyotis Diffusa Herba, Scutellaria Barbata Herba, Poria Cocos→Atractylodes Macrocephalae Rhizoma* | 0.1 | 0.91 | 0.11 | 1.6 | 10 |
| *Hedyotis Diffusa Herba, Poria Cocos, Glycyrrhiza Uralensis Radix Et Rhizoma, Astragalus Membranacei Radix→Atractylodes Macrocephalae Rhizoma* | 0.1 | 0.91 | 0.11 | 1.6 | 10 |
| *Actinidiae Radix, Coix Lacryma-jobi Semen→Atractylodes Macrocephalae Rhizoma* | 0.09 | 0.9 | 0.1 | 1.59 | 9 |
| *Astragalus Membranacei Radix, Actinidiae Radix→Atractylodes Macrocephalae Rhizoma* | 0.09 | 0.9 | 0.1 | 1.59 | 9 |
| *Pericarpium Citri Reticulatae Viride Pericarpium, Coix Lacryma-jobi Semen→Atractylodes Macrocephalae Rhizoma* | 0.09 | 0.9 | 0.1 | 1.59 | 9 |
| *Codonopsis Pilosulae Radix, Coix Lacryma-jobi Semen→Atractylodes Macrocephalae Rhizoma* | 0.17 | 0.9 | 0.19 | 1.59 | 18 |
| *Poria Cocos, Coix Lacryma-jobi Semen→Atractylodes Macrocephalae Rhizoma* | 0.17 | 0.9 | 0.19 | 1.59 | 18 |
| *Pericarpium Citri Reticulatae Viride Pericarpium, Poria Cocos, Astragalus Membranacei Radix→Atractylodes Macrocephalae Rhizoma* | 0.09 | 0.9 | 0.1 | 1.59 | 9 |
| *Codonopsis Pilosulae Radix, Glycyrrhiza Uralensis Radix Et Rhizoma, Coix Lacryma-jobi Semen→Atractylodes Macrocephalae Rhizoma* | 0.09 | 0.9 | 0.1 | 1.59 | 9 |
| *Glycyrrhiza Uralensis Radix Et Rhizoma, Astragalus Membranacei Radix, Coix Lacryma-jobi Semen→Atractylodes Macrocephalae Rhizoma* | 0.09 | 0.9 | 0.1 | 1.59 | 9 |
| *Hedyotis Diffusa Herba, Poria Cocos, Astragalus Membranacei Radix→Atractylodes Macrocephalae Rhizoma* | 0.17 | 0.9 | 0.19 | 1.59 | 18 |
| *Hedyotis Diffusa Herba, Poria Cocos, Coix Lacryma-jobi Semen→Astragalus Membranacei Radix* | 0.13 | 0.93 | 0.13 | 1.58 | 13 |
| *Poria Cocos, Glycyrrhiza Uralensis Radix Et Rhizoma, Astragalus Membranacei Radix→Atractylodes Macrocephalae Rhizoma* | 0.16 | 0.89 | 0.18 | 1.58 | 17 |
| *Hedyotis Diffusa Herba, Atractylodes Macrocephalae Rhizoma, Poria Cocos, Coix Lacryma-jobi Semen→Astragalus Membranacei Radix* | 0.12 | 0.92 | 0.13 | 1.57 | 12 |
| *Hedyotis Diffusa Herba, Glycyrrhiza Uralensis Radix Et Rhizoma→Atractylodes Macrocephalae Rhizoma* | 0.13 | 0.88 | 0.15 | 1.54 | 14 |
| *Pinellia Ternata Rhizoma, Coix Lacryma-jobi Semen→Astragalus Membranacei Radix* | 0.09 | 0.9 | 0.1 | 1.53 | 9 |
| *Scutellaria Barbata Herba, Coix Lacryma-jobi Semen→Astragalus Membranacei Radix* | 0.09 | 0.9 | 0.1 | 1.53 | 9 |
| *Atractylodes Macrocephalae Rhizoma, Pericarpium Citri Reticulatae Viride Pericarpium, Codonopsis Pilosulae Radix→Astragalus Membranacei Radix* | 0.09 | 0.9 | 0.1 | 1.53 | 9 |
| *Hedyotis Diffusa Herba, Codonopsis Pilosulae Radix, Poria Cocos, Coix Lacryma-jobi Semen→Astragalus Membranacei Radix* | 0.09 | 0.9 | 0.1 | 1.53 | 9 |
| *Hedyotis Diffusa Herba, Atractylodes Macrocephalae Rhizoma, Codonopsis Pilosulae Radix, Poria Cocos, Coix Lacryma-jobi Semen→Astragalus Membranacei Radix* | 0.09 | 0.9 | 0.1 | 1.53 | 9 |
| *Pinellia Ternata Rhizoma, Astragalus Membranacei Radix→Atractylodes Macrocephalae Rhizoma* | 0.13 | 0.87 | 0.14 | 1.53 | 13 |
| *Glycyrrhiza Uralensis Radix Et Rhizoma, Coix Lacryma-jobi Semen→Atractylodes Macrocephalae Rhizoma* | 0.13 | 0.87 | 0.14 | 1.53 | 13 |
| *Poria Cocos, Astragalus Membranacei Radix, Coix Lacryma-jobi Semen→Atractylodes Macrocephalae Rhizoma* | 0.13 | 0.87 | 0.14 | 1.53 | 13 |
| *Hedyotis Diffusa Herba, Coix Lacryma-jobi Semen→Astragalus Membranacei Radix* | 0.16 | 0.89 | 0.18 | 1.53 | 17 |
| *Codonopsis Pilosulae Radix→Atractylodes Macrocephalae Rhizoma* | 0.3 | 0.86 | 0.35 | 1.52 | 31 |
| *Hedyotis Diffusa Herba, Atractylodes Macrocephalae Rhizoma, Coix Lacryma-jobi Semen→Astragalus Membranacei Radix* | 0.15 | 0.89 | 0.17 | 1.52 | 16 |
| *Scutellaria Barbata Herba, Poria Cocos→Atractylodes Macrocephalae Rhizoma* | 0.12 | 0.86 | 0.13 | 1.51 | 12 |
| *Hedyotis Diffusa Herba, Astragalus Membranacei Radix→Atractylodes Macrocephalae Rhizoma* | 0.23 | 0.86 | 0.27 | 1.51 | 24 |
| *Hedyotis Diffusa Herba, Glycyrrhiza Uralensis Radix Et Rhizoma, Astragalus Membranacei Radix→Atractylodes Macrocephalae Rhizoma* | 0.12 | 0.86 | 0.13 | 1.51 | 12 |
| *Hedyotis Diffusa Herba→Atractylodes Macrocephalae Rhizoma* | 0.28 | 0.85 | 0.33 | 1.5 | 29 |
| *Atractylodes Macrocephalae Rhizoma, Pericarpium Citri Reticulatae Viride Pericarpium→Astragalus Membranacei Radix* | 0.13 | 0.88 | 0.15 | 1.49 | 14 |
| *Hedyotis Diffusa Herba, Glycyrrhiza Uralensis Radix Et Rhizoma→Astragalus Membranacei Radix* | 0.13 | 0.88 | 0.15 | 1.49 | 14 |
| *Actinidiae Radix→Atractylodes Macrocephalae Rhizoma* | 0.11 | 0.85 | 0.13 | 1.49 | 11 |
| *Codonopsis Pilosulae Radix, Glycyrrhiza Uralensis Radix Et Rhizoma→Atractylodes Macrocephalae Rhizoma* | 0.15 | 0.84 | 0.18 | 1.48 | 16 |
| *Astragalus Membranacei Radix, Coix Lacryma-jobi Semen→Atractylodes Macrocephalae Rhizoma* | 0.2 | 0.84 | 0.24 | 1.48 | 21 |
| *Atractylodes Macrocephalae Rhizoma, Pinellia Ternata Rhizoma→Astragalus Membranacei Radix* | 0.13 | 0.87 | 0.14 | 1.48 | 13 |
| *Pinellia Ternata Rhizoma, Glycyrrhiza Uralensis Radix Et Rhizoma→Atractylodes Macrocephalae Rhizoma* | 0.1 | 0.83 | 0.12 | 1.47 | 10 |
| *Pericarpium Citri Reticulatae Viride Pericarpium, Glycyrrhiza Uralensis Radix Et Rhizoma→Atractylodes Macrocephalae Rhizoma* | 0.1 | 0.83 | 0.12 | 1.47 | 10 |
| *Poria Cocos→Atractylodes Macrocephalae Rhizoma* | 0.38 | 0.83 | 0.45 | 1.46 | 39 |
| *Hedyotis Diffusa Herba, Scutellaria Barbata Herba→Astragalus Membranacei Radix* | 0.12 | 0.86 | 0.13 | 1.46 | 12 |
| *Hedyotis Diffusa Herba, Atractylodes Macrocephalae Rhizoma, Glycyrrhiza Uralensis Radix Et Rhizoma→Astragalus Membranacei Radix* | 0.12 | 0.86 | 0.13 | 1.46 | 12 |
| *Coix Lacryma-jobi Semen→Atractylodes Macrocephalae Rhizoma* | 0.27 | 0.82 | 0.33 | 1.45 | 28 |
| *Poria Cocos, Astragalus Membranacei Radix→Atractylodes Macrocephalae Rhizoma* | 0.27 | 0.82 | 0.33 | 1.45 | 28 |
| *Glycyrrhiza Uralensis Radix Et Rhizoma, Astragalus Membranacei Radix→Atractylodes Macrocephalae Rhizoma* | 0.22 | 0.82 | 0.27 | 1.45 | 23 |
| *Hedyotis Diffusa Herba, Atractylodes Macrocephalae Rhizoma, Scutellaria Barbata Herba→Astragalus Membranacei Radix* | 0.11 | 0.85 | 0.13 | 1.44 | 11 |
| *Hedyotis Diffusa Herba, Poria Cocos, Glycyrrhiza Uralensis Radix Et Rhizoma→Astragalus Membranacei Radix* | 0.11 | 0.85 | 0.13 | 1.44 | 11 |
| *Scutellaria Barbata Herba→Astragalus Membranacei Radix* | 0.15 | 0.84 | 0.18 | 1.44 | 16 |
| *Pericarpium Citri Reticulatae Viride Pericarpium, Poria Cocos→Astragalus Membranacei Radix* | 0.1 | 0.83 | 0.12 | 1.42 | 10 |
| *Hedyotis Diffusa Herba, Poria Cocos→Astragalus Membranacei Radix* | 0.19 | 0.83 | 0.23 | 1.42 | 20 |
| *Atractylodes Macrocephalae Rhizoma, Pinellia Ternata Rhizoma, Poria Cocos→Astragalus Membranacei Radix* | 0.1 | 0.83 | 0.12 | 1.42 | 10 |
| *Hedyotis Diffusa Herba, Atractylodes Macrocephalae Rhizoma, Poria Cocos, Glycyrrhiza Uralensis Radix Et Rhizoma→Astragalus Membranacei Radix* | 0.1 | 0.83 | 0.12 | 1.42 | 10 |
| *Hedyotis Diffusa Herba, Atractylodes Macrocephalae Rhizoma→Astragalus Membranacei Radix* | 0.23 | 0.83 | 0.28 | 1.41 | 24 |
| *Pericarpium Citri Reticulatae Viride Pericarpium→Atractylodes Macrocephalae Rhizoma* | 0.15 | 0.8 | 0.19 | 1.41 | 16 |
| *Pinellia Ternata Rhizoma, Poria Cocos→Atractylodes Macrocephalae Rhizoma* | 0.12 | 0.8 | 0.14 | 1.41 | 12 |
| *Hedyotis Diffusa Herba→Astragalus Membranacei Radix* | 0.27 | 0.82 | 0.33 | 1.4 | 28 |
| *Atractylodes Macrocephalae Rhizoma, Actinidiae Radix→Astragalus Membranacei Radix* | 0.09 | 0.82 | 0.11 | 1.39 | 9 |
| *Pericarpium Citri Reticulatae Viride Pericarpium, Codonopsis Pilosulae Radix→Astragalus Membranacei Radix* | 0.09 | 0.82 | 0.11 | 1.39 | 9 |
| *Pinellia Ternata Rhizoma, Poria Cocos, Glycyrrhiza Uralensis Radix Et Rhizoma→Astragalus Membranacei Radix* | 0.09 | 0.82 | 0.11 | 1.39 | 9 |
| *Hedyotis Diffusa Herba, Scutellaria Barbata Herba, Poria Cocos→Astragalus Membranacei Radix* | 0.09 | 0.82 | 0.11 | 1.39 | 9 |
| *Atractylodes Macrocephalae Rhizoma, Pericarpium Citri Reticulatae Viride Pericarpium, Poria Cocos→Astragalus Membranacei Radix* | 0.09 | 0.82 | 0.11 | 1.39 | 9 |
| *Atractylodes Macrocephalae Rhizoma, Curcumae Rhizoma, Coix Lacryma-jobi Semen→Astragalus Membranacei Radix* | 0.09 | 0.82 | 0.11 | 1.39 | 9 |
| *Hedyotis Diffusa Herba, Codonopsis Pilosulae Radix, Coix Lacryma-jobi Semen→Astragalus Membranacei Radix* | 0.09 | 0.82 | 0.11 | 1.39 | 9 |
| *Hedyotis Diffusa Herba, Atractylodes Macrocephalae Rhizoma, Poria Cocos→Astragalus Membranacei Radix* | 0.17 | 0.82 | 0.21 | 1.39 | 18 |
| *Hedyotis Diffusa Herba, Atractylodes Macrocephalae Rhizoma, Codonopsis Pilosulae Radix, Coix Lacryma-jobi Semen→Astragalus Membranacei Radix* | 0.09 | 0.82 | 0.11 | 1.39 | 9 |
| *Atractylodes Macrocephalae Rhizoma, Scutellaria Barbata Herba→Astragalus Membranacei Radix* | 0.12 | 0.8 | 0.14 | 1.36 | 12 |
| *Hedyotis Diffusa Herba, Codonopsis Pilosulae Radix, Poria Cocos→Astragalus Membranacei Radix* | 0.12 | 0.8 | 0.14 | 1.36 | 12 |
| *Hedyotis Diffusa Herba, Atractylodes Macrocephalae Rhizoma, Codonopsis Pilosulae Radix, Poria Cocos→Astragalus Membranacei Radix* | 0.12 | 0.8 | 0.14 | 1.36 | 12 |
| *Angelica Sinensis Radix, Glycyrrhiza Uralensis Radix Et Rhizoma→Atractylodes Macrocephalae Rhizoma* | 0.09 | 0.82 | 0.11 | 1.44 | 9 |
| *Curcumae Rhizoma, Glycyrrhiza Uralensis Radix Et Rhizoma→Atractylodes Macrocephalae Rhizoma* | 0.09 | 0.82 | 0.11 | 1.44 | 9 |
| *Scutellaria Barbata Herba, Poria Cocos, Astragalus Membranacei Radix→Atractylodes Macrocephalae Rhizoma* | 0.09 | 0.82 | 0.11 | 1.44 | 9 |
| *Curcumae Rhizoma, Astragalus Membranacei Radix, Coix Lacryma-jobi Semen→Atractylodes Macrocephalae Rhizoma* | 0.09 | 0.82 | 0.11 | 1.44 | 9 |

**STable.3. General treatment prescriptions for CRC from RCTs, marketed Chinese patent drug and TCM combinations obtained a patent*.**

| **Number** | Identification number* | **Herb** |
| --- | --- | --- |
| 1 | ITMCTR1900002714 | *Astragalus Membranacei Radix, Cuscutae Semen, Codonopsis Pilosulae Radix, Polygonatum Sibiricum Rhizoma, Lycium Barbarum Fructus, Ligustrum Lucidum Fructus, Epimedium Brevicornu Herba, Spatholobus Suberectus Caulis, Citrus Medica Var. Sarcodactylis Fructus, Prunus Mume Flos* |
| 2 | ITMCTR1900002635 | *Coptis Chinensis Rhizoma, Euodia Rutaecarpa Fructus, Aucklandia Lappa Radix* |
| 3 | ITMCTR2200005722 | *Bupleurum Chinense Radix, Scutellaria Baicalensis Radix, Rheum Palmatum Radix Et Rhizoma, Citrus Aurantium Fructus Immaturus, Pinellia Ternata Rhizoma, Paeonia Lactiflora Radix Alba, Ziziphus Jujuba Fructus, Zingiber Officinale Rhizoma Recens* |
| 4 | ITMCTR2200005671 | *Paeonia Suffruticosa Cortex, Ligusticum Chuanxiong Rhizoma Rhizoma, Carthamus Tinctorius Flos* |
| 5 | ChiCTR1800014718 | *Cinnamomum Cassia Ramulus, Cinnamomum Cassia Cortex, Aconitum Carmichaelii Radix Praeparata, Epimedium Brevicornu Herba, Cynomorii Herba, Zingiber Officinale Rhizoma, Agrimoniae Herba, Pseudostellariae Radix, Atractylodes Macrocephalae Rhizoma, Poria Cocos* |
| 6 | ChiCTR1900025051 | *Atractylodes Macrocephalae Rhizoma, Paeonia Lactiflora Radix Alba, Saposhnikovia Divaricata Radix, Pericarpium Citri Reticulatae Viride, Mume Fructus, Glycyrrhiza Uralensis Radix Et Rhizoma* |
| 7 | ChiCTR2000032996 | *Astragalus Membranacei Radix, Cinnamomum Cassia Ramulus, Paeoniae Radix Rubra, Ligusticum Chuanxiong Rhizoma Rhizoma, Angelica Sinensis Radix, Salvia Miltiorrhiza Radix Et Rhizoma, Clematidis Radix et Rhizoma, Cynanchi Paniculati Radix et Rhizoma* |
| 8 | ChiCTR2100049792 | *Phellinus Igniarius* |
| 9 | ChiCTR2100051831 | *Testudinis Plastrum, Trionycis Carapax, Cornu Cervi Colla, Codonopsis Pilosulae Radix, Lycium Barbarum Fructus, Polygonatum Sibiricum Rhizoma, Ligustrum Lucidum Fructus, Ecliptae Herba, Pericarpium Citri Reticulatae Viride, Galli Gigerii Endothelium Corneum, Hordei Fructus Germinatus, Crataegi Fructus, Cyperi Rhizoma, Asini Corii Colla, Polygonati Odorati Rhizoma, Ophiopogonis Radix* |
| 10 | ChiCTR2100054728 | *Astragalus Membranacei Radix, Cinnamomum Cassia Ramulus, Paeonia Lactiflora Radix Alba, Zingiber Officinale Rhizoma Recens, Ziziphus Jujuba Fructus* |
| 11 | ChiCTR2200056963 | *Astragalus Membranacei Radix, Angelica Sinensis Radix* |
| 12 | ChiCTR2200063670 | *Corydalis Decumbentis Rhizoma, Aconiti Radix, Venenum Bufonis, Daphnes Cortex, Chelidonii Herba, Gentianae Macrophyllae Radix, Angelica Dahurica Radix, Ligusticum Chuanxiong Rhizoma Rhizoma, Paeonia Lactiflora Radix Alba, Glycyrrhiza Uralensis Radix Et Rhizoma* |
| 13 | ChiCTR2300074230 | *Astragalus Membranacei Radix, Pini Nodi Lignum, Cornu Bovis, Agrimoniae Herba, Spatholobus Suberectus Caulis* |
| 14 | ChiCTR-TRC-08000130 | *Ginseng Radix et Rhizoma Rubra, Aconitum Carmichaelii Radix Praeparata* |
| 15 | NCT06394128 | *Panax Ginseng Radix Et Rhizoma, Atractylodes Macrocephalae Rhizoma, Poria Cocos, Glycyrrhiza Uralensis Radix Et Rhizoma, Pinellia Ternata Rhizoma, Pericarpium Citri Reticulatae Viride, Atractylodes Lancea Rhizoma, Cyperi Rhizoma, Scutellaria Baicalensis Radix, Ligusticum Chuanxiong Rhizoma Rhizoma, Angelica Sinensis Radix, Aurantii Fructus* |
| 16 | NCT04690283 | *Astragalus Membranacei Radix, Cinnamomum Cassia Ramulus, Paeonia Lactiflora Radix Alba, Zingiber Officinale Rhizoma Recens, Ziziphus Jujuba Fructus* |
| 17 | Z20060303 | *Astragalus Membranacei Radix, Ligustrum Lucidum Fructus* |
| 18 | CN112121091A | *Mume Fructus, Bombyx Batryticatus, Sophorae Flos* |
| 19 | CN110090282A | *Pseudostellariae Radix, Poria Cocos, Atractylodes Macrocephalae Rhizoma, Glycyrrhiza Uralensis Radix Et Rhizoma, Aucklandia Lappa Radix, Amomum Villosum Fructus, Pinellia Ternata Rhizoma, Pericarpium Citri Reticulatae Viride, Galli Gigerii Endothelium Corneum, Massa Medicata Fermentata, Hordei Fructus Germinatus, Crataegi Fructus, Setariae Fructus Germinatus* |
| 20 | CN107875210A | *Mume Fructus, Bombyx Batryticatus* |
| 21 | CN108310165A | *Astragalus Membranacei Radix, Paeonia Lactiflora Radix Alba, Cyperi Rhizoma, Glycyrrhiza Uralensis Radix Et Rhizoma, Epimedium Brevicornu Herba, Berchemia lineata* |
| 22 | CN102824404A | *Panax Ginseng Radix Et Rhizoma, Sophora Flavescens Radix, Pelargonii Herba* |
| 23 | CN113713057A | *Poria Cocos, Dioscoreae Rhizoma, Coix Lacryma-jobi Semen, Actinidiae Radix, Duchesneae Indicae Herba, Sargentodoxae Caulis, Aucklandia Lappa Radix, Glycyrrhiza Uralensis Radix Et Rhizoma* |
| 24 | CN110833578A | *Rheum Palmatum Radix Et Rhizoma, Angelica Sinensis Radix, Codonopsis Pilosulae Radix, Cimicifugae Rhizoma, Aconitum Carmichaelii Radix Praeparata* |
| 25 | CN111700982A | *Taxus chinensis, Coix Lacryma-jobi Semen, Lonicera Japonica Flos, Phragmitis Caulis, Angelica Sinensis Radix, Trichosanthis Radix, Scrophulariae Radix, Angelica Dahurica Radix, Glycyrrhiza Uralensis Radix Et Rhizoma* |
| 26 | CN109512995A | *Caryophylli Flos, Amomum Villosum Fructus, Foeniculi Fructus, Cinnamomum Cassia Cortex, Piperis Albi Fructus, Aucklandia Lappa Radix, Zingiber Officinale Rhizoma* |
| 27 | CN116650551A | *Spatholobus Suberectus Caulis, Gentianae Macrophyllae Radix, Faeces Bombycis, Scolopendra* |
| 28 | CN117883536A | *Radix Actinidiae Valvatae, Codonopsis Pilosulae Radix, Portulacae Herba, Coix Lacryma-jobi Semen, Amomi Rotundus Fructus, Phellodendron Chinense Cortex, Sargentodoxae Caulis, Linderae Radix, Notopterygii Rhizoma et Radix, Mume Fructus, Halloysitum Rubrum* |
| 29 | CN115463201A | *Mume Fructus, Coptis Chinensis Rhizoma, Zingiber Officinale Rhizoma, Phellodendron Chinense Cortex, Angelica Sinensis Radix* |
| 30 | CN119607166A | *Panax Ginseng Radix Et Rhizoma, Glycyrrhiza Uralensis Radix Et Rhizoma, Atractylodes Macrocephalae Rhizoma, Ophiopogonis Radix, Zingiber Officinale Rhizoma, Inulae Flos, Schisandrae Chinensis Fructus* |
| 31 | CN114732884A | *Trichosanthis Cucumeroidis Radix, Ruta graveolens, Zanthoxyli Nitidi Radix, Ophiocordyceps Sinensis, Taraxacum Mongolicum Herba, Scutellaria Barbata Herba, Salvia Miltiorrhiza Radix Et Rhizoma, Curcumae Longae Rhizoma, Smilacis Glabrae Rhizoma, Schisandrae Chinensis Fructus, Bupleurum Chinense Radix, Ganoderma Lucidum, Panax Ginseng Radix Et Rhizoma, Trionycis Carapax* |
| 32 | CN113082110A | *Angelica Sinensis Radix, Rehmannia Glutinosa Radix Praeparata, Ligusticum Chuanxiong Rhizoma Rhizoma, Paeonia Lactiflora Radix Alba, Astragalus Membranacei Radix, Codonopsis Pilosulae Radix, Glycyrrhiza Uralensis Radix Et Rhizoma* |
| 33 | CN106668389A | *Citrus Aurantium Fructus Immaturus, Linderae Radix, Arecae Pericarpium, Aucklandia Lappa Radix, Phellodendron Chinense Cortex, Alismatis Rhizoma, Notoginseng Radix et Rhizoma, Achyranthis Bidentatae Radix, Panacis Quinquefolii Radix, Glycyrrhiza Uralensis Radix Et Rhizoma* |
| 34 | CN110141623A | *Pseudostellariae Radix, Poria Cocos, Atractylodes Macrocephalae Rhizoma, Glycyrrhiza Uralensis Radix Et Rhizoma, Pinellia Ternata Rhizoma, Pericarpium Citri Reticulatae Viride* |
| 35 | CN108498546A | *Gecko, Pheretima, Armadillidium, Vespae Pupa* |
| 36 | CN110025674A | *Astragalus Membranacei Radix, Angelica Sinensis Radix, Ecliptae Herba, Ligustrum Lucidum Fructus, Psoraleae Fructus, Cuscutae Semen* |
| 37 | CN108542941A | *Actinidiae Radix, Ranunculi Ternati Radix, Scutellaria Barbata Herba, Scolopendra* |
| 38 | CN114652750A | *Moschus Artifactus, Calculus Bovis, Margarita, Succinum, Borax, Borneolum, Calamina Preparata* |
| 39 | CN116942748A | *Pseudostellariae Radix, Poria Cocos, Atractylodes Macrocephalae Rhizoma, Glycyrrhiza Uralensis Radix Et Rhizoma, Pinellia Ternata Rhizoma, Pericarpium Citri Reticulatae Viride* |
| 40 | CN115154576A | *Psoraleae Fructus, Schisandrae Chinensis Fructus, Mume Fructus, Euryales Semen, Nelumbinis Semen, Zingiber Officinale Rhizoma, Granati Pericarpium, Angelica Dahurica Radix* |
| 41 | CN114984142A | *Atractylodes Macrocephalae Rhizoma, Angelica Dahurica Radix, Ampelopsis Radix, Bletillae Rhizoma, Poria Cocos, Aconitum Carmichaelii Radix Praeparata, Asari Radix et Rhizoma* |
| 42 | CN108159160A | *Puerariae Lobatae Radix, Scutellaria Baicalensis Radix, Coptis Chinensis Rhizoma, Glycyrrhiza Uralensis Radix Et Rhizoma, Paeonia Lactiflora Radix Alba, Atractylodes Macrocephalae Rhizoma, Saposhnikovia Divaricata Radix, Pericarpium Citri Reticulatae Viride, Ziziphus Jujuba Fructus* |
| 43 | CN107029084A | *Pinellia Ternata Rhizoma, Rheum Palmatum Radix Et Rhizoma, Arisaema Cum Bile, Paridis Rhizoma, Coptis Chinensis Rhizoma* |
| 44 | CN108295203A | *Patriniae Herba, Lonicera Japonica Flos, Violae Herba, Paeonia Suffruticosa Cortex, Salvia Miltiorrhiza Radix Et Rhizoma, Aconitum Carmichaelii Radix Praeparata, Coix Lacryma-jobi Semen, Gleditsiae Sinensis Fructus, Aucklandia Lappa Radix, Squama Manis* |
| 45 | CN113350438A | *Portulacae Herba, Zanthoxyli Pericarpium, Atractylodes Lancea Rhizoma, Saposhnikovia Divaricata Radix, Natrii Sulfas, Alumen, Forsythia Suspensa Fructus, Platycladi Cacumen, Allii Fistulosi Bulbus* |
| 46 | CN1618447A | *Rabdosiae Rubescentis Herba, Solani Nigri Herba, Duchesneae Indicae Herba, Visci Herba, Gynostemmatis Herba, Acanthopanacis Senticosi Radix et Rhizoma, Chebulae Fructus, Citri Fructus* |
| 47 | CN118717904A | *Lophatheri Herba, Ginseng Radix et Rhizoma, Ophiopogonis Radix, Pinellia Ternata Rhizoma, Glycyrrhiza Uralensis Radix Et Rhizoma, Epimedium Brevicornu Herba, Agrimoniae Herba, Arctii Fructus, Citrus Aurantium Fructus Immaturus, Atractylodes Macrocephalae Rhizoma* |
| 48 | CN102430055A | *Hominis Placenta, Carthamus Tinctorius Flos, Venenum Bufonis, Cremastrae Pseudobulbus, Scolopendra, Calculus Bovis, Ganoderma Lucidum, Galli Gigerii Endothelium Corneum, Pinellia Ternata Rhizoma, Natrii Sulfas Exsiccatus* |
| 49 | CN115531457A | *Notopterygii Rhizoma et Radix, Angelicae Pubescentis Radix, Poria Cocos, Saposhnikovia Divaricata Radix, Schizonepetae Herba, Ligusticum Chuanxiong Rhizoma Rhizoma, Platycodonis Radix, Bupleurum Chinense Radix, Peucedani Radix, Aurantii Fructus, Glycyrrhiza Uralensis Radix Et Rhizoma* |
| 50 | CN114848770A | *Astragalus Membranacei Radix, Codonopsis Pilosulae Radix, Angelica Sinensis Radix, Citrus Medica Var. Sarcodactylis Fructus, Poria Cocos, Alpiniae Oxyphyllae Fructus, Panacis Quinquefolii Radix, Pseudostellariae Radix, Schisandrae Chinensis Fructus, Lycium Barbarum Fructus, Chrysanthemi Flos* |
| 51 | CN109010495A | *Astilbes Herba, Phellodendron Chinense Cortex, Paeoniae Radix Rubra, Rehmanniae Radix, Lonicera Japonica Flos* |
| 52 | 10.3969/j.issn.1673-9701.2010.15.016 | *Kansui Radix, Natrii Sulfas et Rhei Radix et Rhizoma, Magnoliae Officinalis Cortex, Raphani Semen, Aurantii Fructus, Prunus Persica Semen, Paeoniae Radix Rubra* |
| 53 | 10.16715/j.cnki.1671-1246.2012.03.052 | *Codonopsis Pilosulae Radix, Atractylodes Macrocephalae Rhizoma, Aconitum Carmichaelii Radix Praeparata, Cinnamomum Cassia Ramulus, Cyperi Rhizoma, Rheum Palmatum Radix Et Rhizoma, Magnoliae Officinalis Cortex, Citrus Aurantium Fructus Immaturus, Paeoniae Radix Rubra, Prunus Persica Semen* |
| 54 | 10.15912/j.cnki.gmoj.2014.03.026 | *Epimedium Brevicornu Herba, Hominis Placenta, Astragalus Membranacei Radix, Rehmannia Glutinosa Radix Praeparata, Cinnamomum Cassia Cortex, Angelica Sinensis Radix, Spatholobus Suberectus Caulis, Ligustrum Lucidum Fructus, Lycium Barbarum Fructus, Poria Cocos, Pericarpium Citri Reticulatae Viride, Glycyrrhiza Uralensis Radix Et Rhizoma* |
| 55 | 10.16661/j.cnki.1673-2197.2015.09.028 | *Rheum Palmatum Radix Et Rhizoma, Raphani Semen, Magnoliae Officinalis Cortex, Citrus Aurantium Fructus Immaturus, Aucklandia Lappa Radix, Pericarpium Citri Reticulatae Viride, Pericarpium Citri Reticulatae Viride, Caryophylli Flos, Zingiberis Rhizoma Preparatum* |
| 56 | 10.13457/j.cnki.jncm.2016.06.090 | *Panax Ginseng Radix Et Rhizoma, Poria Cocos, Atractylodes Macrocephalae Rhizoma, Pinellia Ternata Rhizoma, Pericarpium Citri Reticulatae Viride, Aucklandia Lappa Radix, Glycyrrhiza Uralensis Radix Et Rhizoma, Amomum Villosum Fructus* |
| 57 | 10.16295/j.cnki.0257-358x.2016.12.008 | *Panax Ginseng Radix Et Rhizoma, Zingiber Officinale Rhizoma, Atractylodes Macrocephalae Rhizoma, Coptis Chinensis Rhizoma, Glycyrrhiza Uralensis Radix Et Rhizoma, Scutellaria Baicalensis Radix* |
| 58 | 10.16588/j.cnki.issn2095-7785.2018.01.010 | *Gypsum Fibrosum, Borax, Borneolum, Margarita, Coptis Chinensis Rhizoma, Realgar, Olibanum, Myrrha, Bletillae Rhizoma, Notoginseng Radix et Rhizoma, Schisandrae Chinensis Fructus, Astragalus Membranacei Radix* |
| 59 | 10.13457/j.cnki.jncm.2018.05.040 | *Panax Ginseng Radix Et Rhizoma, Rehmannia Glutinosa Radix Praeparata, Poria Cocos, Ligusticum Chuanxiong Rhizoma Rhizoma, Trogopterorum Faeces, Corydalis Rhizoma, Atractylodes Macrocephalae Rhizoma, Angelica Sinensis Radix, Myrrha, Paeonia Lactiflora Radix Alba, Glycyrrhiza Uralensis Radix Et Rhizoma* |
| 60 | 10.13424/j.cnki.mtcm.2019.02.020 | *Ephedrae Herba, Paeonia Lactiflora Radix Alba, Astragalus Membranacei Radix, Glycyrrhiza Uralensis Radix Et Rhizoma, Aconitum Carmichaeli, Angelica Sinensis Radix, Ligusticum Chuanxiong Rhizoma Rhizoma* |
| 61 | 10.13193/j.issn.1673-7717.2019.08.026 | *Astragalus Membranacei Radix, Cinnamomum Cassia Ramulus, Rehmannia Glutinosa Radix Praeparata, Paeoniae Radix Rubra, Angelica Sinensis Radix, Ligusticum Chuanxiong Rhizoma Rhizoma, Pheretima, Spatholobus Suberectus Caulis, Tribuli Fructus, Zingiber Officinale Rhizoma Recens, Ziziphus Jujuba Fructus* |
| 62 | 10.16532/j.cnki.1674-3849.2019.07.012 | *Astragalus Membranacei Radix, Cinnamomum Cassia Ramulus, Paeonia Lactiflora Radix Alba, Angelica Sinensis Radix, Ziziphus Jujuba Fructus, Zingiber Officinale Rhizoma Recens* |
| 63 | 10.13457/j.cnki.jncm.2020.17.007 | *Scutellaria Baicalensis Radix, Paeonia Lactiflora Radix Alba, Codonopsis Pilosulae Radix, Pinellia Ternata Rhizoma, Glycyrrhiza Uralensis Radix Et Rhizoma, Hordei Fructus Germinatus* |
| 64 | 10.16791/j.cnki.sgdc.2020.15.032 | *Magnoliae Officinalis Cortex, Rheum Palmatum Radix Et Rhizoma, Euodia Rutaecarpa Fructus, Cinnamomum Cassia Cortex* |
| 65 | 10.13457/j.cnki.jncm.2020.03.012 | *Glycyrrhiza Uralensis Radix Et Rhizoma, Aucklandia Lappa Radix, Natrii Sulfas, Angelica Sinensis Radix, Paeoniae Radix Rubra, Citrus Aurantium Fructus Immaturus, Rehmanniae Radix, Magnoliae Officinalis Cortex, Prunus Persica Semen, Astragalus Membranacei Radix, Raphani Semen, Rheum Palmatum Radix Et Rhizoma, Cannabis Semen* |
| 66 | 10.13862/j.cnki.cn43-1446/r.2021.02.012 | *Rehmannia Glutinosa Radix Praeparata, Paeonia Lactiflora Radix Alba, Angelica Sinensis Radix, Ligusticum Chuanxiong Rhizoma Rhizoma, Codonopsis Pilosulae Radix, Astragalus Membranacei Radix* |
| 67 | 10.16662/j.cnki.1674-0742.2022.13.010 | *Scorpio, Scolopendra, Eupolyphaga Seu Steleophaga, Pheretima, Dioscoreae Rhizoma, Atractylodes Macrocephalae Rhizoma* |
| 68 | 10.16367/j.cnki.1002-2619.2022.05.025 | *Scutellaria Baicalensis Radix, Portulacae Herba, Sophora Flavescens Radix, Dictamni Cortex* |
| 69 | 10.19811/j.cnki.ISSN2096-6628.2022.07.003 | *Atractylodes Macrocephalae Rhizoma, Poria Cocos, Dioscoreae Rhizoma, Pseudostellariae Radix, Glycyrrhiza Uralensis Radix Et Rhizoma, Hordei Fructus Germinatus, Magnoliae Officinalis Cortex, Amomum Villosum Fructus, Galli Gigerii Endothelium Corneum, Citrus Medica Var. Sarcodactylis Fructus, Aurantii Fructus* |
| 70 | 10.13863/j.issn1008-0805.2015.03.041 | *Pseudostellariae Radix, Atractylodes Macrocephalae Rhizoma, Poria Cocos, Dioscoreae Rhizoma, Coptis Chinensis Rhizoma, Aucklandia Lappa Radix, Citrus Aurantium Fructus Immaturus, Sanguisorbae Radix, Scutellaria Barbata Herba, Smilacis Glabrae Rhizoma, Solani Septemlobi Herba, Glycyrrhiza Uralensis Radix Et Rhizoma* |
| 71 | 10.13633/j.cnki.1000-3649.2017.02.030 | *Astragalus Membranacei Radix, Angelica Sinensis Radix, Ligustrum Lucidum Fructus, Scutellaria Barbata Herba, Curcumae Rhizoma* |
| 72 | 10.13863/j.issn1008-0805.2018.11.037 | *Rheum Palmatum Radix Et Rhizoma, Citrus Aurantium Fructus Immaturus, Magnoliae Officinalis Cortex, Atractylodes Lancea Rhizoma, Pericarpium Citri Reticulatae Viride, Pericarpium Citri Reticulatae Viride, Crataegi Fructus, Massa Medicata Fermentata* |
| 73 | 10.3969/j.issn.1008-8202.2006.01.01 | *Panax Ginseng Radix Et Rhizoma, Hominis Placenta, Atractylodes Macrocephalae Rhizoma, Poria Cocos, Angelica Sinensis Radix, Paeonia Lactiflora Radix Alba* |
| 74 | 10.13194/j.issn.1673-842x.2016.01.040 | *Euodia Rutaecarpa Fructus, Salvia Miltiorrhiza Radix Et Rhizoma, Asari Radix et Rhizoma, Sinapis Semen, Borneolum* |
| 75 | CNKI:CDFD2014000010000023 | *Poria Cocos, Polyporus, Alismatis Rhizoma, Atractylodes Macrocephalae Rhizoma, Cinnamomum Cassia Ramulus, Glycyrrhiza Uralensis Radix Et Rhizoma* |
| 76 | 10.13633/j.cnki.1000-3649.2024.09.029 | *Pseudostellariae Radix, Aquilariae Lignum Resinatum, Linderae Radix, Caryophylli Flos, Arecae Semen, Pinellia Ternata Rhizoma, Pericarpium Citri Reticulatae Viride, Poria Cocos, Folium Perillae, Zingiber Officinale Rhizoma Recens, Ziziphus Jujuba Fructus, Glycyrrhiza Uralensis Radix Et Rhizoma* |
| 77 | 10.16599/j.cnki.1672-1519.2024.05.022 | *Raphani Semen* |
| 78 | 10.16025/j.1674-1307.2023.04.005 | *Atractylodes Lancea Rhizoma, Aucklandia Lappa Radix, Arecae Semen, Angelica Dahurica Radix, Gleditsiae Sinensis Fructus* |
| 79 | 10.13633/j.cnki.1006-978X.2021.06.002 | *Euodia Rutaecarpa Fructus* |
| 80 | 10.14033/j.cnki.cfmr.2021.18.002 | *Lonicera Japonica Flos, Angelica Sinensis Radix, Rheum Palmatum Radix Et Rhizoma, Coptis Chinensis Rhizoma, Paeoniae Radix Rubra, Sophora Flavescens Radix, Momordicae Semen, Saposhnikovia Divaricata Radix, Phellodendron Chinense Cortex, Scutellaria Baicalensis Radix, Natrii Sulfas, Borneolum* |
| 81 | 10.16808/j.cnki.issn1003-7705.2021.03.039 | *Euodia Rutaecarpa Fructus, Rheum Palmatum Radix Et Rhizoma, Citrus Aurantium Fructus Immaturus, Magnoliae Officinalis Cortex* |
| 82 | 10.19664/j.cnki.1002-2392.2016.06.022 | *Prunus Persica Semen, Rheum Palmatum Radix Et Rhizoma, Cinnamomum Cassia Ramulus, Glycyrrhiza Uralensis Radix Et Rhizoma, Natrii Sulfas* |
| 83 | 10.3969/j.issn.1671-1246.2012.03.051 | *Codonopsis Pilosulae Radix, Atractylodes Macrocephalae Rhizoma, Aconitum Carmichaelii Radix Praeparata, Cinnamomum Cassia Ramulus, Cyperi Rhizoma, Rheum Palmatum Radix Et Rhizoma, Magnoliae Officinalis Cortex, Citrus Aurantium Fructus Immaturus, Paeoniae Radix Rubra, Prunus Persica Semen* |
| 84 | 10.3969/j.issn.1005-4561.2003.10.003 | *Astragalus Membranacei Radix, Testudinis Plastrum, Ligusticum Chuanxiong Rhizoma Rhizoma, Trionycis Carapax, Atractylodes Macrocephalae Rhizoma, Leonuri Herba, Crataegi Fructus* |
| 85 | 10.3969/j.issn.1007-614X.2007.10.038 | *Aucklandia Lappa Radix, Pericarpium Citri Reticulatae Viride, Magnoliae Officinalis Cortex, Crataegi Fructus, Rheum Palmatum Radix Et Rhizoma, Sennae Folium* |

*Identification number includes Registration Number, Drug approval number, Patent Number and DOI Number.

**STable.4. Top 20 herbs ranked by importance in random forest model (mean decrease accuracy).**

| **Herb** | **Mean Decrease Accuracy** | **Mean Decrease Gini** |
| --- | --- | --- |
| *Hedyotis Diffusa Herba* | 20.36 | 4.48 |
| *Astragalus Membranacei Radix* | 17.92 | 3.71 |
| *Curcumae Rhizoma* | 14.66 | 2.97 |
| *Coix Lacryma-jobi Semen* | 14.48 | 2.89 |
| *Atractylodes Macrocephalae Rhizoma* | 14.13 | 2.94 |
| *Sparganii Rhizoma* | 12.43 | 1.20 |
| *Fructus Akebiae* | 11.84 | 1.21 |
| *Curcumae Radix* | 9.80 | 0.92 |
| *Codonopsis Pilosulae Radix* | 8.93 | 1.78 |
| *Cinnamomum Cassia Ramulus* | 8.82 | 1.02 |
| *Bruceae Fructus* | 8.04 | 0.80 |
| *Ligusticum Chuanxiong Rhizoma* | 7.39 | 1.05 |
| *Cistanches Herba* | 7.05 | 0.39 |
| *Poria Cocos* | 6.99 | 1.65 |
| *Lonicera Japonica Flos* | 6.76 | 0.37 |
| *Ganoderma Lucidum* | 6.13 | 0.80 |
| *Scorpio* | 6.06 | 0.55 |
| *Zingiber Officinale Rhizoma Recens* | 5.69 | 0.48 |
| *Angelica Sinensis Radix* | 5.61 | 1.00 |
| *Angelica Dahurica Radix* | 5.52 | 0.47 |

**STable.5. Binding energies of molecular docking**

| **Target protein** | **Ligand** | **Binding energy (kcal/mol)** | **Ki (μM)** |
| --- | --- | --- | --- |
| PTGS1 | quercetin | -8.60 | <0.001 |
|  | kaempferol | -8.13 | <0.001 |
| PTGS2 | quercetin | -9.25 | <0.001 |
|  | kaempferol | -8.87 | <0.001 |
| NCOA2 | quercetin | -7.65 | <0.001 |
|  | kaempferol | -7.54 | <0.001 |
| HSP90AA1 | quercetin | -8.63 | <0.001 |
|  | kaempferol | -8.51 | <0.001 |
| PRSS1 | quercetin | -7.47 | <0.001 |
|  | kaempferol | -7.13 | <0.001 |
| GABRA1 | quercetin | -8.18 | <0.001 |
|  | kaempferol | -7.67 | <0.001 |

**Stable.6. Results of molecular dynamics simulation**

| **Protein-ligand complex** | **RMSD (nm)** | **Rg(nm)** | **Hydrogen bonds** | **Hydrophobic contact** |
| --- | --- | --- | --- | --- |
| PTGS1-quercetin | 0.216 | 1.789 | 3 | 5 |
| PTGS1-kaempferol | 0.230 | 1.821 | 2 | 7 |
| PTGS2-quercetin | 0.224 | 1.795 | 2 | 5 |
| PTGS2-kaempferol | 0.225 | 1.795 | 4 | 7 |
| NCOA2-quercetin | 0.215 | 1.791 | 4 | 5 |
| NCOA2-kaempferol | 0.231 | 1.800 | 4 | 3 |
| HSP90AA1-quercetin | 0.226 | 1.781 | 4 | 3 |
| HSP90AA1-kaempferol | 0.219 | 1.790 | 4 | 4 |
| PRSS1-quercetin | 0.216 | 1.794 | 5 | 7 |
| PRSS1-kaempferol | 0.229 | 1.801 | 4 | 5 |
| GABRA1-quercetin | 0.229 | 1.827 | 5 | 4 |
| GABRA1-kaempferol | 0.236 | 1.806 | 2 | 4 |
