# Supplementary figures and images for: Unveiling medication patterns in traditional Chinese medicine for the prevention of colorectal cancer recurrence: from potential combinations to validation of components and targets
Source: Chin Med. 2026 Jun 4;21:160. doi: 10.1186/s13020-026-01438-5 (PMC13235114; doi:10.1186/s13020-026-01438-5)

Fig 8 A


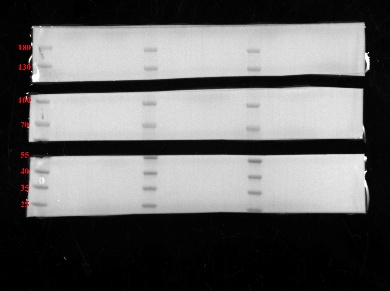

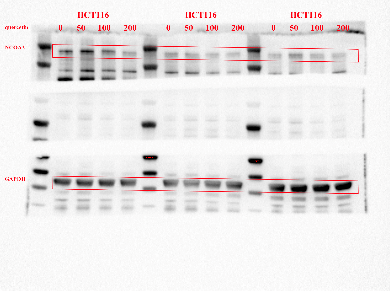

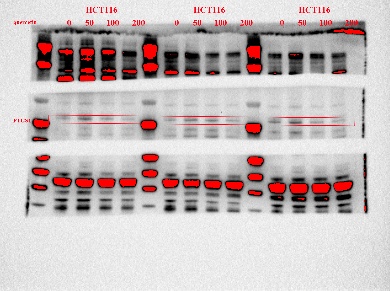


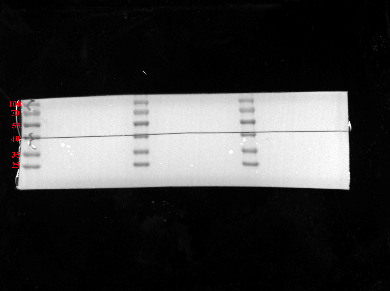

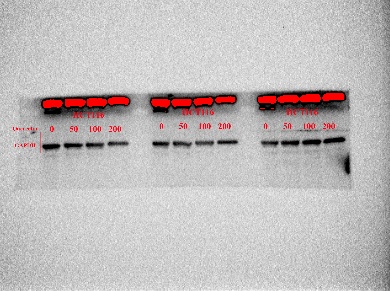

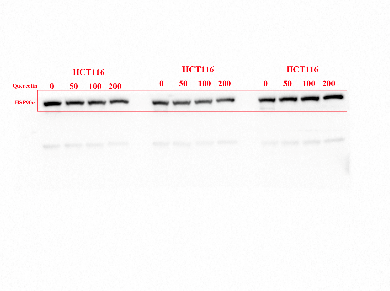


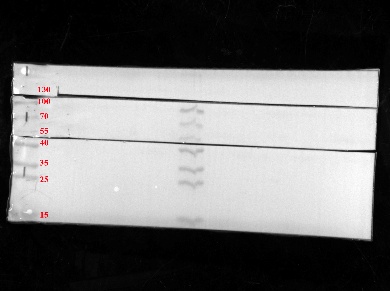

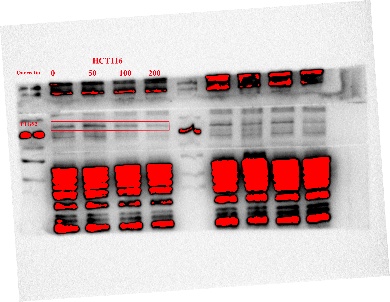

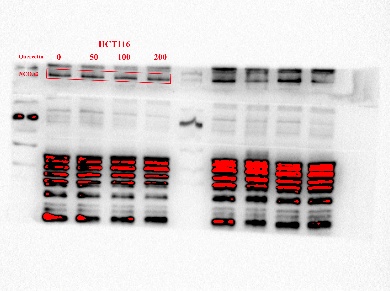


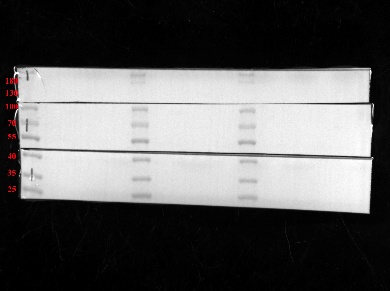

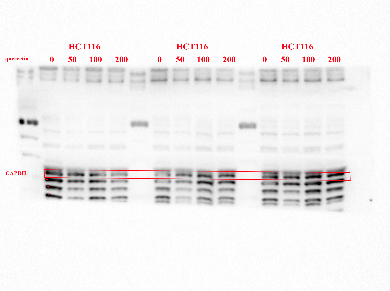

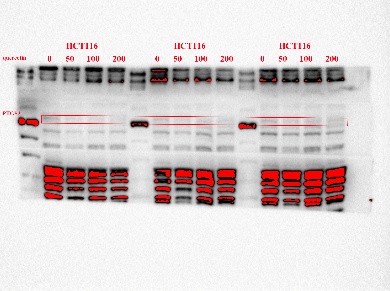


Fig 8 B


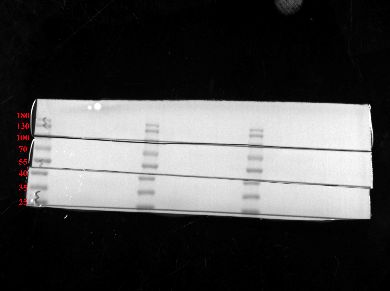

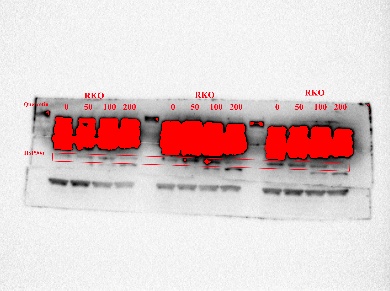

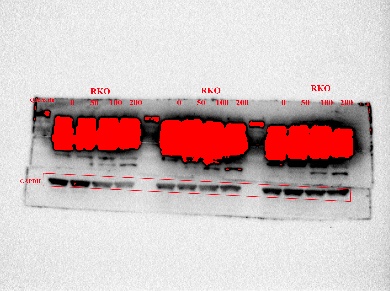

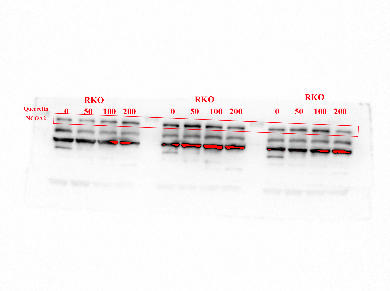


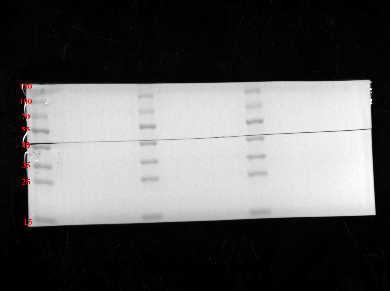

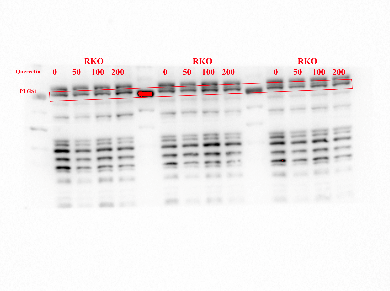

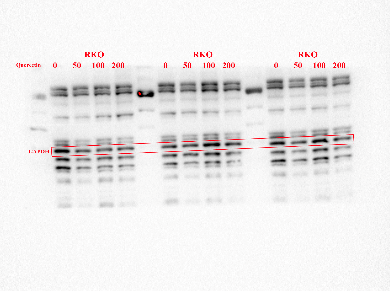


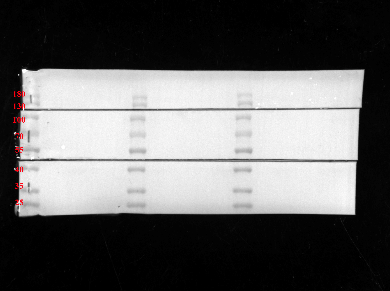

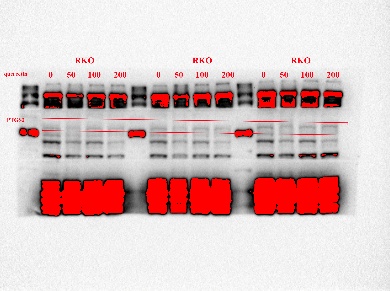

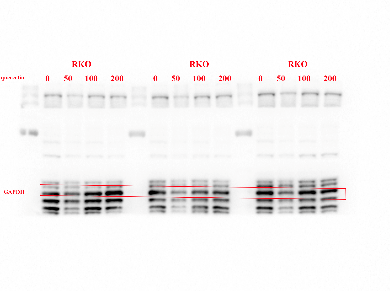


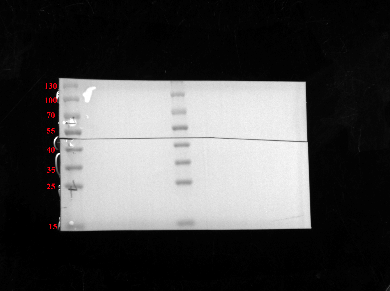

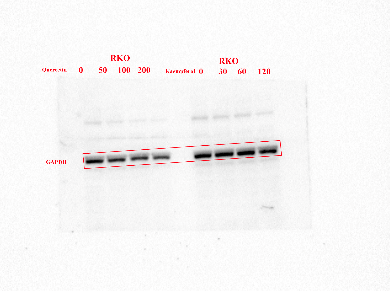

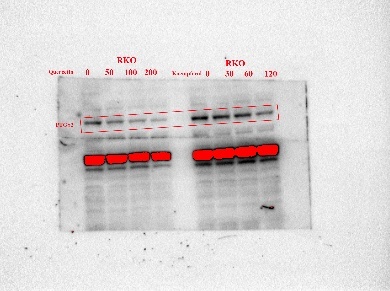


Fig 8 C


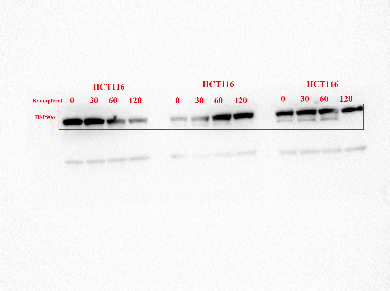

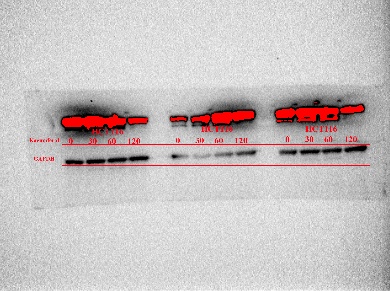

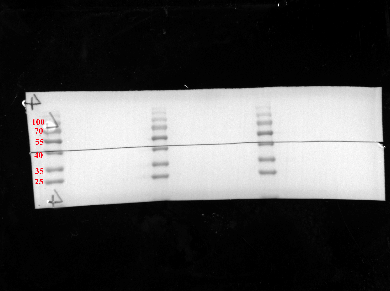


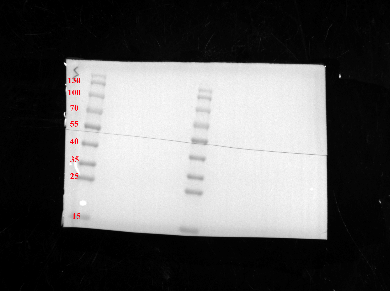

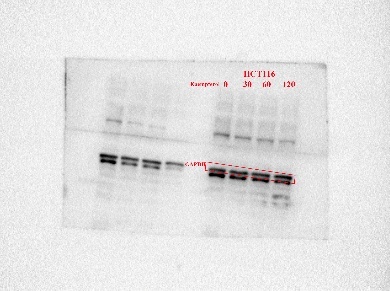

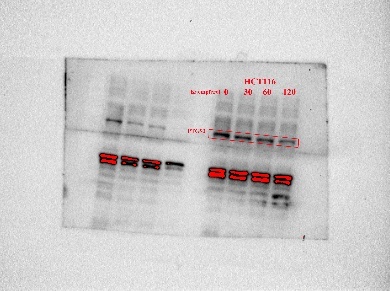


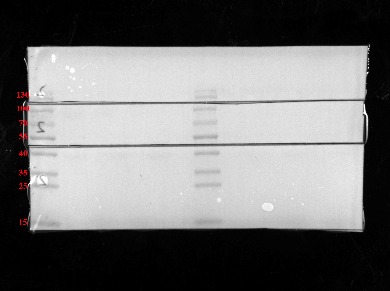

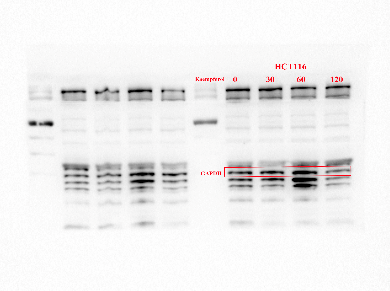

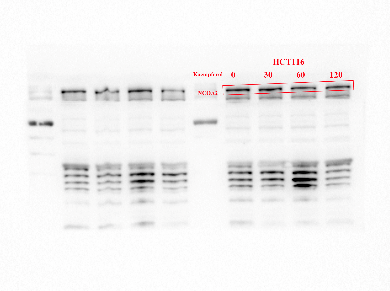


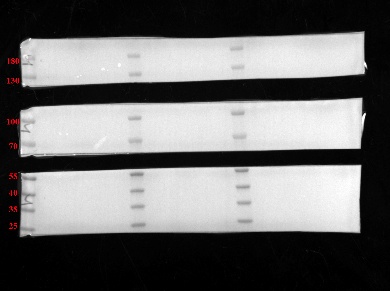

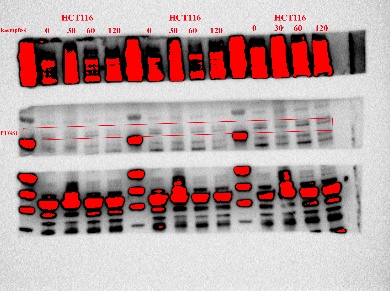

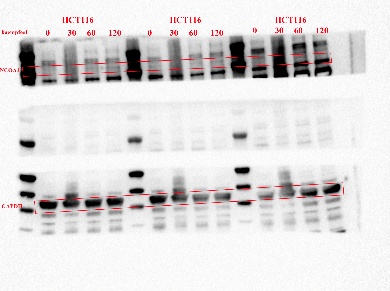


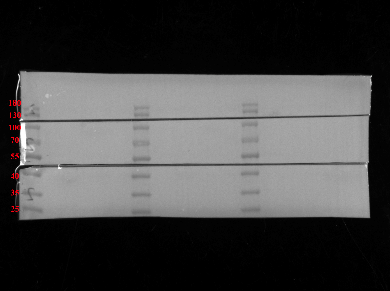

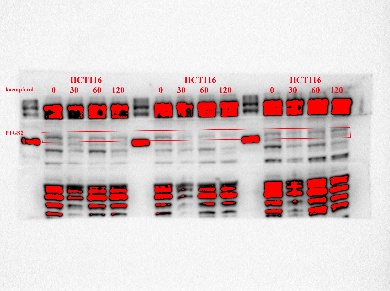

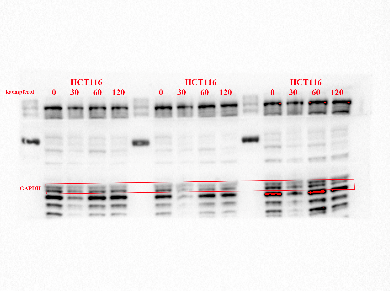


Fig 8 D


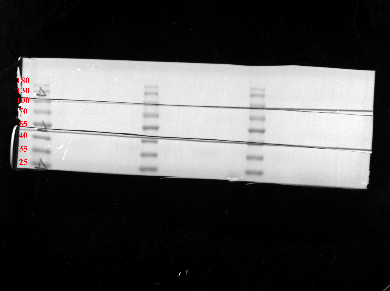

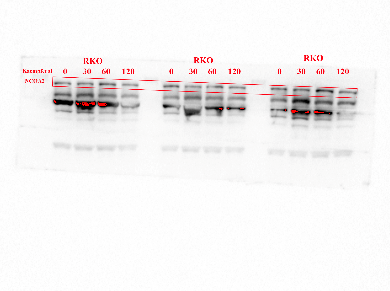

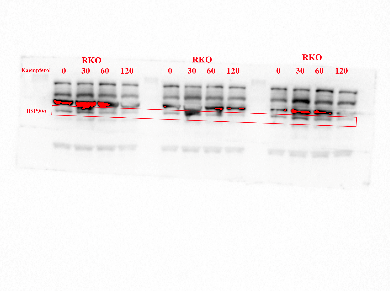

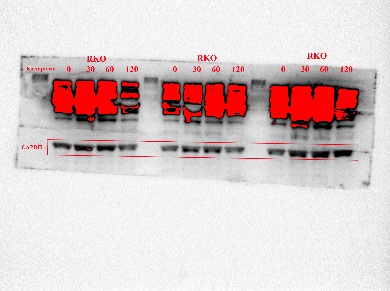


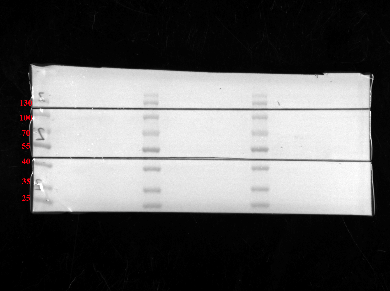

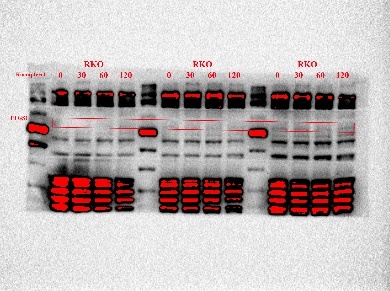

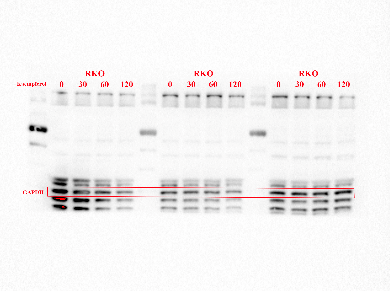


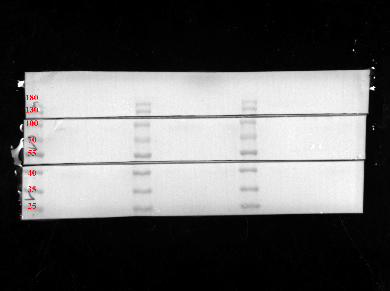

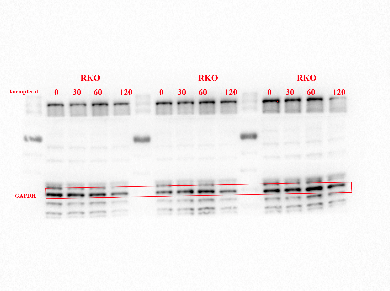

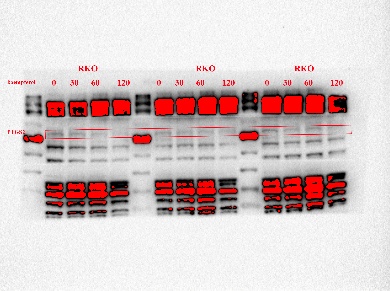

Supplement: Supplementary file 2 — Supplementary Material 2 [file 13020_2026_1438_MOESM2_ESM.docx]
