# Supplementary material for: Unveiling medication patterns in traditional Chinese medicine for the prevention of colorectal cancer recurrence: from potential combinations to validation of components and targets
Source: Chin Med. 2026 Jun 4;21:160. doi: 10.1186/s13020-026-01438-5 (PMC13235114; doi:10.1186/s13020-026-01438-5)
Supplement: Supplementary file 3 — Supplementary Material 3 [file 13020_2026_1438_MOESM3_ESM.docx]

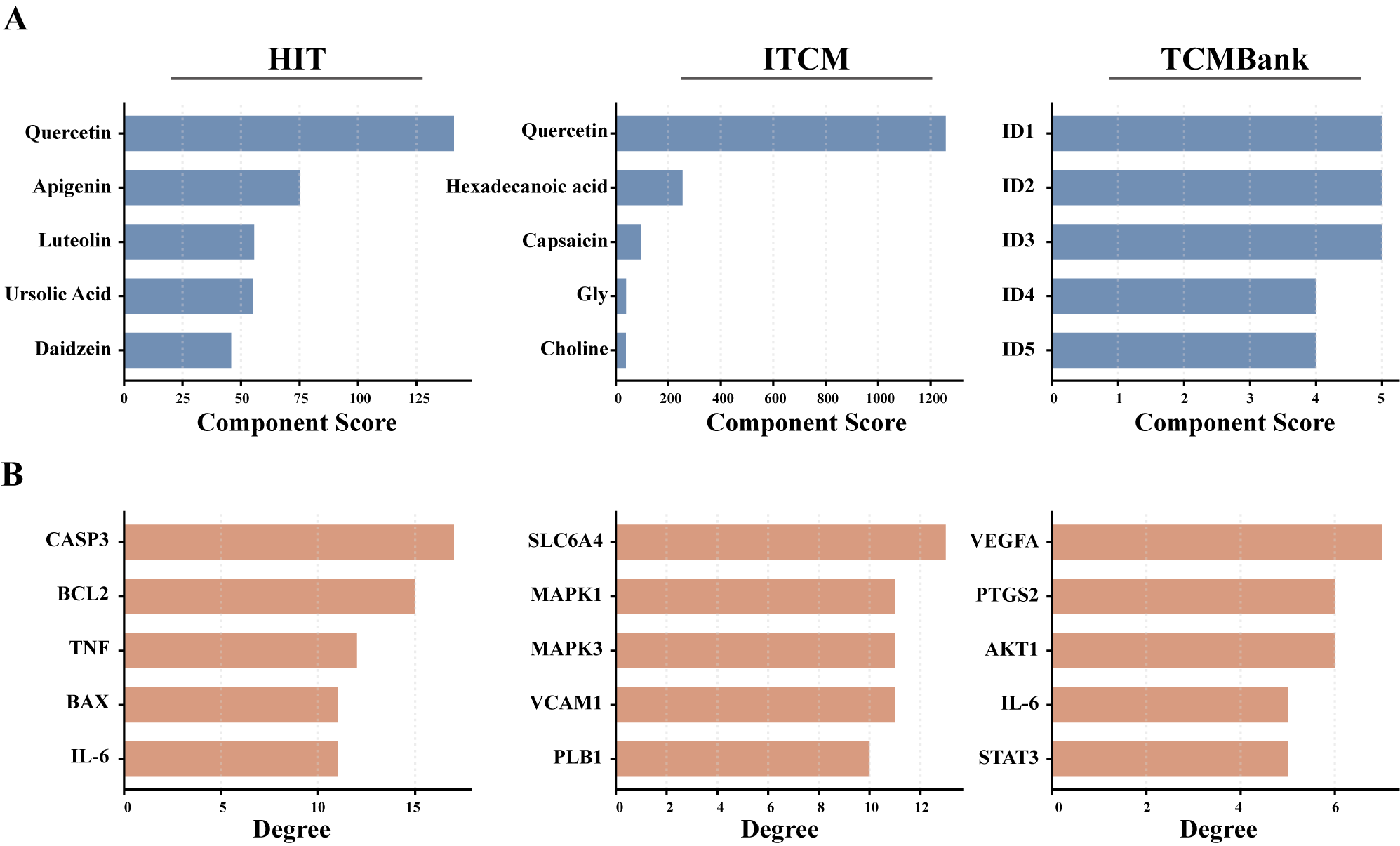


SFigure.1 Core components and targets of HIT, ITCM, and TCMBank databases. **A** The top five active ingredients in compatible TCM ranked by degree. In the TCMBank database, IDs 1-5 correspond to TCMMBANKIN022441, TCMBANKIN041912, TCMBANKIN051993, TCMBANKIN006698, and TCMBANKIN019481, respectively. **B** The top five target proteins in compatible TCM ranked by degree.


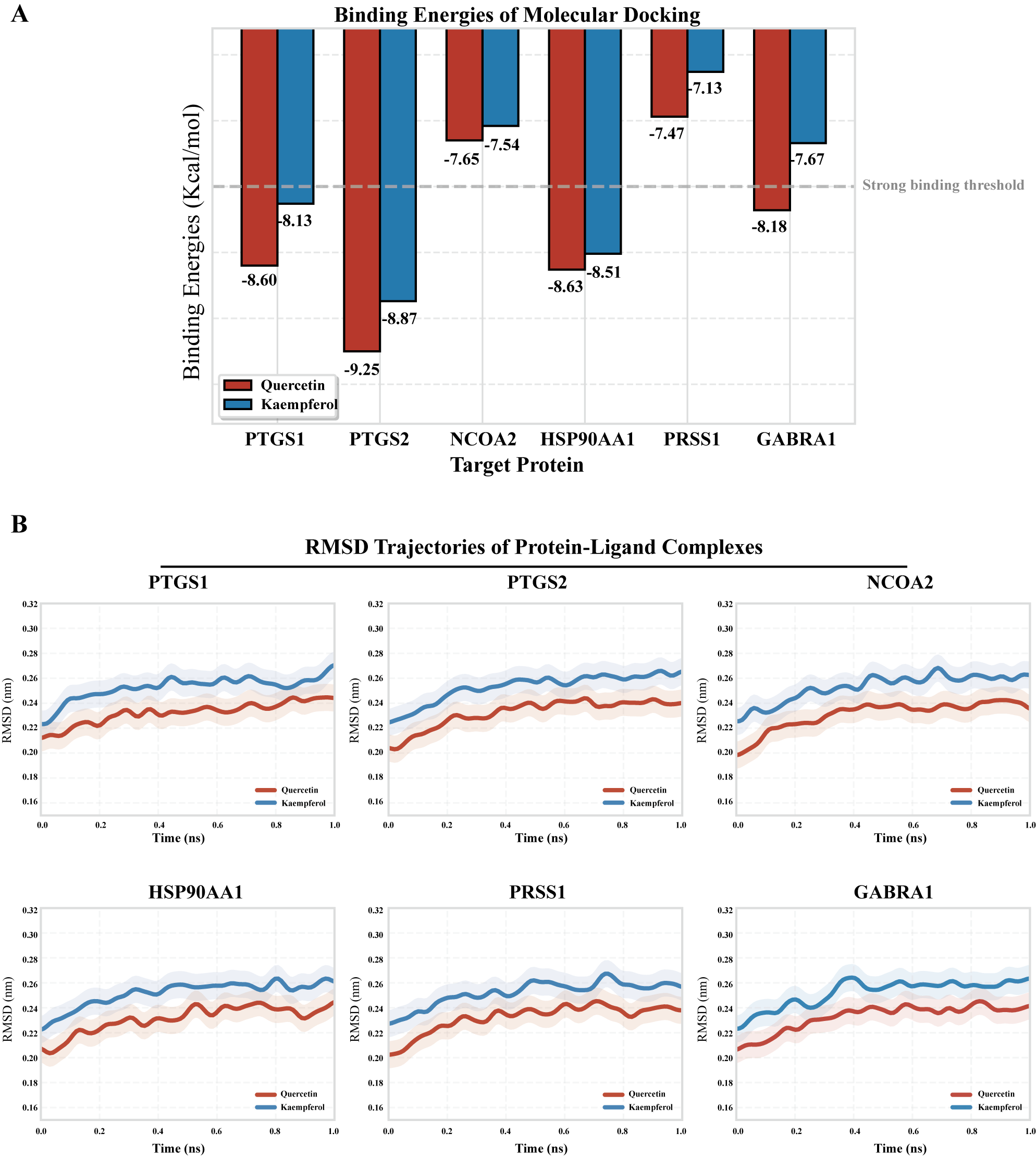


SFigure.2 The molecular docking and molecular dynamics simulation. **A** Binding energy of molecular docking of quercetin and kaempferol with target molecules. **B** RMSD trajectories of protein-ligand complexes.


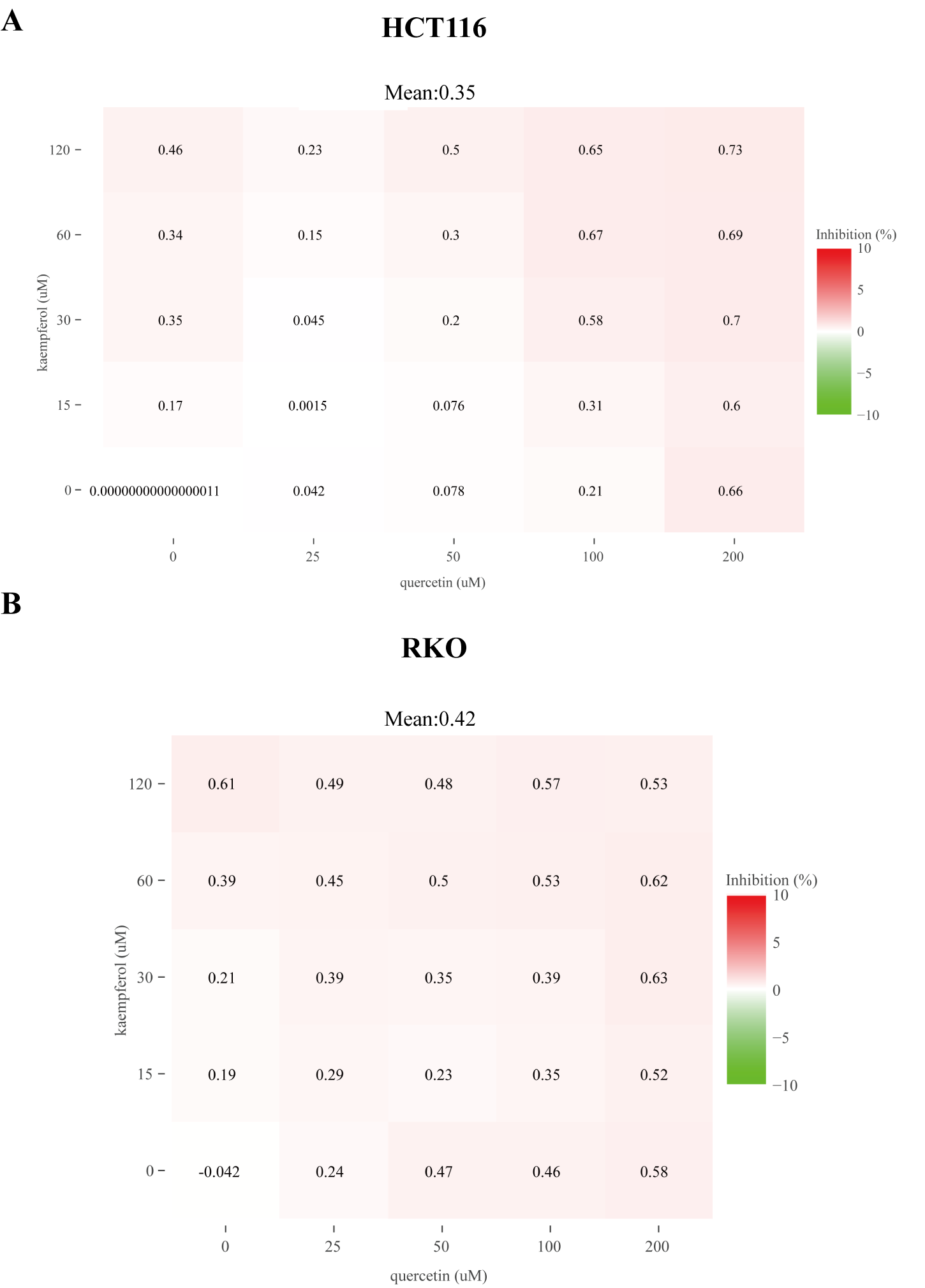


SFigure.3 Combination experiment of quercetin and kaempferol. **A** Combination experiment in HCT116 cells. **B** Combination experiment in RKO cells.
